# Supplementary material for: Infrared Spectra of Small Radicals for Exoplanetary Spectroscopy: OH, NH, CN and CH: The State of Current Knowledge
Source: Molecules. 2023 Apr 11;28(8):3362. doi: 10.3390/molecules28083362 (PMC10143568; doi:10.3390/molecules28083362)
Supplement: Supplementary file 1 [file molecules-28-03362-s001.zip › molecules-2303650-supplementary.pdf]

Supplementary Information for
"Infrared Spectra of Small Radicals for Exoplanetary Spectroscopy: OH, NH, CN and CH. The State of Current Knowledge"

This supplementary information provides line lists for all our experimentally observed emission lines of OH, NH, CH, CN radicals. Aside the experimental line positions together with spectroscopic notations, we also provide a comparison with theoretically predicted line positions. These predictions were either taken from the HITRAN database or generated by use of MoLLIST database.

Note: Experimental line positions are entirely original and not compiled from other sources. The estimated accuracy for measured line positions is close to the Doppler width of the lines, between 0.001-0.003 cm^{-1} in the infrared spectral range.

Legend:

ν_{exp} – experimentally observed wavenumber in cm^{-1} units

ν_{the} – theoretically predicted wavenumber in cm^{-1} units (HITRAN or MoLLIST)

GUQ – global upper quanta (spectral notation of the upper state together with the spin-component notation)

GLQ – global lower quanta (spectral notation of the lower state together with the spin-component notation)

v' – upper vibrational quantum number

v'' – lower vibrational quantum number

J' – upper rotational quantum number

J'' – lower rotational quantum number

N' – further upper rotational quantum number

N'' – further lower rotational quantum number

branch – spectral 'PQR' notation of selected emission line

F' – upper quantum number (F = I + J, where I is a net nuclear spin – F therefore corresponds to a total angular momentum of an atom, arising from the coupling of a magnetic moment of an electron and a nuclear magnetic moment)

F'' – lower quantum number

Table S1: Emission lines of OH radical in the $X^2\Pi$ ground state between 1800-3500 cm^{-1}

ν_{exp} (cm^{-1})	ν_{the} (cm^{-1})	GUQ	GLQ	v'	v''	J'	J''
1800.446	1800.4439	$X_{3/2}$	$X_{1/2}$	2	1	31.5	31.5
1809.623	1809.6284	$X_{1/2}$	$X_{1/2}$	3	2	30.5	29.5
1815.217	1815.2130	$X_{3/2}$	$X_{3/2}$	2	1	33.5	32.5
1830.622	1830.6216	$X_{3/2}$	$X_{1/2}$	10	9	4.5	4.5
1831.194	1831.1974	$X_{1/2}$	$X_{1/2}$	11	10	3.5	3.5
1831.900	1831.9033	$X_{3/2}$	$X_{1/2}$	9	8	10.5	10.5
1841.066	1841.0623	$X_{1/2}$	$X_{1/2}$	9	8	12.5	11.5
1845.863	1845.8676	$X_{1/2}$	$X_{1/2}$	11	10	0.5	0.5
1852.695	1852.6929	$X_{1/2}$	$X_{1/2}$	10	9	7.5	6.5
1853.482	1853.4827	$X_{3/2}$	$X_{1/2}$	5	4	23.5	23.5
1857.031	1857.0251	$X_{3/2}$	$X_{1/2}$	4	3	17.5	16.5
1861.155	1861.1582	$X_{3/2}$	$X_{1/2}$	7	6	17.5	17.5
1873.392	1873.3963	$X_{1/2}$	$X_{1/2}$	2	1	31.5	30.5
1874.106	1874.1051	$X_{3/2}$	$X_{3/2}$	8	7	16.5	15.5
1874.954	1874.9583	$X_{1/2}$	$X_{3/2}$	12	11	4.5	3.5
1876.410	1876.4114	$X_{1/2}$	$X_{3/2}$	1	1	33.5	32.5
1882.668	1882.6714	$X_{3/2}$	$X_{1/2}$	5	4	15.5	14.5
1892.140	1892.1423	$X_{1/2}$	$X_{1/2}$	6	5	21.5	20.5
1896.451	1896.4507	$X_{1/2}$	$X_{3/2}$	0	0	31.5	30.5
1896.710	1896.7146	$X_{3/2}$	$X_{1/2}$	3	2	18.5	17.5
1903.463	1903.4661	$X_{3/2}$	$X_{1/2}$	3	2	44.5	43.5
1911.094	1911.0901	$X_{3/2}$	$X_{3/2}$	11	10	3.5	2.5
1914.208	1914.2077	$X_{3/2}$	$X_{1/2}$	2	1	29.5	29.5
1915.376	1915.3717	$X_{3/2}$	$X_{3/2}$	9	8	11.5	10.5
1917.164	1917.1681	$X_{3/2}$	$X_{1/2}$	1	0	31.5	31.5
1917.589	1917.5893	$X_{3/2}$	$X_{1/2}$	10	9	1.5	0.5
1920.084	1920.0807	$X_{1/2}$	$X_{1/2}$	8	7	14.5	13.5
1920.720	1920.7181	$X_{1/2}$	$X_{1/2}$	9	8	10.5	9.5
1923.072	1923.0681	$X_{1/2}$	$X_{1/2}$	10	9	11.5	11.5
1929.481	1929.4854	$X_{1/2}$	$X_{1/2}$	11	10	4.5	3.5
1931.048	1931.0476	$X_{3/2}$	$X_{3/2}$	3	2	43.5	43.5
1931.351	1931.3462	$X_{3/2}$	$X_{1/2}$	10	9	2.5	1.5
1933.067	1933.0646	$X_{3/2}$	$X_{1/2}$	9	8	7.5	7.5
1934.787	1934.7906	$X_{3/2}$	$X_{1/2}$	4	3	24.5	24.5
1936.178	1936.1774	$X_{1/2}$	$X_{3/2}$	3	2	45.5	44.5
1937.176	1937.1710	$X_{1/2}$	$X_{3/2}$	2	1	48.5	47.5
1937.520	1937.5144	$X_{3/2}$	$X_{3/2}$	10	9	5.5	4.5
1938.309	1938.3107	$X_{1/2}$	$X_{1/2}$	11	10	14.5	13.5
1945.384	1945.3854	$X_{1/2}$	$X_{3/2}$	2	2	38.5	37.5
1947.848	1947.8485	$X_{3/2}$	$X_{3/2}$	11	10	6.5	5.5
1954.325	1954.3230	$X_{1/2}$	$X_{3/2}$	12	11	9.5	8.5
1958.558	1958.5616	$X_{1/2}$	$X_{1/2}$	9	8	9.5	8.5
1959.418	1959.4236	$X_{1/2}$	$X_{3/2}$	2	2	39.5	38.5

Table S1 (continued)

ν_{exp} (cm^{-1})	ν_{the} (cm^{-1})	GUQ	GLQ	v'	v''	J'	J''
1961.094	1961.0974	$X_{3/2}$	$X_{1/2}$	6	5	18.5	18.5
1961.361	1961.3609	$X_{3/2}$	$X_{1/2}$	6	5	12.5	11.5
1962.808	1962.8122	$X_{3/2}$	$X_{1/2}$	5	4	14.5	13.5
1963.790	1963.7875	$X_{1/2}$	$X_{1/2}$	8	7	13.5	12.5
1965.611	1965.6111	$X_{3/2}$	$X_{3/2}$	10	9	4.5	3.5
1966.374	1966.3764	$X_{1/2}$	$X_{1/2}$	11	10	9.5	8.5
1966.564	1966.5696	$X_{1/2}$	$X_{1/2}$	11	10	10.5	9.5
1976.236	1976.2361	$X_{1/2}$	$X_{1/2}$	10	9	8.5	8.5
1977.699	1977.6944	$X_{1/2}$	$X_{1/2}$	10	9	8.5	8.5
1979.602	1979.5993	$X_{1/2}$	$X_{3/2}$	2	2	41.5	40.5
1982.445	1982.4416	$X_{1/2}$	$X_{3/2}$	0	0	32.5	31.5
1987.580	1987.5831	$X_{3/2}$	$X_{3/2}$	6	5	20.5	19.5
1988.381	1988.3768	$X_{3/2}$	$X_{3/2}$	9	8	9.5	8.5
1988.920	1988.9222	$X_{3/2}$	$X_{3/2}$	9	8	9.5	8.5
1992.404	1992.4086	$X_{1/2}$	$X_{1/2}$	10	9	7.5	7.5
1993.934	1993.9378	$X_{1/2}$	$X_{1/2}$	9	8	16.5	16.5
1994.580	1994.5761	$X_{3/2}$	$X_{1/2}$	9	8	3.5	2.5
1995.710	1995.7124	$X_{1/2}$	$X_{1/2}$	9	8	8.5	7.5
2002.359	2002.3557	$X_{3/2}$	$X_{3/2}$	10	9	7.5	7.5
2005.428	2005.4248	$X_{1/2}$	$X_{1/2}$	10	9	6.5	6.5
2006.945	2006.9497	$X_{3/2}$	$X_{3/2}$	4	3	25.5	24.5
2013.393	2013.3908	$X_{3/2}$	$X_{1/2}$	9	8	4.5	4.5
2018.329	2018.3278	$X_{1/2}$	$X_{3/2}$	11	10	4.5	4.5
2018.486	2018.4820	$X_{1/2}$	$X_{3/2}$	11	10	4.5	4.5
2018.958	2018.9637	$X_{3/2}$	$X_{3/2}$	9	8	16.5	16.5
2022.899	2022.9029	$X_{1/2}$	$X_{3/2}$	11	10	6.5	6.5
2027.037	2027.0364	$X_{3/2}$	$X_{1/2}$	2	1	27.5	27.5
2028.469	2028.4709	$X_{1/2}$	$X_{3/2}$	1	1	37.5	36.5
2029.116	2029.1208	$X_{1/2}$	$X_{1/2}$	9	8	15.5	15.5
2032.290	2032.2947	$X_{3/2}$	$X_{1/2}$	5	4	13.5	12.5
2033.261	2033.2613	$X_{3/2}$	$X_{3/2}$	10	9	4.5	4.5
2038.596	2038.6013	$X_{3/2}$	$X_{1/2}$	7	6	13.5	13.5
2041.394	2041.3904	$X_{3/2}$	$X_{3/2}$	8	7	12.5	11.5
2042.213	2042.2170	$X_{1/2}$	$X_{3/2}$	1	1	48.5	47.5
2046.502	2046.4982	$X_{1/2}$	$X_{1/2}$	1	0	47.5	47.5
2052.710	2052.7092	$X_{1/2}$	$X_{3/2}$	1	1	46.5	45.5
2053.434	2053.4338	$X_{1/2}$	$X_{1/2}$	1	0	46.5	46.5
2062.792	2062.7909	$X_{3/2}$	$X_{1/2}$	3	2	24.5	24.5
2066.788	2066.7908	$X_{1/2}$	$X_{1/2}$	9	8	6.5	5.5
2072.637	2072.6340	$X_{3/2}$	$X_{1/2}$	9	8	12.5	11.5
2076.034	2076.0376	$X_{1/2}$	$X_{1/2}$	7	6	14.5	13.5
2077.771	2077.7669	$X_{3/2}$	$X_{1/2}$	8	7	5.5	4.5
2078.649	2078.6480	$X_{3/2}$	$X_{3/2}$	5	4	21.5	20.5

Table S1 (continued)

ν_{exp} (cm^{-1})	ν_{the} (cm^{-1})	GUQ	GLQ	v'	v''	J'	J''
2081.875	2081.8743	$X_{1/2}$	$X_{1/2}$	5	4	20.5	19.5
2083.407	2083.4077	$X_{3/2}$	$X_{3/2}$	6	5	18.5	17.5
2089.220	2089.2262	$X_{3/2}$	$X_{3/2}$	9	8	6.5	5.5
2090.101	2090.1048	$X_{1/2}$	$X_{3/2}$	9	8	16.5	15.5
2093.855	2093.8595	$X_{3/2}$	$X_{3/2}$	4	3	37.5	37.5
2096.806	2096.8049	$X_{3/2}$	$X_{1/2}$	1	0	46.5	45.5
2099.631	2099.6357	$X_{3/2}$	$X_{3/2}$	2	1	28.5	27.5
2100.434	2100.4365	$X_{1/2}$	$X_{1/2}$	9	8	5.5	4.5
2100.495	2100.5003	$X_{1/2}$	$X_{1/2}$	9	8	5.5	4.5
2101.576	2101.5764	$X_{1/2}$	$X_{3/2}$	1	1	42.5	41.5
2102.034	2102.0287	$X_{3/2}$	$X_{3/2}$	2	1	28.5	27.5
2103.778	2103.7782	$X_{1/2}$	$X_{1/2}$	3	2	40.5	40.5
2105.372	2105.3676	$X_{3/2}$	$X_{1/2}$	9	8	1.5	0.5
2108.949	2108.9501	$X_{3/2}$	$X_{3/2}$	3	2	40.5	40.5
2112.280	2112.2818	$X_{3/2}$	$X_{3/2}$	4	3	23.5	22.5
2113.806	2113.8113	$X_{1/2}$	$X_{1/2}$	4	3	22.5	21.5
2117.259	2117.2628	$X_{3/2}$	$X_{3/2}$	2	1	44.5	44.5
2118.693	2118.6924	$X_{3/2}$	$X_{1/2}$	3	1	44.5	43.5
2121.644	2121.6466	$X_{1/2}$	$X_{3/2}$	10	9	5.5	4.5
2124.783	2124.7869	$X_{3/2}$	$X_{1/2}$	8	7	18.5	17.5
2126.461	2126.4551	$X_{1/2}$	$X_{3/2}$	0	0	37.5	36.5
2128.449	2128.4549	$X_{3/2}$	$X_{3/2}$	5	4	20.5	19.5
2130.197	2130.1966	$X_{3/2}$	$X_{3/2}$	6	5	17.5	16.5
2135.207	2135.2035	$X_{1/2}$	$X_{1/2}$	9	8	10.5	10.5
2140.712	2140.7082	$X_{3/2}$	$X_{1/2}$	4	3	20.5	20.5
2141.723	2141.7187	$X_{1/2}$	$X_{1/2}$	10	9	4.5	3.5
2148.794	2148.7956	$X_{3/2}$	$X_{3/2}$	9	8	4.5	3.5
2156.356	2156.3581	$X_{1/2}$	$X_{1/2}$	10	9	5.5	4.5
2160.794	2160.7936	$X_{1/2}$	$X_{1/2}$	3	2	39.5	39.5
2160.991	2160.9896	$X_{3/2}$	$X_{1/2}$	8	7	5.5	5.5
2163.779	2163.7839	$X_{1/2}$	$X_{1/2}$	8	7	8.5	7.5
2170.339	2170.3335	$X_{1/2}$	$X_{1/2}$	1	0	28.5	27.5
2171.124	2171.1248	$X_{1/2}$	$X_{3/2}$	0	0	47.5	46.5
2177.009	2177.0139	$X_{3/2}$	$X_{3/2}$	2	1	43.5	43.5
2178.593	2178.5963	$X_{1/2}$	$X_{3/2}$	9	8	12.5	11.5
2179.771	2179.7761	$X_{1/2}$	$X_{1/2}$	5	4	18.5	17.5
2183.221	2183.2194	$X_{3/2}$	$X_{3/2}$	10	9	8.5	7.5
2190.503	2190.5017	$X_{3/2}$	$X_{3/2}$	10	9	9.5	8.5
2192.833	2192.8334	$X_{3/2}$	$X_{3/2}$	9	8	7.5	7.5
2198.935	2198.9365	$X_{3/2}$	$X_{3/2}$	10	9	11.5	10.5
2210.096	2210.0951	$X_{3/2}$	$X_{1/2}$	1	0	17.5	16.5
2214.699	2214.7004	$X_{1/2}$	$X_{1/2}$	2	1	25.5	24.5
2215.091	2215.0889	$X_{1/2}$	$X_{3/2}$	10	9	1.5	1.5

Table S1 (continued)

ν_{exp} (cm^{-1})	ν_{the} (cm^{-1})	GUQ	GLQ	v'	v''	J'	J''
2218.525	2218.5190	$X_{1/2}$	$X_{1/2}$	8	7	15.5	15.5
2225.863	2225.8587	$X_{1/2}$	$X_{3/2}$	10	9	14.5	14.5
2228.441	2228.4435	$X_{1/2}$	$X_{1/2}$	5	4	17.5	16.5
2230.386	2230.3796	$X_{3/2}$	$X_{1/2}$	4	3	34.5	33.5
2232.192	2232.1884	$X_{3/2}$	$X_{1/2}$	5	4	10.5	9.5
2232.708	2232.7070	$X_{3/2}$	$X_{3/2}$	9	8	2.5	2.5
2233.209	2233.2138	$X_{1/2}$	$X_{1/2}$	2	1	40.5	40.5
2233.383	2233.3803	$X_{1/2}$	$X_{1/2}$	9	8	1.5	1.5
2238.938	2238.9376	$X_{1/2}$	$X_{3/2}$	10	9	6.5	6.5
2239.138	2239.1421	$X_{3/2}$	$X_{1/2}$	8	7	1.5	1.5
2244.569	2244.5728	$X_{3/2}$	$X_{1/2}$	5	4	15.5	15.5
2245.231	2245.2302	$X_{3/2}$	$X_{3/2}$	4	3	35.5	35.5
2255.009	2255.0064	$X_{3/2}$	$X_{1/2}$	3	2	37.5	36.5
2256.635	2256.6336	$X_{1/2}$	$X_{3/2}$	7	6	23.5	22.5
2268.556	2268.5561	$X_{1/2}$	$X_{1/2}$	3	2	37.5	37.5
2279.486	2279.4883	$X_{1/2}$	$X_{1/2}$	8	7	12.5	12.5
2283.543	2283.5467	$X_{1/2}$	$X_{1/2}$	1	0	26.5	25.5
2285.533	2285.5353	$X_{1/2}$	$X_{1/2}$	1	0	26.5	25.5
2286.062	2286.0652	$X_{1/2}$	$X_{1/2}$	6	5	24.5	24.5
2292.333	2292.3276	$X_{3/2}$	$X_{3/2}$	9	8	2.5	1.5
2300.178	2300.1787	$X_{3/2}$	$X_{3/2}$	3	2	22.5	21.5
2301.345	2301.3493	$X_{1/2}$	$X_{1/2}$	3	2	21.5	20.5
2304.614	2304.6165	$X_{3/2}$	$X_{3/2}$	7	6	19.5	19.5
2308.138	2308.1370	$X_{1/2}$	$X_{1/2}$	7	6	18.5	18.5
2308.420	2308.4250	$X_{3/2}$	$X_{1/2}$	1	0	16.5	15.5
2309.386	2309.3909	$X_{1/2}$	$X_{3/2}$	4	3	35.5	34.5
2310.184	2310.1848	$X_{1/2}$	$X_{3/2}$	1	0	45.5	44.5
2313.790	2313.7878	$X_{1/2}$	$X_{1/2}$	6	5	12.5	11.5
2314.760	2314.7646	$X_{3/2}$	$X_{1/2}$	8	7	7.5	6.5
2316.056	2316.0614	$X_{3/2}$	$X_{1/2}$	8	7	4.5	3.5
2318.716	2318.7144	$X_{1/2}$	$X_{1/2}$	4	3	18.5	17.5
2319.467	2319.4705	$X_{3/2}$	$X_{1/2}$	7	6	18.5	17.5
2319.603	2319.5981	$X_{1/2}$	$X_{1/2}$	3	2	36.5	36.5
2332.894	2332.8894	$X_{3/2}$	$X_{3/2}$	7	6	18.5	18.5
2338.193	2338.1974	$X_{1/2}$	$X_{1/2}$	8	7	3.5	2.5
2339.771	2339.7726	$X_{3/2}$	$X_{1/2}$	13	11	11.5	10.5
2345.979	2345.9818	$X_{1/2}$	$X_{3/2}$	9	8	3.5	2.5
2349.494	2349.4948	$X_{1/2}$	$X_{3/2}$	7	6	20.5	19.5
2358.180	2358.1743	$X_{1/2}$	$X_{3/2}$	2	1	41.5	40.5
2358.392	2358.3866	$X_{3/2}$	$X_{1/2}$	3	2	12.5	11.5
2367.179	2367.1840	$X_{3/2}$	$X_{3/2}$	5	4	15.5	14.5
2369.847	2369.8492	$X_{1/2}$	$X_{1/2}$	8	7	2.5	1.5
2375.319	2375.3146	$X_{3/2}$	$X_{3/2}$	9	8	7.5	6.5

Table S1 (continued)

ν_{exp} (cm^{-1})	ν_{the} (cm^{-1})	GUQ	GLQ	v'	v''	J'	J''
2378.676	2378.6780	$X_{1/2}$	$X_{1/2}$	2	1	22.5	21.5
2379.333	2379.3372	$X_{3/2}$	$X_{3/2}$	2	1	23.5	22.5
2380.327	2380.3277	$X_{1/2}$	$X_{1/2}$	2	1	22.5	21.5
2380.870	2380.8650	$X_{3/2}$	$X_{1/2}$	1	0	41.5	40.5
2384.806	2384.8071	$X_{3/2}$	$X_{3/2}$	7	6	16.5	16.5
2393.044	2393.0495	$X_{3/2}$	$X_{3/2}$	6	5	11.5	10.5
2394.156	2394.1582	$X_{1/2}$	$X_{1/2}$	9	8	8.5	7.5
2398.972	2398.9707	$X_{3/2}$	$X_{3/2}$	1	0	25.5	24.5
2399.886	2399.8871	$X_{1/2}$	$X_{1/2}$	1	0	40.5	40.5
2400.427	2400.4298	$X_{3/2}$	$X_{1/2}$	3	2	34.5	33.5
2402.911	2402.9088	$X_{3/2}$	$X_{3/2}$	3	2	20.5	19.5
2405.811	2405.8090	$X_{1/2}$	$X_{3/2}$	9	8	1.5	1.5
2406.090	2406.0919	$X_{1/2}$	$X_{1/2}$	3	2	19.5	18.5
2407.510	2407.5085	$X_{1/2}$	$X_{1/2}$	8	7	2.5	2.5
2412.344	2412.3496	$X_{1/2}$	$X_{3/2}$	10	9	7.5	6.5
2412.615	2412.6165	$X_{3/2}$	$X_{3/2}$	5	4	14.5	13.5
2413.603	2413.6056	$X_{1/2}$	$X_{3/2}$	10	9	7.5	6.5
2414.404	2414.4049	$X_{3/2}$	$X_{1/2}$	6	5	21.5	20.5
2415.228	2415.2299	$X_{3/2}$	$X_{3/2}$	4	3	17.5	16.5
2416.609	2416.6138	$X_{3/2}$	$X_{3/2}$	9	8	14.5	13.5
2416.859	2416.8567	$X_{1/2}$	$X_{1/2}$	5	4	13.5	12.5
2421.341	2421.3364	$X_{3/2}$	$X_{3/2}$	3	2	35.5	35.5
2423.558	2423.5583	$X_{1/2}$	$X_{3/2}$	9	8	17.5	17.5
2424.248	2424.2536	$X_{3/2}$	$X_{1/2}$	6	5	20.5	19.5
2431.692	2431.6935	$X_{3/2}$	$X_{3/2}$	2	1	22.5	21.5
2433.166	2433.1644	$X_{3/2}$	$X_{1/2}$	3	2	11.5	10.5
2433.664	2433.6618	$X_{3/2}$	$X_{3/2}$	2	1	22.5	21.5
2433.870	2433.8638	$X_{1/2}$	$X_{1/2}$	7	6	13.5	13.5
2434.759	2434.7554	$X_{1/2}$	$X_{1/2}$	2	1	21.5	20.5
2435.154	2435.1558	$X_{3/2}$	$X_{1/2}$	1	0	22.5	22.5
2452.333	2452.3357	$X_{1/2}$	$X_{3/2}$	8	7	7.5	6.5
2453.348	2453.3513	$X_{3/2}$	$X_{3/2}$	1	0	24.5	23.5
2454.287	2454.2851	$X_{3/2}$	$X_{1/2}$	6	5	6.5	6.5
2454.407	2454.4094	$X_{3/2}$	$X_{3/2}$	3	2	19.5	18.5
2454.602	2454.6040	$X_{3/2}$	$X_{1/2}$	7	6	1.5	0.5
2455.533	2455.5333	$X_{3/2}$	$X_{3/2}$	1	0	24.5	23.5
2456.074	2456.0785	$X_{3/2}$	$X_{3/2}$	3	2	19.5	18.5
2456.500	2456.4964	$X_{1/2}$	$X_{1/2}$	1	0	40.5	40.5
2457.190	2457.1880	$X_{3/2}$	$X_{3/2}$	7	6	5.5	4.5
2457.861	2457.8653	$X_{1/2}$	$X_{3/2}$	9	8	12.5	12.5
2460.001	2459.9981	$X_{1/2}$	$X_{1/2}$	7	6	12.5	12.5
2462.496	2462.4913	$X_{3/2}$	$X_{3/2}$	1	0	41.5	41.5
2470.981	2470.9841	$X_{3/2}$	$X_{1/2}$	2	1	36.5	35.5

Table S1 (continued)

ν_{exp} (cm^{-1})	ν_{the} (cm^{-1})	GUQ	GLQ	v'	v''	J'	J''
2473.382	2473.3816	$X_{3/2}$	$X_{3/2}$	8	7	2.5	1.5
2479.688	2479.6912	$X_{3/2}$	$X_{1/2}$	13	11	9.5	8.5
2481.945	2481.9499	$X_{1/2}$	$X_{1/2}$	5	4	24.5	24.5
2485.635	2485.6363	$X_{3/2}$	$X_{3/2}$	2	1	21.5	20.5
2486.743	2486.7414	$X_{3/2}$	$X_{1/2}$	4	3	29.5	28.5
2487.226	2487.2307	$X_{1/2}$	$X_{1/2}$	2	1	20.5	19.5
2487.524	2487.5245	$X_{3/2}$	$X_{3/2}$	2	1	21.5	20.5
2488.738	2488.7348	$X_{1/2}$	$X_{1/2}$	2	1	20.5	19.5
2490.977	2490.9744	$X_{1/2}$	$X_{1/2}$	2	1	36.5	36.5
2500.841	2500.8355	$X_{3/2}$	$X_{3/2}$	5	4	12.5	11.5
2505.305	2505.3086	$X_{3/2}$	$X_{3/2}$	3	2	18.5	17.5
2505.456	2505.4605	$X_{1/2}$	$X_{3/2}$	5	4	26.5	25.5
2506.890	2506.8912	$X_{3/2}$	$X_{3/2}$	3	2	18.5	17.5
2508.656	2508.6584	$X_{1/2}$	$X_{1/2}$	3	2	17.5	16.5
2510.949	2510.9502	$X_{1/2}$	$X_{1/2}$	1	0	22.5	21.5
2511.732	2511.7334	$X_{3/2}$	$X_{3/2}$	1	0	23.5	22.5
2512.209	2512.2138	$X_{3/2}$	$X_{3/2}$	4	3	15.5	14.5
2512.663	2512.6651	$X_{1/2}$	$X_{1/2}$	1	0	22.5	21.5
2514.184	2514.1848	$X_{3/2}$	$X_{3/2}$	8	7	4.5	3.5
2514.840	2514.8380	$X_{1/2}$	$X_{1/2}$	4	3	14.5	13.5
2524.246	2524.2509	$X_{3/2}$	$X_{1/2}$	2	1	36.5	35.5
2532.667	2532.6680	$X_{3/2}$	$X_{3/2}$	8	7	5.5	4.5
2536.945	2536.9448	$X_{1/2}$	$X_{3/2}$	10	9	13.5	12.5
2537.138	2537.1362	$X_{3/2}$	$X_{1/2}$	6	5	3.5	3.5
2538.344	2538.3461	$X_{3/2}$	$X_{1/2}$	6	5	16.5	15.5
2539.080	2539.0806	$X_{3/2}$	$X_{3/2}$	2	1	20.5	19.5
2540.801	2540.8019	$X_{1/2}$	$X_{1/2}$	2	1	19.5	18.5
2540.882	2540.8862	$X_{3/2}$	$X_{3/2}$	2	1	20.5	19.5
2542.233	2542.2291	$X_{1/2}$	$X_{1/2}$	2	1	19.5	18.5
2545.740	2545.7442	$X_{3/2}$	$X_{1/2}$	5	4	5.5	4.5
2545.817	2545.8228	$X_{3/2}$	$X_{3/2}$	4	3	27.5	27.5
2546.874	2546.8766	$X_{3/2}$	$X_{3/2}$	6	5	17.5	17.5
2550.359	2550.3599	$X_{1/2}$	$X_{1/2}$	3	2	31.5	31.5
2551.110	2551.1114	$X_{1/2}$	$X_{3/2}$	7	6	11.5	10.5
2552.471	2552.4720	$X_{1/2}$	$X_{1/2}$	13	11	11.5	10.5
2555.564	2555.5650	$X_{3/2}$	$X_{3/2}$	3	2	17.5	16.5
2556.656	2556.6539	$X_{3/2}$	$X_{3/2}$	7	6	6.5	6.5
2557.892	2557.8937	$X_{1/2}$	$X_{1/2}$	3	2	16.5	15.5
2558.811	2558.8115	$X_{3/2}$	$X_{3/2}$	4	3	14.5	13.5
2559.035	2559.0365	$X_{1/2}$	$X_{1/2}$	3	2	16.5	15.5
2560.933	2560.9321	$X_{1/2}$	$X_{1/2}$	4	3	13.5	12.5
2563.325	2563.3282	$X_{3/2}$	$X_{3/2}$	7	6	5.5	5.5
2565.504	2565.5049	$X_{3/2}$	$X_{3/2}$	1	0	22.5	21.5

Table S1 (continued)

ν_{exp} (cm^{-1})	ν_{the} (cm^{-1})	GUQ	GLQ	v'	v''	J'	J''
2566.918	2566.9233	$X_{1/2}$	$X_{1/2}$	1	0	21.5	20.5
2567.528	2567.5317	$X_{3/2}$	$X_{1/2}$	6	5	14.5	13.5
2568.462	2568.4643	$X_{1/2}$	$X_{3/2}$	4	3	28.5	27.5
2568.561	2568.5635	$X_{1/2}$	$X_{1/2}$	1	0	21.5	20.5
2569.119	2569.1241	$X_{3/2}$	$X_{1/2}$	4	3	11.5	11.5
2576.564	2576.5599	$X_{3/2}$	$X_{1/2}$	5	4	7.5	7.5
2578.544	2578.5451	$X_{1/2}$	$X_{3/2}$	1	0	40.5	39.5
2579.098	2579.0949	$X_{3/2}$	$X_{3/2}$	8	7	8.5	7.5
2581.389	2581.3837	$X_{3/2}$	$X_{3/2}$	7	6	2.5	2.5
2582.162	2582.1653	$X_{1/2}$	$X_{1/2}$	7	6	1.5	1.5
2584.366	2584.3708	$X_{3/2}$	$X_{3/2}$	7	6	1.5	1.5
2585.191	2585.1945	$X_{3/2}$	$X_{3/2}$	5	4	10.5	9.5
2587.241	2587.2431	$X_{1/2}$	$X_{3/2}$	6	5	17.5	16.5
2591.877	2591.8805	$X_{1/2}$	$X_{3/2}$	8	7	2.5	2.5
2591.986	2591.9852	$X_{3/2}$	$X_{3/2}$	2	1	19.5	18.5
2593.706	2593.7059	$X_{3/2}$	$X_{3/2}$	2	1	19.5	18.5
2593.848	2593.8526	$X_{1/2}$	$X_{1/2}$	2	1	18.5	17.5
2595.205	2595.2009	$X_{1/2}$	$X_{1/2}$	2	1	18.5	17.5
2599.016	2599.0163	$X_{1/2}$	$X_{1/2}$	8	7	9.5	8.5
2602.024	2602.0221	$X_{3/2}$	$X_{3/2}$	8	7	19.5	18.5
2603.687	2603.6900	$X_{1/2}$	$X_{3/2}$	5	4	23.5	22.5
2604.566	2604.5657	$X_{3/2}$	$X_{3/2}$	4	3	13.5	12.5
2605.132	2605.1351	$X_{3/2}$	$X_{3/2}$	3	2	16.5	15.5
2606.535	2606.5399	$X_{3/2}$	$X_{3/2}$	3	2	16.5	15.5
2607.700	2607.7009	$X_{1/2}$	$X_{1/2}$	3	2	15.5	14.5
2608.757	2608.7622	$X_{1/2}$	$X_{1/2}$	3	2	15.5	14.5
2609.785	2609.7795	$X_{3/2}$	$X_{1/2}$	13	11	8.5	8.5
2611.977	2611.9805	$X_{1/2}$	$X_{3/2}$	3	2	31.5	30.5
2614.565	2614.5637	$X_{3/2}$	$X_{3/2}$	1	0	38.5	38.5
2616.422	2616.4201	$X_{3/2}$	$X_{3/2}$	8	7	12.5	11.5
2617.803	2617.8090	$X_{3/2}$	$X_{3/2}$	8	7	17.5	16.5
2619.893	2619.8889	$X_{3/2}$	$X_{3/2}$	6	5	13.5	13.5
2620.942	2620.9427	$X_{3/2}$	$X_{3/2}$	1	0	21.5	20.5
2621.369	2621.3710	$X_{1/2}$	$X_{1/2}$	8	7	15.5	14.5
2621.876	2621.8732	$X_{3/2}$	$X_{3/2}$	8	7	16.5	15.5
2622.469	2622.4699	$X_{1/2}$	$X_{1/2}$	1	0	20.5	19.5
2622.888	2622.8886	$X_{3/2}$	$X_{3/2}$	1	0	21.5	20.5
2623.087	2623.0915	$X_{3/2}$	$X_{1/2}$	3	2	13.5	13.5
2623.807	2623.8074	$X_{3/2}$	$X_{3/2}$	8	7	15.5	14.5
2624.032	2624.0331	$X_{1/2}$	$X_{1/2}$	1	0	20.5	19.5
2630.277	2630.2726	$X_{3/2}$	$X_{3/2}$	6	5	13.5	13.5
2631.752	2631.7467	$X_{1/2}$	$X_{1/2}$	6	5	4.5	3.5
2632.517	2632.5202	$X_{1/2}$	$X_{1/2}$	5	4	8.5	7.5

Table S1 (continued)

ν_{exp} (cm^{-1})	ν_{the} (cm^{-1})	GUQ	GLQ	v'	v''	J'	J''
2642.780	2642.7822	$X_{1/2}$	$X_{3/2}$	8	7	17.5	17.5
2642.901	2642.8993	$X_{1/2}$	$X_{3/2}$	8	7	8.5	8.5
2644.306	2644.3074	$X_{3/2}$	$X_{3/2}$	2	1	18.5	17.5
2645.939	2645.9410	$X_{3/2}$	$X_{3/2}$	2	1	18.5	17.5
2646.343	2646.3441	$X_{1/2}$	$X_{1/2}$	2	1	17.5	16.5
2647.616	2647.6113	$X_{1/2}$	$X_{1/2}$	2	1	17.5	16.5
2647.732	2647.7335	$X_{1/2}$	$X_{1/2}$	5	4	18.5	18.5
2648.429	2648.4323	$X_{3/2}$	$X_{3/2}$	4	3	12.5	11.5
2649.430	2649.4261	$X_{3/2}$	$X_{3/2}$	4	3	12.5	11.5
2650.388	2650.3821	$X_{3/2}$	$X_{3/2}$	6	5	4.5	3.5
2653.976	2653.9735	$X_{3/2}$	$X_{3/2}$	3	2	15.5	14.5
2654.691	2654.6934	$X_{1/2}$	$X_{3/2}$	8	7	10.5	10.5
2655.290	2655.2874	$X_{3/2}$	$X_{3/2}$	3	2	15.5	14.5
2655.589	2655.5834	$X_{1/2}$	$X_{1/2}$	7	6	2.5	1.5
2656.821	2656.8205	$X_{1/2}$	$X_{1/2}$	3	2	14.5	13.5
2657.797	2657.7987	$X_{1/2}$	$X_{1/2}$	3	2	14.5	13.5
2659.354	2659.3532	$X_{3/2}$	$X_{1/2}$	6	5	5.5	4.5
2665.229	2665.2234	$X_{3/2}$	$X_{3/2}$	5	4	8.5	7.5
2665.334	2665.3318	$X_{1/2}$	$X_{3/2}$	1	0	37.5	36.5
2665.812	2665.8061	$X_{3/2}$	$X_{3/2}$	5	4	8.5	7.5
2675.901	2675.8986	$X_{3/2}$	$X_{3/2}$	1	0	20.5	19.5
2677.551	2677.5501	$X_{1/2}$	$X_{1/2}$	1	0	19.5	18.5
2677.756	2677.7607	$X_{3/2}$	$X_{3/2}$	1	0	20.5	19.5
2679.031	2679.0341	$X_{1/2}$	$X_{1/2}$	1	0	19.5	18.5
2686.409	2686.4040	$X_{3/2}$	$X_{3/2}$	6	5	9.5	9.5
2688.175	2688.1759	$X_{1/2}$	$X_{3/2}$	8	7	3.5	2.5
2690.776	2690.7744	$X_{3/2}$	$X_{1/2}$	4	3	8.5	8.5
2691.102	2691.0958	$X_{3/2}$	$X_{3/2}$	7	6	4.5	3.5
2693.337	2693.3388	$X_{3/2}$	$X_{3/2}$	4	3	11.5	10.5
2696.001	2696.0026	$X_{3/2}$	$X_{3/2}$	2	1	17.5	16.5
2696.304	2696.3045	$X_{3/2}$	$X_{1/2}$	5	4	3.5	3.5
2697.274	2697.2765	$X_{1/2}$	$X_{1/2}$	4	3	10.5	9.5
2697.543	2697.5472	$X_{3/2}$	$X_{3/2}$	2	1	17.5	16.5
2698.236	2698.2371	$X_{1/2}$	$X_{1/2}$	2	1	16.5	15.5
2698.346	2698.3498	$X_{1/2}$	$X_{3/2}$	6	5	12.5	11.5
2699.425	2699.4212	$X_{1/2}$	$X_{1/2}$	2	1	16.5	15.5
2702.038	2702.0327	$X_{3/2}$	$X_{3/2}$	3	2	14.5	13.5
2702.877	2702.8766	$X_{1/2}$	$X_{1/2}$	7	6	4.5	3.5
2703.257	2703.2545	$X_{3/2}$	$X_{3/2}$	3	2	14.5	13.5
2703.437	2703.4349	$X_{3/2}$	$X_{3/2}$	5	4	7.5	6.5
2703.921	2703.9222	$X_{3/2}$	$X_{3/2}$	5	4	7.5	6.5
2703.999	2703.9937	$X_{3/2}$	$X_{1/2}$	1	0	11.5	10.5
2705.215	2705.2161	$X_{1/2}$	$X_{1/2}$	3	2	13.5	12.5

Table S1 (continued)

ν_{exp} (cm^{-1})	ν_{the} (cm^{-1})	GUQ	GLQ	v'	v''	J'	J''
2706.106	2706.1100	$X_{1/2}$	$X_{1/2}$	3	2	13.5	12.5
2711.024	2711.0281	$X_{3/2}$	$X_{3/2}$	6	5	7.5	7.5
2711.534	2711.5377	$X_{1/2}$	$X_{3/2}$	4	3	24.5	23.5
2714.011	2714.0154	$X_{3/2}$	$X_{3/2}$	5	4	17.5	17.5
2714.190	2714.1866	$X_{1/2}$	$X_{1/2}$	4	3	22.5	22.5
2722.482	2722.4793	$X_{1/2}$	$X_{3/2}$	8	7	4.5	3.5
2723.361	2723.3661	$X_{1/2}$	$X_{1/2}$	7	6	5.5	4.5
2723.558	2723.5546	$X_{3/2}$	$X_{3/2}$	6	5	6.5	6.5
2727.582	2727.5860	$X_{3/2}$	$X_{1/2}$	3	2	27.5	26.5
2730.332	2730.3288	$X_{3/2}$	$X_{3/2}$	1	0	19.5	18.5
2733.523	2733.5259	$X_{1/2}$	$X_{1/2}$	1	0	18.5	17.5
2740.326	2740.3293	$X_{3/2}$	$X_{3/2}$	5	4	6.5	5.5
2740.717	2740.7199	$X_{3/2}$	$X_{3/2}$	5	4	6.5	5.5
2741.057	2741.0520	$X_{1/2}$	$X_{1/2}$	4	3	9.5	8.5
2741.567	2741.5627	$X_{1/2}$	$X_{1/2}$	4	3	9.5	8.5
2746.692	2746.6946	$X_{1/2}$	$X_{1/2}$	5	4	14.5	14.5
2747.019	2747.0247	$X_{3/2}$	$X_{3/2}$	2	1	16.5	15.5
2747.423	2747.4238	$X_{3/2}$	$X_{3/2}$	6	5	2.5	2.5
2748.475	2748.4785	$X_{3/2}$	$X_{3/2}$	2	1	16.5	15.5
2749.267	2749.2629	$X_{3/2}$	$X_{3/2}$	3	2	13.5	12.5
2749.388	2749.3841	$X_{3/2}$	$X_{1/2}$	13	11	4.5	3.5
2749.494	2749.4918	$X_{1/2}$	$X_{1/2}$	2	1	15.5	14.5
2750.393	2750.3913	$X_{3/2}$	$X_{3/2}$	3	2	13.5	12.5
2750.592	2750.5912	$X_{1/2}$	$X_{1/2}$	2	1	15.5	14.5
2750.703	2750.7009	$X_{1/2}$	$X_{1/2}$	3	2	25.5	25.5
2751.908	2751.9069	$X_{1/2}$	$X_{1/2}$	5	4	5.5	4.5
2752.855	2752.8527	$X_{1/2}$	$X_{1/2}$	3	2	12.5	11.5
2753.661	2753.6611	$X_{1/2}$	$X_{1/2}$	3	2	12.5	11.5
2754.870	2754.8674	$X_{3/2}$	$X_{3/2}$	1	0	35.5	35.5
2758.355	2758.3594	$X_{3/2}$	$X_{1/2}$	3	2	10.5	10.5
2758.610	2758.6150	$X_{1/2}$	$X_{3/2}$	7	6	1.5	1.5
2764.266	2764.2669	$X_{3/2}$	$X_{3/2}$	13	11	10.5	10.5
2765.008	2765.0129	$X_{3/2}$	$X_{3/2}$	7	6	23.5	22.5
2767.952	2767.9491	$X_{1/2}$	$X_{1/2}$	13	11	9.5	9.5
2769.375	2769.3715	$X_{3/2}$	$X_{1/2}$	1	0	33.5	32.5
2775.803	2775.8081	$X_{3/2}$	$X_{3/2}$	5	4	5.5	4.5
2776.106	2776.1014	$X_{3/2}$	$X_{3/2}$	5	4	5.5	4.5
2776.291	2776.2959	$X_{3/2}$	$X_{1/2}$	13	11	4.5	4.5
2778.075	2778.0804	$X_{3/2}$	$X_{3/2}$	4	3	9.5	8.5
2778.970	2778.9744	$X_{3/2}$	$X_{3/2}$	4	3	20.5	20.5
2783.957	2783.9606	$X_{1/2}$	$X_{1/2}$	4	3	8.5	7.5
2784.187	2784.1878	$X_{3/2}$	$X_{3/2}$	1	0	18.5	17.5
2784.381	2784.3826	$X_{1/2}$	$X_{1/2}$	4	3	8.5	7.5

Table S1 (continued)

ν_{exp} (cm^{-1})	ν_{the} (cm^{-1})	GUQ	GLQ	v'	v''	J'	J''
2785.212	2785.2163	$X_{3/2}$	$X_{1/2}$	3	2	6.5	5.5
2785.876	2785.8752	$X_{3/2}$	$X_{3/2}$	1	0	18.5	17.5
2786.149	2786.1478	$X_{1/2}$	$X_{1/2}$	1	0	17.5	16.5
2787.466	2787.4671	$X_{1/2}$	$X_{1/2}$	1	0	17.5	16.5
2792.669	2792.6658	$X_{1/2}$	$X_{3/2}$	8	7	6.5	5.5
2793.320	2793.3209	$X_{1/2}$	$X_{1/2}$	7	6	20.5	19.5
2795.613	2795.6111	$X_{3/2}$	$X_{3/2}$	3	2	12.5	11.5
2796.232	2796.2372	$X_{1/2}$	$X_{3/2}$	9	8	14.5	13.5
2796.647	2796.6451	$X_{3/2}$	$X_{3/2}$	3	2	12.5	11.5
2797.321	2797.3258	$X_{3/2}$	$X_{3/2}$	2	1	15.5	14.5
2798.685	2798.6871	$X_{3/2}$	$X_{3/2}$	2	1	15.5	14.5
2799.081	2799.0789	$X_{1/2}$	$X_{3/2}$	5	4	15.5	14.5
2799.699	2799.6977	$X_{1/2}$	$X_{1/2}$	3	2	11.5	10.5
2800.064	2800.0688	$X_{1/2}$	$X_{1/2}$	2	1	14.5	13.5
2800.420	2800.4192	$X_{1/2}$	$X_{1/2}$	3	2	11.5	10.5
2801.083	2801.0820	$X_{1/2}$	$X_{1/2}$	2	1	14.5	13.5
2803.712	2803.7056	$X_{3/2}$	$X_{3/2}$	7	6	20.5	19.5
2804.316	2804.3137	$X_{3/2}$	$X_{1/2}$	13	11	3.5	3.5
2806.840	2806.8432	$X_{1/2}$	$X_{1/2}$	7	6	11.5	10.5
2807.115	2807.1153	$X_{1/2}$	$X_{3/2}$	7	6	6.5	6.5
2809.282	2809.2880	$X_{3/2}$	$X_{1/2}$	4	3	18.5	17.5
2809.455	2809.4578	$X_{1/2}$	$X_{3/2}$	5	4	15.5	14.5
2811.884	2811.8842	$X_{1/2}$	$X_{1/2}$	7	6	18.5	17.5
2812.793	2812.7874	$X_{3/2}$	$X_{1/2}$	1	0	15.5	15.5
2814.074	2814.0775	$X_{1/2}$	$X_{1/2}$	3	1	46.5	45.5
2814.150	2814.1500	$X_{1/2}$	$X_{1/2}$	3	2	24.5	24.5
2814.809	2814.8130	$X_{3/2}$	$X_{3/2}$	7	6	13.5	12.5
2814.921	2814.9259	$X_{3/2}$	$X_{1/2}$	2	1	12.5	12.5
2816.198	2816.2029	$X_{3/2}$	$X_{3/2}$	13	11	7.5	6.5
2816.501	2816.5064	$X_{1/2}$	$X_{3/2}$	7	6	7.5	7.5
2818.158	2818.1570	$X_{3/2}$	$X_{3/2}$	4	3	8.5	7.5
2818.646	2818.6419	$X_{1/2}$	$X_{1/2}$	7	6	13.5	12.5
2818.771	2818.7715	$X_{3/2}$	$X_{3/2}$	4	3	8.5	7.5
2819.600	2819.6014	$X_{3/2}$	$X_{3/2}$	7	6	14.5	13.5
2820.314	2820.3107	$X_{1/2}$	$X_{3/2}$	6	5	5.5	4.5
2821.167	2821.1632	$X_{1/2}$	$X_{1/2}$	7	6	16.5	15.5
2825.995	2825.9971	$X_{1/2}$	$X_{1/2}$	4	3	7.5	6.5
2826.478	2826.4731	$X_{3/2}$	$X_{1/2}$	13	11	2.5	2.5
2827.096	2827.0986	$X_{1/2}$	$X_{1/2}$	5	4	3.5	2.5
2829.378	2829.3777	$X_{3/2}$	$X_{1/2}$	3	2	23.5	22.5
2837.428	2837.4287	$X_{3/2}$	$X_{3/2}$	1	0	17.5	16.5
2838.076	2838.0783	$X_{1/2}$	$X_{3/2}$	6	5	4.5	3.5
2839.024	2839.0254	$X_{3/2}$	$X_{3/2}$	1	0	17.5	16.5

Table S1 (continued)

ν_{exp} (cm^{-1})	ν_{the} (cm^{-1})	GUQ	GLQ	v'	v''	J'	J''
2839.585	2839.5819	$X_{1/2}$	$X_{1/2}$	1	0	16.5	15.5
2840.818	2840.8160	$X_{1/2}$	$X_{1/2}$	1	0	16.5	15.5
2841.023	2841.0210	$X_{3/2}$	$X_{3/2}$	3	2	11.5	10.5
2841.964	2841.9596	$X_{3/2}$	$X_{3/2}$	3	2	11.5	10.5
2842.227	2842.2214	$X_{3/2}$	$X_{3/2}$	1	0	33.5	33.5
2845.720	2845.7220	$X_{1/2}$	$X_{1/2}$	3	2	10.5	9.5
2846.851	2846.8558	$X_{3/2}$	$X_{3/2}$	2	1	14.5	13.5
2848.122	2848.1232	$X_{3/2}$	$X_{3/2}$	2	1	14.5	13.5
2849.931	2849.9294	$X_{1/2}$	$X_{1/2}$	2	1	13.5	12.5
2850.860	2850.8551	$X_{1/2}$	$X_{1/2}$	2	1	13.5	12.5
2851.638	2851.6384	$X_{1/2}$	$X_{3/2}$	7	6	12.5	12.5
2852.098	2852.1020	$X_{1/2}$	$X_{1/2}$	6	5	3.5	2.5
2857.721	2857.7188	$X_{3/2}$	$X_{3/2}$	4	3	7.5	6.5
2857.914	2857.9087	$X_{1/2}$	$X_{3/2}$	6	5	3.5	2.5
2858.232	2858.2351	$X_{3/2}$	$X_{3/2}$	4	3	7.5	6.5
2862.178	2862.1738	$X_{3/2}$	$X_{1/2}$	13	11	5.5	4.5
2864.373	2864.3776	$X_{3/2}$	$X_{1/2}$	2	0	47.5	47.5
2867.170	2867.1683	$X_{1/2}$	$X_{1/2}$	4	3	6.5	5.5
2867.404	2867.4078	$X_{1/2}$	$X_{1/2}$	4	3	6.5	5.5
2872.328	2872.3286	$X_{1/2}$	$X_{1/2}$	2	1	27.5	27.5
2881.084	2881.0817	$X_{3/2}$	$X_{1/2}$	3	2	7.5	7.5
2885.435	2885.4379	$X_{1/2}$	$X_{3/2}$	5	4	11.5	10.5
2886.275	2886.2734	$X_{3/2}$	$X_{3/2}$	3	2	10.5	9.5
2889.080	2889.0761	$X_{3/2}$	$X_{1/2}$	2	1	26.5	25.5
2890.003	2890.0029	$X_{3/2}$	$X_{3/2}$	1	0	16.5	15.5
2890.670	2890.6739	$X_{3/2}$	$X_{1/2}$	2	0	40.5	39.5
2890.905	2890.9021	$X_{1/2}$	$X_{1/2}$	3	2	9.5	8.5
2891.506	2891.5070	$X_{3/2}$	$X_{3/2}$	1	0	16.5	15.5
2892.381	2892.3835	$X_{1/2}$	$X_{1/2}$	1	0	15.5	14.5
2893.533	2893.5306	$X_{1/2}$	$X_{1/2}$	1	0	15.5	14.5
2895.560	2895.5626	$X_{3/2}$	$X_{3/2}$	2	1	13.5	12.5
2895.958	2895.9590	$X_{3/2}$	$X_{3/2}$	4	3	6.5	5.5
2896.064	2896.0686	$X_{1/2}$	$X_{1/2}$	5	4	4.5	4.5
2896.373	2896.3751	$X_{3/2}$	$X_{3/2}$	4	3	6.5	5.5
2896.733	2896.7346	$X_{3/2}$	$X_{3/2}$	2	1	13.5	12.5
2899.036	2899.0363	$X_{1/2}$	$X_{1/2}$	2	1	12.5	11.5
2899.878	2899.8732	$X_{1/2}$	$X_{1/2}$	2	1	12.5	11.5
2901.970	2901.9660	$X_{3/2}$	$X_{3/2}$	5	4	4.5	4.5
2904.605	2904.6106	$X_{1/2}$	$X_{3/2}$	4	3	18.5	17.5
2907.488	2907.4925	$X_{1/2}$	$X_{1/2}$	4	3	5.5	4.5
2907.641	2907.6385	$X_{1/2}$	$X_{1/2}$	4	3	5.5	4.5
2914.691	2914.6862	$X_{1/2}$	$X_{3/2}$	13	11	9.5	8.5
2923.471	2923.4663	$X_{1/2}$	$X_{3/2}$	8	7	10.5	9.5

Table S1 (continued)

ν_{exp} (cm^{-1})	ν_{the} (cm^{-1})	GUQ	GLQ	v'	v''	J'	J''
2928.156	2928.1513	$X_{1/2}$	$X_{3/2}$	4	3	17.5	16.5
2928.773	2928.7749	$X_{3/2}$	$X_{3/2}$	3	2	9.5	8.5
2929.521	2929.5190	$X_{3/2}$	$X_{3/2}$	3	2	9.5	8.5
2932.769	2932.7695	$X_{3/2}$	$X_{3/2}$	4	3	5.5	4.5
2933.082	2933.0840	$X_{3/2}$	$X_{3/2}$	4	3	5.5	4.5
2935.220	2935.2225	$X_{1/2}$	$X_{1/2}$	3	2	8.5	7.5
2935.669	2935.6747	$X_{1/2}$	$X_{1/2}$	3	2	8.5	7.5
2936.420	2936.4180	$X_{3/2}$	$X_{1/2}$	1	0	29.5	28.5
2936.521	2936.5260	$X_{1/2}$	$X_{3/2}$	3	2	22.5	21.5
2938.785	2938.7820	$X_{3/2}$	$X_{1/2}$	1	0	8.5	7.5
2939.825	2939.8249	$X_{1/2}$	$X_{3/2}$	1	0	32.5	31.5
2941.861	2941.8603	$X_{3/2}$	$X_{3/2}$	1	0	15.5	14.5
2942.722	2942.7242	$X_{1/2}$	$X_{3/2}$	7	6	5.5	4.5
2943.271	2943.2700	$X_{3/2}$	$X_{3/2}$	1	0	15.5	14.5
2943.390	2943.3909	$X_{3/2}$	$X_{3/2}$	2	1	12.5	11.5
2945.334	2945.3301	$X_{1/2}$	$X_{3/2}$	6	5	3.5	3.5
2945.571	2945.5694	$X_{1/2}$	$X_{1/2}$	1	0	14.5	13.5
2946.855	2946.8547	$X_{1/2}$	$X_{3/2}$	2	1	46.5	46.5
2947.355	2947.3548	$X_{1/2}$	$X_{1/2}$	2	1	11.5	10.5
2948.105	2948.1017	$X_{1/2}$	$X_{1/2}$	2	1	11.5	10.5
2956.908	2956.9075	$X_{3/2}$	$X_{3/2}$	13	11	3.5	2.5
2958.895	2958.8905	$X_{1/2}$	$X_{1/2}$	1	0	29.5	29.5
2960.715	2960.7161	$X_{3/2}$	$X_{3/2}$	6	5	24.5	23.5
2961.030	2961.0325	$X_{1/2}$	$X_{1/2}$	6	5	23.5	22.5
2963.479	2963.4750	$X_{3/2}$	$X_{1/2}$	1	0	12.5	12.5
2963.672	2963.6684	$X_{1/2}$	$X_{1/2}$	5	4	1.5	0.5
2964.226	2964.2254	$X_{3/2}$	$X_{3/2}$	1	0	30.5	30.5
2964.686	2964.6890	$X_{1/2}$	$X_{3/2}$	6	5	24.5	24.5
2965.529	2965.5266	$X_{3/2}$	$X_{3/2}$	4	3	12.5	12.5
2966.544	2966.5493	$X_{1/2}$	$X_{3/2}$	3	2	21.5	20.5
2967.036	2967.0400	$X_{1/2}$	$X_{1/2}$	13	11	9.5	8.5
2968.038	2968.0379	$X_{3/2}$	$X_{3/2}$	4	3	4.5	3.5
2968.257	2968.2514	$X_{3/2}$	$X_{3/2}$	4	3	4.5	3.5
2968.427	2968.4297	$X_{1/2}$	$X_{3/2}$	6	5	24.5	24.5
2970.976	2970.9753	$X_{3/2}$	$X_{3/2}$	3	2	8.5	7.5
2971.620	2971.6201	$X_{3/2}$	$X_{3/2}$	3	2	8.5	7.5
2975.969	2975.9640	$X_{3/2}$	$X_{3/2}$	4	3	12.5	12.5
2978.682	2978.6790	$X_{1/2}$	$X_{1/2}$	3	2	7.5	6.5
2978.821	2978.8232	$X_{3/2}$	$X_{1/2}$	4	3	3.5	2.5
2979.040	2979.0378	$X_{1/2}$	$X_{1/2}$	3	2	7.5	6.5
2979.632	2979.6280	$X_{1/2}$	$X_{3/2}$	1	0	31.5	30.5
2980.388	2980.3903	$X_{1/2}$	$X_{3/2}$	7	6	6.5	5.5
2981.240	2981.2385	$X_{1/2}$	$X_{1/2}$	6	5	10.5	9.5

Table S1 (continued)

ν_{exp} (cm^{-1})	ν_{the} (cm^{-1})	GUQ	GLQ	v'	v''	J'	J''
2982.930	2982.9356	$X_{1/2}$	$X_{1/2}$	13	11	2.5	2.5
2983.165	2983.1633	$X_{1/2}$	$X_{1/2}$	13	11	2.5	2.5
2987.763	2987.7583	$X_{1/2}$	$X_{3/2}$	13	11	10.5	10.5
2988.189	2988.1932	$X_{3/2}$	$X_{3/2}$	6	5	22.5	21.5
2990.281	2990.2823	$X_{3/2}$	$X_{3/2}$	2	1	11.5	10.5
2990.976	2990.9731	$X_{3/2}$	$X_{3/2}$	1	0	28.5	28.5
2991.259	2991.2599	$X_{3/2}$	$X_{3/2}$	2	1	11.5	10.5
2992.949	2992.9487	$X_{3/2}$	$X_{3/2}$	1	0	14.5	13.5
2994.265	2994.2623	$X_{3/2}$	$X_{3/2}$	1	0	14.5	13.5
2994.851	2994.8542	$X_{1/2}$	$X_{1/2}$	2	1	10.5	9.5
2995.514	2995.5099	$X_{1/2}$	$X_{1/2}$	2	1	10.5	9.5
2995.751	2995.7517	$X_{3/2}$	$X_{1/2}$	3	2	17.5	16.5
2995.922	2995.9231	$X_{1/2}$	$X_{1/2}$	1	0	13.5	12.5
2996.896	2996.8917	$X_{1/2}$	$X_{1/2}$	1	0	13.5	12.5
3001.790	3001.7913	$X_{3/2}$	$X_{3/2}$	4	3	3.5	2.5
3011.946	3011.9450	$X_{3/2}$	$X_{3/2}$	3	2	7.5	6.5
3012.858	3012.8597	$X_{1/2}$	$X_{1/2}$	6	5	14.5	13.5
3013.566	3013.5665	$X_{3/2}$	$X_{3/2}$	6	5	15.5	14.5
3015.970	3015.9746	$X_{1/2}$	$X_{3/2}$	6	5	9.5	9.5
3016.857	3016.8604	$X_{3/2}$	$X_{3/2}$	6	5	17.5	16.5
3016.990	3016.9867	$X_{3/2}$	$X_{3/2}$	6	5	17.5	16.5
3018.333	3018.3303	$X_{1/2}$	$X_{3/2}$	8	7	14.5	13.5
3021.287	3021.2929	$X_{1/2}$	$X_{1/2}$	5	4	3.5	2.5
3021.543	3021.5446	$X_{1/2}$	$X_{1/2}$	3	2	6.5	5.5
3025.773	3025.7764	$X_{3/2}$	$X_{3/2}$	4	3	8.5	8.5
3032.281	3032.2812	$X_{3/2}$	$X_{3/2}$	5	4	4.5	3.5
3032.502	3032.4965	$X_{3/2}$	$X_{3/2}$	5	4	4.5	3.5
3036.173	3036.1732	$X_{3/2}$	$X_{3/2}$	2	1	10.5	9.5
3037.050	3037.0519	$X_{3/2}$	$X_{3/2}$	2	1	10.5	9.5
3041.509	3041.5096	$X_{1/2}$	$X_{1/2}$	2	1	9.5	8.5
3042.073	3042.0729	$X_{1/2}$	$X_{1/2}$	2	1	9.5	8.5
3043.217	3043.2137	$X_{3/2}$	$X_{3/2}$	1	0	13.5	12.5
3043.830	3043.8252	$X_{1/2}$	$X_{1/2}$	13	11	4.5	3.5
3044.431	3044.4298	$X_{3/2}$	$X_{3/2}$	1	0	13.5	12.5
3045.816	3045.8194	$X_{1/2}$	$X_{1/2}$	1	0	48.5	47.5
3046.582	3046.5811	$X_{1/2}$	$X_{1/2}$	1	0	12.5	11.5
3047.463	3047.4583	$X_{1/2}$	$X_{1/2}$	1	0	12.5	11.5
3047.833	3047.8312	$X_{1/2}$	$X_{3/2}$	6	5	16.5	16.5
3050.889	3050.8871	$X_{1/2}$	$X_{3/2}$	1	0	28.5	27.5
3051.582	3051.5819	$X_{3/2}$	$X_{3/2}$	3	2	6.5	5.5
3052.018	3052.0222	$X_{3/2}$	$X_{3/2}$	3	2	6.5	5.5
3052.389	3052.3931	$X_{1/2}$	$X_{1/2}$	4	3	35.5	34.5
3053.134	3053.1352	$X_{3/2}$	$X_{1/2}$	3	2	15.5	14.5

Table S1 (continued)

ν_{exp} (cm^{-1})	ν_{the} (cm^{-1})	GUQ	GLQ	v'	v''	J'	J''
3056.780	3056.7789	$X_{3/2}$	$X_{1/2}$	3	1	43.5	43.5
3058.015	3058.0117	$X_{3/2}$	$X_{3/2}$	4	3	5.5	5.5
3063.055	3063.0562	$X_{1/2}$	$X_{1/2}$	3	2	5.5	4.5
3063.221	3063.2208	$X_{1/2}$	$X_{1/2}$	3	2	5.5	4.5
3067.016	3067.0196	$X_{1/2}$	$X_{3/2}$	3	2	40.5	40.5
3069.504	3069.5043	$X_{1/2}$	$X_{1/2}$	3	1	44.5	43.5
3071.691	3071.6903	$X_{1/2}$	$X_{1/2}$	1	0	25.5	25.5
3073.515	3073.5207	$X_{3/2}$	$X_{3/2}$	4	3	2.5	2.5
3074.559	3074.5613	$X_{1/2}$	$X_{1/2}$	4	3	1.5	1.5
3076.574	3076.5764	$X_{3/2}$	$X_{3/2}$	4	3	1.5	1.5
3080.416	3080.4114	$X_{3/2}$	$X_{1/2}$	2	1	20.5	19.5
3080.993	3080.9937	$X_{3/2}$	$X_{3/2}$	2	1	9.5	8.5
3081.771	3081.7720	$X_{3/2}$	$X_{3/2}$	2	1	9.5	8.5
3087.306	3087.3054	$X_{1/2}$	$X_{1/2}$	2	1	8.5	7.5
3087.775	3087.7746	$X_{1/2}$	$X_{1/2}$	2	1	8.5	7.5
3089.767	3089.7681	$X_{3/2}$	$X_{3/2}$	3	2	5.5	4.5
3090.102	3090.1031	$X_{3/2}$	$X_{3/2}$	3	2	5.5	4.5
3092.600	3092.5985	$X_{3/2}$	$X_{3/2}$	1	0	12.5	11.5
3093.712	3093.7155	$X_{3/2}$	$X_{3/2}$	1	0	12.5	11.5
3093.985	3093.9803	$X_{1/2}$	$X_{3/2}$	2	1	23.5	22.5
3096.446	3096.4481	$X_{1/2}$	$X_{1/2}$	1	0	11.5	10.5
3097.233	3097.2327	$X_{1/2}$	$X_{1/2}$	1	0	11.5	10.5
3098.740	3098.7366	$X_{1/2}$	$X_{3/2}$	3	2	16.5	15.5
3104.038	3104.0349	$X_{1/2}$	$X_{1/2}$	3	2	4.5	3.5
3104.106	3104.1010	$X_{1/2}$	$X_{1/2}$	3	2	4.5	3.5
3120.086	3120.0920	$X_{1/2}$	$X_{1/2}$	2	1	43.5	42.5
3121.589	3121.5841	$X_{3/2}$	$X_{3/2}$	3	2	13.5	13.5
3124.666	3124.6647	$X_{3/2}$	$X_{3/2}$	2	1	8.5	7.5
3125.340	3125.3410	$X_{3/2}$	$X_{3/2}$	2	1	8.5	7.5
3126.376	3126.3772	$X_{3/2}$	$X_{3/2}$	3	2	4.5	3.5
3126.604	3126.6064	$X_{3/2}$	$X_{3/2}$	3	2	4.5	3.5
3127.411	3127.4094	$X_{3/2}$	$X_{1/2}$	3	2	10.5	9.5
3132.238	3132.2381	$X_{1/2}$	$X_{1/2}$	2	1	7.5	6.5
3132.615	3132.6111	$X_{1/2}$	$X_{1/2}$	2	1	7.5	6.5
3133.528	3133.5257	$X_{3/2}$	$X_{1/2}$	2	1	18.5	17.5
3136.941	3136.9426	$X_{1/2}$	$X_{1/2}$	3	2	11.5	11.5
3138.866	3138.8648	$X_{1/2}$	$X_{3/2}$	2	1	43.5	43.5
3141.044	3141.0422	$X_{3/2}$	$X_{3/2}$	1	0	11.5	10.5
3142.060	3142.0589	$X_{3/2}$	$X_{3/2}$	1	0	11.5	10.5
3144.651	3144.6488	$X_{3/2}$	$X_{3/2}$	3	2	11.5	11.5
3145.495	3145.4920	$X_{1/2}$	$X_{1/2}$	1	0	10.5	9.5
3146.184	3146.1825	$X_{1/2}$	$X_{1/2}$	1	0	10.5	9.5
3147.785	3147.7831	$X_{3/2}$	$X_{1/2}$	3	2	7.5	6.5

Table S1 (continued)

ν_{exp} (cm^{-1})	ν_{the} (cm^{-1})	GUQ	GLQ	v'	v''	J'	J''
3148.611	3148.6093	$X_{3/2}$	$X_{3/2}$	4	3	2.5	1.5
3151.843	3151.8400	$X_{3/2}$	$X_{1/2}$	1	0	23.5	22.5
3160.261	3160.2643	$X_{1/2}$	$X_{3/2}$	3	2	38.5	38.5
3161.425	3161.4285	$X_{3/2}$	$X_{3/2}$	3	2	3.5	2.5
3163.809	3163.8054	$X_{1/2}$	$X_{3/2}$	6	5	6.5	5.5
3164.742	3164.7432	$X_{1/2}$	$X_{3/2}$	13	11	11.5	10.5
3165.658	3165.6590	$X_{3/2}$	$X_{3/2}$	5	4	24.5	23.5
3167.096	3167.0945	$X_{3/2}$	$X_{3/2}$	2	1	7.5	6.5
3167.666	3167.6666	$X_{3/2}$	$X_{3/2}$	2	1	7.5	6.5
3175.415	3175.4138	$X_{1/2}$	$X_{1/2}$	1	0	46.5	45.5
3176.318	3176.3212	$X_{1/2}$	$X_{1/2}$	2	1	6.5	5.5
3176.596	3176.5956	$X_{1/2}$	$X_{1/2}$	2	1	6.5	5.5
3177.621	3177.6205	$X_{3/2}$	$X_{1/2}$	3	1	42.5	42.5
3183.472	3183.4730	$X_{1/2}$	$X_{1/2}$	3	2	2.5	1.5
3183.843	3183.8477	$X_{1/2}$	$X_{3/2}$	3	2	12.5	11.5
3188.097	3188.0997	$X_{1/2}$	$X_{1/2}$	5	4	21.5	20.5
3188.481	3188.4794	$X_{3/2}$	$X_{3/2}$	1	0	10.5	9.5
3189.393	3189.3945	$X_{3/2}$	$X_{3/2}$	1	0	10.5	9.5
3192.718	3192.7215	$X_{1/2}$	$X_{1/2}$	3	2	7.5	7.5
3193.401	3193.4037	$X_{3/2}$	$X_{3/2}$	5	4	14.5	13.5
3193.689	3193.6869	$X_{1/2}$	$X_{1/2}$	1	0	9.5	8.5
3194.284	3194.2819	$X_{1/2}$	$X_{1/2}$	1	0	9.5	8.5
3195.925	3195.9268	$X_{1/2}$	$X_{1/2}$	5	4	20.5	19.5
3196.032	3196.0310	$X_{3/2}$	$X_{3/2}$	4	3	32.5	31.5
3201.546	3201.5426	$X_{1/2}$	$X_{3/2}$	13	11	2.5	1.5
3201.631	3201.6361	$X_{1/2}$	$X_{1/2}$	5	4	19.5	18.5
3205.872	3205.8669	$X_{1/2}$	$X_{3/2}$	6	5	7.5	6.5
3206.354	3206.3478	$X_{1/2}$	$X_{1/2}$	5	4	16.5	15.5
3206.931	3206.9285	$X_{1/2}$	$X_{1/2}$	5	4	17.5	16.5
3208.176	3208.1747	$X_{3/2}$	$X_{3/2}$	2	1	6.5	5.5
3208.638	3208.6401	$X_{3/2}$	$X_{3/2}$	2	1	6.5	5.5
3210.348	3210.3485	$X_{1/2}$	$X_{1/2}$	2	1	15.5	15.5
3211.157	3211.1587	$X_{3/2}$	$X_{1/2}$	12	10	10.5	9.5
3213.243	3213.2383	$X_{3/2}$	$X_{1/2}$	1	0	22.5	21.5
3219.583	3219.5872	$X_{1/2}$	$X_{1/2}$	2	1	5.5	4.5
3219.764	3219.7602	$X_{1/2}$	$X_{1/2}$	2	1	5.5	4.5
3219.933	3219.9282	$X_{3/2}$	$X_{1/2}$	1	0	4.5	3.5
3220.432	3220.4334	$X_{1/2}$	$X_{3/2}$	3	2	10.5	9.5
3224.939	3224.9367	$X_{3/2}$	$X_{3/2}$	4	3	5.5	4.5
3225.943	3225.9419	$X_{1/2}$	$X_{3/2}$	5	4	19.5	19.5
3227.887	3227.8930	$X_{1/2}$	$X_{3/2}$	13	11	7.5	6.5
3228.062	3228.0589	$X_{1/2}$	$X_{1/2}$	3	2	3.5	3.5
3228.622	3228.6218	$X_{3/2}$	$X_{1/2}$	9	7	19.5	18.5

Table S1 (continued)

ν_{exp} (cm^{-1})	ν_{the} (cm^{-1})	GUQ	GLQ	v'	v''	J'	J''
3230.185	3230.1809	$X_{1/2}$	$X_{3/2}$	5	4	14.5	14.5
3231.647	3231.6464	$X_{3/2}$	$X_{3/2}$	3	2	3.5	3.5
3232.391	3232.3928	$X_{3/2}$	$X_{3/2}$	3	2	3.5	3.5
3233.606	3233.6099	$X_{1/2}$	$X_{1/2}$	3	2	2.5	2.5
3234.659	3234.6582	$X_{1/2}$	$X_{1/2}$	3	2	36.5	35.5
3234.839	3234.8379	$X_{3/2}$	$X_{3/2}$	1	0	9.5	8.5
3235.650	3235.6499	$X_{3/2}$	$X_{3/2}$	1	0	9.5	8.5
3238.196	3238.1923	$X_{1/2}$	$X_{1/2}$	1	0	20.5	20.5
3239.246	3239.2437	$X_{3/2}$	$X_{3/2}$	3	2	1.5	1.5
3239.837	3239.8362	$X_{1/2}$	$X_{1/2}$	1	0	45.5	44.5
3241.018	3241.0164	$X_{1/2}$	$X_{1/2}$	1	0	8.5	7.5
3241.516	3241.5146	$X_{1/2}$	$X_{1/2}$	4	3	5.5	4.5
3243.610	3243.6144	$X_{3/2}$	$X_{1/2}$	7	5	25.5	24.5
3244.210	3244.2065	$X_{1/2}$	$X_{1/2}$	2	1	14.5	14.5
3247.779	3247.7779	$X_{3/2}$	$X_{3/2}$	2	1	5.5	4.5
3248.134	3248.1339	$X_{3/2}$	$X_{3/2}$	2	1	5.5	4.5
3249.058	3249.0566	$X_{1/2}$	$X_{3/2}$	3	2	36.5	36.5
3251.413	3251.4092	$X_{3/2}$	$X_{1/2}$	2	1	13.5	12.5
3256.601	3256.5985	$X_{1/2}$	$X_{3/2}$	4	3	1.5	1.5
3257.779	3257.7808	$X_{1/2}$	$X_{3/2}$	1	0	22.5	21.5
3262.679	3262.6769	$X_{1/2}$	$X_{1/2}$	11	9	14.5	13.5
3263.003	3263.0051	$X_{3/2}$	$X_{3/2}$	1	0	20.5	20.5
3267.222	3267.2211	$X_{1/2}$	$X_{3/2}$	4	3	2.5	2.5
3267.530	3267.5273	$X_{3/2}$	$X_{1/2}$	1	0	5.5	5.5
3268.016	3268.0112	$X_{3/2}$	$X_{1/2}$	1	0	20.5	19.5
3269.344	3269.3466	$X_{3/2}$	$X_{3/2}$	4	3	7.5	6.5
3274.168	3274.1732	$X_{3/2}$	$X_{3/2}$	3	2	36.5	35.5
3274.286	3274.2898	$X_{1/2}$	$X_{3/2}$	2	1	16.5	15.5
3280.036	3280.0357	$X_{3/2}$	$X_{3/2}$	1	0	8.5	7.5
3280.742	3280.7429	$X_{3/2}$	$X_{3/2}$	1	0	8.5	7.5
3284.810	3284.8089	$X_{1/2}$	$X_{3/2}$	6	5	9.5	8.5
3285.763	3285.7631	$X_{3/2}$	$X_{3/2}$	2	1	4.5	3.5
3286.008	3286.0085	$X_{3/2}$	$X_{3/2}$	2	1	4.5	3.5
3287.479	3287.4782	$X_{1/2}$	$X_{1/2}$	1	0	7.5	6.5
3287.876	3287.8763	$X_{1/2}$	$X_{1/2}$	1	0	7.5	6.5
3294.768	3294.7639	$X_{1/2}$	$X_{3/2}$	4	3	28.5	28.5
3296.123	3296.1213	$X_{1/2}$	$X_{1/2}$	1	0	18.5	18.5
3299.463	3299.4577	$X_{1/2}$	$X_{1/2}$	4	3	27.5	26.5
3299.889	3299.8935	$X_{1/2}$	$X_{1/2}$	4	3	27.5	26.5
3307.468	3307.4682	$X_{1/2}$	$X_{3/2}$	1	0	44.5	44.5
3308.074	3308.0695	$X_{3/2}$	$X_{3/2}$	4	3	9.5	8.5
3309.004	3309.0014	$X_{1/2}$	$X_{3/2}$	4	3	5.5	5.5
3309.850	3309.8443	$X_{1/2}$	$X_{3/2}$	4	3	5.5	5.5

Table S1 (continued)

ν_{exp} (cm^{-1})	ν_{the} (cm^{-1})	GUQ	GLQ	v'	v''	J'	J''
3317.669	3317.6675	$X_{1/2}$	$X_{3/2}$	6	5	10.5	9.5
3318.916	3318.9191	$X_{3/2}$	$X_{3/2}$	1	0	19.5	19.5
3319.470	3319.4717	$X_{3/2}$	$X_{3/2}$	4	3	27.5	26.5
3320.103	3320.1079	$X_{3/2}$	$X_{3/2}$	12	10	6.5	5.5
3322.001	3322.0014	$X_{3/2}$	$X_{3/2}$	2	1	3.5	2.5
3322.145	3322.1409	$X_{3/2}$	$X_{3/2}$	2	1	3.5	2.5
3323.155	3323.1576	$X_{1/2}$	$X_{1/2}$	1	0	17.5	17.5
3323.979	3323.9772	$X_{3/2}$	$X_{3/2}$	1	0	7.5	6.5
3324.575	3324.5773	$X_{3/2}$	$X_{3/2}$	1	0	7.5	6.5
3326.493	3326.4958	$X_{1/2}$	$X_{1/2}$	3	2	2.5	1.5
3330.511	3330.5054	$X_{1/2}$	$X_{1/2}$	2	1	9.5	9.5
3333.089	3333.0887	$X_{1/2}$	$X_{1/2}$	1	0	6.5	5.5
3333.386	3333.3843	$X_{1/2}$	$X_{1/2}$	1	0	6.5	5.5
3336.241	3336.2431	$X_{3/2}$	$X_{3/2}$	4	3	26.5	25.5
3340.127	3340.1276	$X_{3/2}$	$X_{1/2}$	11	9	9.5	8.5
3340.845	3340.8413	$X_{1/2}$	$X_{3/2}$	1	0	20.5	19.5
3340.949	3340.9518	$X_{1/2}$	$X_{1/2}$	12	10	7.5	7.5
3341.757	3341.7607	$X_{3/2}$	$X_{1/2}$	12	10	1.5	0.5
3341.872	3341.8707	$X_{3/2}$	$X_{3/2}$	3	2	3.5	2.5
3344.674	3344.6773	$X_{1/2}$	$X_{1/2}$	2	1	2.5	1.5
3344.787	3344.7848	$X_{1/2}$	$X_{1/2}$	2	1	2.5	1.5
3346.239	3346.2351	$X_{1/2}$	$X_{1/2}$	3	2	33.5	32.5
3347.392	3347.3942	$X_{1/2}$	$X_{3/2}$	7	6	20.5	19.5
3351.085	3351.0795	$X_{3/2}$	$X_{3/2}$	2	1	8.5	8.5
3351.396	3351.3982	$X_{1/2}$	$X_{1/2}$	4	3	24.5	23.5
3351.517	3351.5183	$X_{1/2}$	$X_{1/2}$	4	3	24.5	23.5
3351.811	3351.8063	$X_{3/2}$	$X_{1/2}$	12	10	3.5	2.5
3355.203	3355.1983	$X_{1/2}$	$X_{3/2}$	4	3	25.5	25.5
3358.125	3358.1197	$X_{1/2}$	$X_{3/2}$	6	5	11.5	10.5
3360.166	3360.1598	$X_{3/2}$	$X_{1/2}$	1	0	2.5	2.5
3366.550	3366.5482	$X_{3/2}$	$X_{3/2}$	1	0	6.5	5.5
3367.037	3367.0383	$X_{3/2}$	$X_{3/2}$	1	0	6.5	5.5
3372.980	3372.9743	$X_{1/2}$	$X_{1/2}$	2	0	44.5	43.5
3374.117	3374.1137	$X_{1/2}$	$X_{1/2}$	4	3	13.5	12.5
3377.885	3377.8875	$X_{1/2}$	$X_{1/2}$	1	0	5.5	4.5
3378.075	3378.0773	$X_{1/2}$	$X_{1/2}$	1	0	5.5	4.5
3380.712	3380.7184	$X_{3/2}$	$X_{1/2}$	1	0	1.5	1.5
3383.422	3383.4217	$X_{3/2}$	$X_{3/2}$	4	3	15.5	14.5
3384.225	3384.2238	$X_{1/2}$	$X_{3/2}$	2	1	38.5	38.5
3388.768	3388.7677	$X_{1/2}$	$X_{1/2}$	4	3	15.5	14.5
3389.176	3389.1720	$X_{1/2}$	$X_{1/2}$	4	3	20.5	19.5
3391.334	3391.3333	$X_{1/2}$	$X_{3/2}$	4	3	11.5	11.5
3391.800	3391.7963	$X_{1/2}$	$X_{3/2}$	2	1	38.5	38.5

Table S1 (continued)

ν_{exp} (cm^{-1})	ν_{the} (cm^{-1})	GUQ	GLQ	v'	v''	J'	J''
3393.414	3393.4149	$X_{3/2}$	$X_{3/2}$	4	3	20.5	19.5
3394.369	3394.3732	$X_{3/2}$	$X_{3/2}$	3	2	5.5	4.5
3394.895	3394.8970	$X_{1/2}$	$X_{1/2}$	4	3	18.5	17.5
3395.077	3395.0742	$X_{1/2}$	$X_{1/2}$	4	3	17.5	16.5
3395.246	3395.2410	$X_{3/2}$	$X_{3/2}$	2	1	3.5	3.5
3395.450	3395.4496	$X_{3/2}$	$X_{3/2}$	4	3	18.5	17.5
3396.050	3396.0467	$X_{3/2}$	$X_{3/2}$	2	1	3.5	3.5
3397.213	3397.2118	$X_{1/2}$	$X_{1/2}$	2	1	2.5	2.5
3400.085	3400.0853	$X_{3/2}$	$X_{3/2}$	2	1	2.5	2.5
3400.751	3400.7502	$X_{1/2}$	$X_{1/2}$	2	1	1.5	1.5
3402.819	3402.8225	$X_{3/2}$	$X_{3/2}$	2	1	1.5	1.5
3402.927	3402.9322	$X_{1/2}$	$X_{1/2}$	1	0	42.5	41.5
3407.375	3407.3767	$X_{3/2}$	$X_{3/2}$	3	2	32.5	31.5
3407.613	3407.6117	$X_{3/2}$	$X_{3/2}$	1	0	5.5	4.5
3407.986	3407.9885	$X_{3/2}$	$X_{3/2}$	1	0	5.5	4.5
3412.779	3412.7785	$X_{1/2}$	$X_{3/2}$	4	3	14.5	14.5
3421.927	3421.9322	$X_{1/2}$	$X_{1/2}$	1	0	4.5	3.5
3424.086	3424.0852	$X_{1/2}$	$X_{3/2}$	6	5	13.5	12.5
3434.061	3434.0635	$X_{1/2}$	$X_{1/2}$	12	10	2.5	1.5
3434.645	3434.6455	$X_{3/2}$	$X_{3/2}$	1	0	13.5	13.5
3436.804	3436.7992	$X_{1/2}$	$X_{1/2}$	3	2	6.5	5.5
3442.181	3442.1792	$X_{3/2}$	$X_{1/2}$	1	0	1.5	0.5
3447.016	3447.0115	$X_{3/2}$	$X_{3/2}$	1	0	4.5	3.5
3453.713	3453.7093	$X_{1/2}$	$X_{1/2}$	2	1	36.5	35.5
3459.906	3459.9029	$X_{1/2}$	$X_{1/2}$	12	10	10.5	9.5
3462.589	3462.5901	$X_{1/2}$	$X_{3/2}$	3	2	4.5	4.5
3464.506	3464.5002	$X_{3/2}$	$X_{1/2}$	11	9	7.5	6.5
3470.341	3470.3419	$X_{1/2}$	$X_{1/2}$	12	10	0.5	0.5
3481.673	3481.6739	$X_{1/2}$	$X_{1/2}$	3	2	28.5	27.5
3484.594	3484.5983	$X_{3/2}$	$X_{3/2}$	1	0	3.5	2.5
3484.749	3484.7482	$X_{3/2}$	$X_{3/2}$	1	0	3.5	2.5
3489.092	3489.0907	$X_{3/2}$	$X_{3/2}$	2	1	36.5	35.5
3489.472	3489.4758	$X_{1/2}$	$X_{1/2}$	2	1	35.5	34.5
3493.001	3493.0005	$X_{3/2}$	$X_{1/2}$	10	8	12.5	11.5
3494.127	3494.1248	$X_{1/2}$	$X_{1/2}$	2	1	2.5	1.5
3502.001	3502.0002	$X_{1/2}$	$X_{1/2}$	3	2	27.5	26.5
3509.564	3509.5633	$X_{3/2}$	$X_{3/2}$	2	1	3.5	2.5
3510.822	3510.8165	$X_{3/2}$	$X_{1/2}$	9	7	16.5	15.5
3512.081	3512.0802	$X_{1/2}$	$X_{1/2}$	11	9	10.5	9.5
3512.465	3512.4673	$X_{1/2}$	$X_{3/2}$	3	2	7.5	7.5
3516.726	3516.7239	$X_{3/2}$	$X_{3/2}$	12	10	2.5	1.5
3532.405	3532.4007	$X_{3/2}$	$X_{3/2}$	1	0	7.5	7.5
3534.956	3534.9508	$X_{1/2}$	$X_{1/2}$	3	2	25.5	24.5

Table S1 (continued)

ν_{exp} (cm^{-1})	ν_{the} (cm^{-1})	GUQ	GLQ	v'	v''	J'	J''
3537.868	3537.8657	$X_{3/2}$	$X_{3/2}$	2	1	4.5	3.5
3541.038	3541.0400	$X_{3/2}$	$X_{3/2}$	3	1	41.5	40.5
3548.676	3548.6793	$X_{3/2}$	$X_{1/2}$	10	8	14.5	14.5
3556.264	3556.2615	$X_{1/2}$	$X_{3/2}$	3	2	3.5	2.5
3556.818	3556.8180	$X_{3/2}$	$X_{3/2}$	3	2	14.5	13.5
3557.133	3557.1361	$X_{3/2}$	$X_{3/2}$	2	1	34.5	33.5
3559.444	3559.4469	$X_{3/2}$	$X_{3/2}$	3	2	24.5	23.5
3564.867	3564.8711	$X_{3/2}$	$X_{3/2}$	2	1	5.5	4.5
3565.672	3565.6678	$X_{3/2}$	$X_{3/2}$	1	0	2.5	2.5
3566.080	3566.0815	$X_{1/2}$	$X_{1/2}$	11	9	9.5	8.5
3568.416	3568.4159	$X_{3/2}$	$X_{3/2}$	1	0	1.5	1.5
3568.523	3568.5228	$X_{3/2}$	$X_{3/2}$	1	0	1.5	1.5
3568.909	3568.9058	$X_{1/2}$	$X_{1/2}$	1	0	0.5	0.5
3569.213	3569.2145	$X_{1/2}$	$X_{1/2}$	1	0	0.5	0.5
3571.168	3571.1709	$X_{1/2}$	$X_{3/2}$	4	3	7.5	6.5
3578.154	3578.1589	$X_{1/2}$	$X_{1/2}$	3	2	16.5	15.5
3578.672	3578.6759	$X_{3/2}$	$X_{3/2}$	3	2	17.5	16.5
3582.911	3582.9115	$X_{1/2}$	$X_{3/2}$	6	5	23.5	22.5
3586.253	3586.2484	$X_{3/2}$	$X_{3/2}$	1	0	39.5	38.5
3588.743	3588.7436	$X_{1/2}$	$X_{1/2}$	10	8	15.5	14.5
3591.748	3591.7523	$X_{3/2}$	$X_{1/2}$	2	0	41.5	41.5

Table S2: Observed and theoretically predicted rotational lines of OH in the ground and the first excited vibrational state

ν_{exp} (cm^{-1})	ν_{the} (cm^{-1})	GUQ	GLQ	v'	v''	J'	J''
710.139	710.1364	$X_{1/2}$	$X_{1/2}$	0	0	20.5	19.5
710.774	710.7714	$X_{3/2}$	$X_{3/2}$	0	0	21.5	20.5
711.196	711.1886	$X_{1/2}$	$X_{1/2}$	0	0	20.5	19.5
712.000	711.9951	$X_{3/2}$	$X_{3/2}$	0	0	21.5	20.5
737.487	737.4856	$X_{1/2}$	$X_{1/2}$	0	0	21.5	20.5
738.052	738.0500	$X_{3/2}$	$X_{3/2}$	0	0	22.5	21.5
738.556	738.5517	$X_{1/2}$	$X_{1/2}$	0	0	21.5	20.5
739.285	739.2818	$X_{3/2}$	$X_{3/2}$	0	0	22.5	21.5
757.366	757.3654	$X_{1/2}$	$X_{1/2}$	1	1	23.5	22.5
757.838	757.8394	$X_{3/2}$	$X_{3/2}$	1	1	24.5	23.5
758.997	758.9941	$X_{3/2}$	$X_{3/2}$	1	1	24.5	23.5
763.983	763.9828	$X_{1/2}$	$X_{1/2}$	0	0	22.5	21.5
764.484	764.4861	$X_{3/2}$	$X_{3/2}$	0	0	23.5	22.5
765.058	765.0580	$X_{1/2}$	$X_{1/2}$	0	0	22.5	21.5
765.719	765.7208	$X_{3/2}$	$X_{3/2}$	0	0	23.5	22.5
780.877	780.8806	$X_{1/2}$	$X_{1/2}$	1	1	24.5	23.5
781.302	781.3067	$X_{3/2}$	$X_{3/2}$	1	1	25.5	24.5
781.889	781.8884	$X_{1/2}$	$X_{1/2}$	1	1	24.5	23.5
782.450	782.4516	$X_{3/2}$	$X_{3/2}$	1	1	25.5	24.5
789.600	789.6035	$X_{1/2}$	$X_{1/2}$	0	0	23.5	22.5
790.050	790.0534	$X_{3/2}$	$X_{3/2}$	0	0	24.5	23.5
790.680	790.6827	$X_{1/2}$	$X_{1/2}$	0	0	23.5	22.5
791.281	791.2860	$X_{3/2}$	$X_{3/2}$	0	0	24.5	23.5
803.484	803.4846	$X_{1/2}$	$X_{1/2}$	1	1	25.5	24.5
803.863	803.8687	$X_{3/2}$	$X_{3/2}$	1	1	26.5	25.5
814.319	814.3239	$X_{1/2}$	$X_{1/2}$	0	0	24.5	23.5
814.722	814.7272	$X_{3/2}$	$X_{3/2}$	0	0	25.5	24.5
815.398	815.4022	$X_{1/2}$	$X_{1/2}$	0	0	24.5	23.5
815.948	815.9525	$X_{3/2}$	$X_{3/2}$	0	0	25.5	24.5
825.147	825.1554	$X_{1/2}$	$X_{1/2}$	1	1	26.5	25.5
825.497	825.5026	$X_{3/2}$	$X_{3/2}$	1	1	27.5	26.5
826.135	826.1420	$X_{1/2}$	$X_{1/2}$	1	1	26.5	25.5
838.116	838.1217	$X_{1/2}$	$X_{1/2}$	0	0	25.5	24.5
838.480	838.4842	$X_{3/2}$	$X_{3/2}$	0	0	26.5	25.5
839.189	839.1942	$X_{1/2}$	$X_{1/2}$	0	0	25.5	24.5
839.691	839.6970	$X_{3/2}$	$X_{3/2}$	0	0	26.5	25.5
860.970	860.9758	$X_{1/2}$	$X_{1/2}$	0	0	26.5	25.5
861.298	861.3024	$X_{3/2}$	$X_{3/2}$	0	0	27.5	26.5
862.032	862.0374	$X_{1/2}$	$X_{1/2}$	0	0	26.5	25.5
862.493	862.4976	$X_{3/2}$	$X_{3/2}$	0	0	27.5	26.5
882.862	882.8660	$X_{1/2}$	$X_{1/2}$	0	0	27.5	26.5
883.157	883.1610	$X_{3/2}$	$X_{3/2}$	0	0	28.5	27.5
883.910	883.9119	$X_{1/2}$	$X_{1/2}$	0	0	27.5	26.5

Table S2 (continued)

ν_{exp} (cm^{-1})	ν_{the} (cm^{-1})	GUQ	GLQ	v'	v''	J'	J''
884.330	884.3335	$X_{3/2}$	$X_{3/2}$	0	0	28.5	27.5
903.769	903.7735	$X_{1/2}$	$X_{1/2}$	0	0	28.5	27.5
904.036	904.0406	$X_{3/2}$	$X_{3/2}$	0	0	29.5	28.5
904.795	904.7988	$X_{1/2}$	$X_{1/2}$	0	0	28.5	27.5
905.179	905.1854	$X_{3/2}$	$X_{3/2}$	0	0	29.5	28.5
923.678	923.6805	$X_{1/2}$	$X_{1/2}$	0	0	29.5	28.5
923.919	923.9230	$X_{3/2}$	$X_{3/2}$	0	0	30.5	29.5
924.680	924.6804	$X_{1/2}$	$X_{1/2}$	0	0	29.5	28.5
925.033	925.0350	$X_{3/2}$	$X_{3/2}$	0	0	30.5	29.5
942.571	942.5702	$X_{1/2}$	$X_{1/2}$	0	0	30.5	29.5
942.788	942.7908	$X_{3/2}$	$X_{3/2}$	0	0	31.5	30.5
943.540	943.5400	$X_{1/2}$	$X_{1/2}$	0	0	30.5	29.5
943.862	943.8652	$X_{3/2}$	$X_{3/2}$	0	0	31.5	30.5
960.626	960.6281	$X_{3/2}$	$X_{3/2}$	0	0	32.5	31.5
961.651	961.6600	$X_{3/2}$	$X_{3/2}$	0	0	32.5	31.5

Table S3: Rovibrational emission lines of NH radical in the $X^3\Sigma^-$ ground state

ν_{exp} (cm^{-1})	ν_{the} (cm^{-1})	v'	v''	J'	N'	J''	N''
2426.441	2426.4392	1	0	6	5	7	8
2613.714	2613.7140	1	0	13	12	13	13
2613.955	2613.9517	1	0	13	12	12	13
2660.131	2660.1320	1	0	11	11	12	12
2704.314	2704.3094	1	0	11	10	12	11
2730.722	2730.7179	1	0	3	2	4	5
2746.092	2746.0923	1	0	10	9	10	10
2747.407	2747.4052	1	0	8	9	9	10
2747.488	2747.4922	1	0	9	9	10	10
2790.380	2790.3822	1	0	8	8	8	9
2830.755	2830.7539	1	0	2	1	3	4
2831.956	2831.9537	1	0	7	7	7	8
2910.371	2910.3732	1	0	6	5	6	6
2912.205	2912.2076	1	0	5	5	5	6
2929.953	2929.9560	1	0	1	0	2	3
2948.966	2948.9613	1	0	5	4	5	5
2949.960	2949.9660	1	0	3	4	4	5
2987.381	2987.3859	1	0	2	3	3	4
2987.585	2987.5859	1	0	4	3	5	4
3022.865	3022.8649	1	0	3	2	3	3
3023.638	3023.6333	1	0	1	2	2	3
3023.903	3023.9063	1	0	3	2	4	3
3058.955	3058.9574	1	0	1	1	2	2
3059.064	3059.0697	1	0	2	1	3	2
3059.219	3059.2203	1	0	2	1	1	2
3092.211	3092.2164	1	0	1	0	1	1
3093.062	3093.0676	1	0	1	0	2	1
3093.995	3094.0008	1	0	1	0	0	1
3156.783	3156.7860	1	0	2	1	1	0
3157.626	3157.6314	1	0	1	1	1	0
3186.908	3186.9097	1	0	3	2	2	1
3187.009	3187.0072	1	0	2	2	1	1
3187.862	3187.8585	1	0	2	2	2	1
3214.742	3214.7441	1	0	2	3	2	2
3215.609	3215.6138	1	0	4	3	3	2
3215.685	3215.6856	1	0	3	3	2	2
3215.846	3215.8524	1	0	2	3	1	2
3242.888	3242.8927	1	0	5	4	4	3
3242.951	3242.9511	1	0	4	4	3	3
3243.044	3243.0462	1	0	3	4	2	3
3243.158	3243.1544	1	0	3	4	4	3
3268.713	3268.7153	1	0	6	5	5	4
3268.766	3268.7652	1	0	5	5	4	4

Table S3 (continued)

ν_{exp} (cm^{-1})	ν_{the} (cm^{-1})	v'	v''	J'	N'	J''	N''
3268.835	3268.8345	1	0	4	5	3	4
3269.880	3269.8799	1	0	5	5	5	4
3293.045	3293.0463	1	0	7	6	6	5
3293.093	3293.0899	1	0	6	6	5	5
3293.150	3293.1459	1	0	5	6	4	5
3293.587	3293.5852	1	0	5	6	6	5
3294.275	3294.2723	1	0	6	6	6	5
3316.511	3316.5122	1	0	6	7	7	6
3356.260	3356.2542	1	0	8	9	8	8
3374.306	3374.3103	1	0	9	10	9	9
3392.182	3392.1828	1	0	10	11	11	10
3405.374	3405.3722	1	0	11	12	11	11
3418.608	3418.6059	1	0	12	13	11	12
3419.911	3419.9145	1	0	12	13	13	12
3429.679	3429.6822	1	0	15	14	14	13
3438.838	3438.8322	1	0	14	15	14	14
2386.134	2386.1334	2	1	13	14	14	15
2386.399	2386.3940	2	1	14	14	14	15
2431.176	2431.1783	2	1	12	13	13	14
2431.276	2431.2701	2	1	13	13	14	14
2474.373	2474.3736	2	1	13	12	12	13
2475.713	2475.7185	2	1	13	12	14	13
2475.897	2475.9000	2	1	12	12	12	13
2493.045	2493.0506	2	1	4	3	5	6
2560.661	2560.6629	2	1	11	10	11	11
2603.170	2603.1756	2	1	10	9	9	10
2604.015	2604.0180	2	1	8	9	9	10
2604.101	2604.1007	2	1	9	9	10	10
2604.193	2604.1876	2	1	10	9	11	10
2644.432	2644.4338	2	1	9	8	8	9
2684.784	2684.7809	2	1	8	7	7	8
2725.377	2725.3785	2	1	6	6	6	7
2781.452	2781.4558	2	1	1	0	2	3
2800.338	2800.3438	2	1	5	4	6	5
2835.389	2835.3923	2	1	4	3	4	4
2836.298	2836.2985	2	1	2	3	3	4
2836.405	2836.4089	2	1	3	3	4	4
2836.490	2836.4922	2	1	4	3	5	4
2871.245	2871.2509	2	1	1	2	2	3
2904.442	2904.4363	2	1	2	1	2	2
2904.605	2904.6014	2	1	0	1	1	2
2905.271	2905.2748	2	1	1	1	2	2
2905.380	2905.3851	2	1	2	1	3	2

Table S3 (continued)

ν_{exp} (cm^{-1})	ν_{the} (cm^{-1})	v'	v''	J'	N'	J''	N''
2998.279	2998.2787	2	1	0	1	1	0
2999.227	2999.2271	2	1	2	1	1	0
3000.069	3000.0657	2	1	1	1	1	0
3028.057	3028.0614	2	1	3	2	2	1
3028.151	3028.1548	2	1	2	2	1	1
3028.827	3028.8246	2	1	1	2	0	1
3029.005	3029.0002	2	1	2	2	2	1
3055.473	3055.4786	2	1	4	3	3	2
3055.544	3055.5462	2	1	3	3	2	2
3055.711	3055.7114	2	1	2	3	1	2
3080.684	3080.6795	2	1	3	4	3	3
3081.526	3081.5276	2	1	4	4	3	3
3081.620	3081.6210	2	1	3	4	2	3
3081.710	3081.7089	2	1	3	4	4	3
3106.060	3106.0602	2	1	5	5	4	4
3106.130	3106.1279	2	1	4	5	3	4
3106.384	3106.3896	2	1	4	5	5	4
3107.155	3107.1600	2	1	5	5	5	4
3129.067	3129.0666	2	1	7	6	6	5
3129.108	3129.1061	2	1	6	6	5	5
3129.165	3129.1604	2	1	5	6	4	5
3129.566	3129.5675	2	1	5	6	6	5
3130.269	3130.2707	2	1	6	6	6	5
3150.599	3150.5931	2	1	8	7	7	6
3151.217	3151.2121	2	1	6	7	7	6
3170.009	3170.0042	2	1	7	8	7	7
3205.202	3205.2024	2	1	9	10	9	9
3220.307	3220.3113	2	1	10	11	10	10
3221.767	3221.7671	2	1	10	11	11	10
3233.717	3233.7129	2	1	11	12	11	11
3245.375	3245.3723	2	1	12	13	12	12
3255.502	3255.5070	2	1	15	14	14	13
3257.142	3257.1363	2	1	14	14	14	13
3410.692	3410.6969	2	1	5	6	4	3
2335.019	2335.0185	3	2	11	12	12	13
2335.108	2335.1029	3	2	12	12	13	13
2419.162	2419.1672	3	2	9	10	10	11
2419.245	2419.2471	3	2	10	10	11	11
2419.668	2419.6651	3	2	10	10	10	11
2448.896	2448.8957	3	2	3	2	4	5
2460.003	2459.9970	3	2	8	9	9	10
2460.158	2460.1595	3	2	10	9	11	10
2460.545	2460.5468	3	2	9	9	9	10

Table S3 (continued)

ν_{exp} (cm^{-1})	ν_{the} (cm^{-1})	v'	v''	J'	N'	J''	N''
2541.186	2541.1894	3	2	2	1	3	4
2577.716	2577.7135	3	2	6	6	6	7
2613.679	2613.6794	3	2	6	5	5	6
2649.011	2649.0113	3	2	5	4	5	5
2649.987	2649.9923	3	2	3	4	4	5
2650.152	2650.1569	3	2	5	4	6	5
2684.850	2684.8539	3	2	2	3	3	4
2717.777	2717.7766	3	2	3	2	3	3
2718.791	2718.7931	3	2	3	2	4	3
2751.273	2751.2731	3	2	1	1	2	2
2751.379	2751.3811	3	2	2	1	3	2
2782.795	2782.7976	3	2	1	0	2	1
2840.398	2840.4008	3	2	0	1	1	0
2842.184	2842.1855	3	2	1	1	1	0
2868.978	2868.9761	3	2	2	2	1	1
2894.992	2894.9966	3	2	4	3	3	2
2895.063	2895.0605	3	2	3	3	2	2
2895.222	2895.2239	3	2	2	3	1	2
2918.874	2918.8732	3	2	3	4	3	3
2919.726	2919.7298	3	2	4	4	3	3
2919.820	2919.8217	3	2	3	4	2	3
2942.159	2942.1633	3	2	4	5	4	4
2942.900	2942.9031	3	2	6	5	5	4
2942.948	2942.9452	3	2	5	5	4	4
2943.010	2943.0114	3	2	4	5	3	4
2964.634	2964.6324	3	2	7	6	6	5
2964.727	2964.7212	3	2	5	6	4	5
2984.831	2984.8299	3	2	8	7	7	6
2984.863	2984.8607	3	2	7	7	6	6
2985.022	2985.0191	3	2	2	3	1	0
3003.485	3003.4845	3	2	8	8	7	7
3004.145	3004.1417	3	2	7	8	8	7
3021.820	3021.8172	3	2	9	9	9	8
3049.557	3049.5532	3	2	12	11	11	10
3050.538	3050.5434	3	2	10	11	11	10
3061.547	3061.5434	3	2	12	12	11	11
3063.016	3063.0178	3	2	12	12	12	11
3071.775	3071.7814	3	2	12	13	11	12
3073.285	3073.2902	3	2	13	13	13	12
3081.470	3081.4731	3	2	13	14	14	13
3314.128	3314.1288	3	2	6	7	5	4
2306.418	2306.4227	4	3	3	2	4	5
2314.184	2314.1859	4	3	8	9	9	10

Table S3 (continued)

ν_{exp} (cm^{-1})	ν_{the} (cm^{-1})	v'	v''	J'	N'	J''	N''
2314.261	2314.2588	4	3	9	9	10	10
2352.363	2352.3680	4	3	9	8	8	9
2353.007	2353.0034	4	3	7	8	8	9
2464.352	2464.3570	4	3	5	5	5	6
2499.183	2499.1822	4	3	4	4	4	5
2532.045	2532.0478	4	3	3	3	4	4
2532.128	2532.1276	4	3	4	3	5	4
2564.627	2564.6292	4	3	3	2	4	3
2595.183	2595.1847	4	3	0	1	1	2
2625.256	2625.2531	4	3	1	0	1	1
2626.080	2626.0840	4	3	1	0	2	1
2682.858	2682.8609	4	3	1	1	1	0
2707.212	2707.2111	4	3	1	2	1	1
2708.247	2708.2447	4	3	3	2	2	1
2708.326	2708.3296	4	3	2	2	1	1
2733.009	2733.0137	4	3	4	3	3	2
2733.074	2733.0729	4	3	3	3	2	2
2733.233	2733.2354	4	3	2	3	1	2
2755.529	2755.5233	4	3	3	4	3	3
2756.337	2756.3400	4	3	5	4	4	3
2756.382	2756.3860	4	3	4	4	3	3
2756.479	2756.4771	4	3	3	4	2	3
2778.192	2778.1918	4	3	6	5	5	4
2778.229	2778.2293	4	3	5	5	4	4
2778.294	2778.2948	4	3	4	5	3	4
2799.685	2799.6894	4	3	6	6	6	5
2817.346	2817.3492	4	3	7	7	6	6
2834.549	2834.5471	4	3	8	8	7	7
2835.776	2835.7819	4	3	8	8	8	7
2876.208	2876.2100	4	3	12	11	11	10
2876.239	2876.2449	4	3	10	11	9	10
2886.671	2886.6671	4	3	13	12	12	11
2902.189	2902.1900	4	3	15	14	14	13
2903.731	2903.7259	4	3	14	14	14	13
2979.395	2979.3914	4	3	4	5	3	2
3132.334	3132.3324	4	3	6	7	5	4
3538.652	3538.6484	4	3	12	13	11	10
3597.483	3597.4828	4	3	13	14	12	11
2309.128	2309.1232	5	4	6	5	6	6
2310.136	2310.1374	5	4	4	5	5	6
2310.197	2310.2029	5	4	5	5	6	6
2343.867	2343.8731	5	4	5	4	6	5
2376.243	2376.2440	5	4	3	3	4	4

Table S3 (continued)

ν_{exp} (cm^{-1})	ν_{the} (cm^{-1})	v'	v''	J'	N'	J''	N''
2407.516	2407.5105	5	4	2	2	3	3
2407.597	2407.5979	5	4	3	2	4	3
2467.470	2467.4677	5	4	1	0	0	1
2518.872	2518.8687	5	4	0	1	1	0
2543.622	2543.6275	5	4	1	2	1	1
2544.450	2544.4492	5	4	1	2	2	1
2544.657	2544.6614	5	4	3	2	2	1
2544.740	2544.7396	5	4	2	2	1	1
2545.401	2545.4058	5	4	1	2	0	1
2568.038	2568.0351	5	4	4	3	3	2
2568.253	2568.2519	5	4	2	3	1	2
2589.108	2589.1131	5	4	3	4	3	3
2590.967	2590.9640	5	4	4	4	4	3
2610.362	2610.3661	5	4	5	5	4	4
2610.616	2610.6196	5	4	4	5	5	4
2628.478	2628.4783	5	4	5	6	5	5
2629.272	2629.2694	5	4	5	6	4	5
2629.588	2629.5829	5	4	5	6	6	5
2645.795	2645.7992	5	4	6	7	6	6
2646.483	2646.4831	5	4	7	7	6	6
2646.526	2646.5289	5	4	6	7	5	6
2650.205	2650.2098	5	4	2	3	1	0
2662.108	2662.1132	5	4	9	8	8	7
2662.173	2662.1683	5	4	7	8	6	7
2663.334	2663.3363	5	4	8	8	8	7
2675.524	2675.5241	5	4	8	9	8	8
2676.115	2676.1108	5	4	9	9	8	8
2676.145	2676.1454	5	4	8	9	7	8
2676.776	2676.7808	5	4	8	9	9	8
2677.371	2677.3674	5	4	9	9	9	8
2698.423	2698.4225	5	4	10	11	10	10
2698.945	2698.9442	5	4	10	11	9	10
2707.680	2707.6819	5	4	11	12	10	11
2715.609	2715.6068	5	4	12	13	13	12
2723.900	2723.8988	5	4	15	16	14	15
2727.161	2727.1638	5	4	3	4	2	1
2802.332	2802.3304	5	4	4	5	3	2
3016.314	3016.3088	5	4	7	8	6	5

Table S4: Emission lines of the CN radical for the $X^2\Sigma^+$ fundamental ground state

ν (cm^{-1})	J'	J''	v'	v''	ν (cm^{-1})	J'	J''	v'	v''
1928.5855	26	27	1	0	2101.2625	17	16	1	0
1933.2020	25	26	1	0	2104.3954	18	17	1	0
1937.8149	24	25	1	0	2107.4877	19	18	1	0
1942.3878	23	24	1	0	2110.5439	20	19	1	0
1946.9344	22	23	1	0	2112.5613	21	20	1	0
1951.4403	21	22	1	0	2116.5377	22	21	1	0
1955.9139	20	21	1	0	2119.4735	23	22	1	0
1960.3640	19	20	1	0	2122.3717	24	23	1	0
1964.7830	18	19	1	0	2125.2563	25	24	1	0
1969.1610	17	18	1	0	2128.1161	26	25	1	0
1973.5134	16	17	1	0	2130.8628	27	26	1	0
1977.8299	15	16	1	0	2133.5737	28	27	1	0
1982.1188	14	15	1	0	1912.3507	24	25	2	1
1986.3719	13	14	1	0	1916.8967	23	24	2	1
1990.5944	12	13	1	0	1921.4114	22	23	2	1
1994.7822	11	12	1	0	1925.8873	21	22	2	1
1998.9378	10	11	1	0	1930.3330	20	21	2	1
2003.0601	9	10	1	0	1934.7503	19	20	2	1
2007.1483	8	9	1	0	1939.1278	18	19	2	1
2011.2042	7	8	1	0	1943.4779	17	18	2	1
2015.2257	6	7	1	0	1947.7994	16	17	2	1
2019.2137	5	6	1	0	1952.0832	15	16	2	1
2023.1678	4	5	1	0	1956.3365	14	15	2	1
2027.0870	3	4	1	0	1960.5572	13	14	2	1
2030.9735	2	3	1	0	1964.7432	12	13	2	1
2034.8172	1	2	1	0	1968.8991	11	12	2	1
2038.6415	0	1	1	0	1973.0215	10	11	2	1
2046.1731	1	0	1	0	1977.1091	9	10	2	1
2049.8840	2	1	1	0	1981.1653	8	9	2	1
2053.5619	3	2	1	0	1985.1861	7	8	2	1
2057.2034	4	3	1	0	1989.1737	6	7	2	1
2060.8098	5	4	1	0	1993.1274	5	6	2	1
2064.3806	6	5	1	0	1997.0467	4	5	2	1
2067.9153	7	6	1	0	2000.9327	3	4	2	1
2071.4142	8	7	1	0	2004.7752	2	3	2	1
2074.8768	9	8	1	0	2008.6006	1	2	2	1
2078.3038	10	9	1	0	2012.3840	0	1	2	1
2081.6943	11	10	1	0	2019.8438	1	0	2	1
2085.0476	12	11	1	0	2023.5209	2	1	2	1
2088.3645	13	12	1	0	2027.1632	3	2	2	1
2091.6447	14	13	1	0	2030.7691	4	3	2	1
2094.8878	15	14	1	0	2034.3397	5	4	2	1
2098.0937	16	15	1	0	2037.8752	6	5	2	1

Table S4 (continued)

ν (cm^{-1})	J'	J''	v'	v''	ν (cm^{-1})	J'	J''	v'	v''
2041.3746	7	6	2	1	1993.4751	1	0	3	2
2044.8378	8	7	2	1	1997.1204	2	1	3	2
2048.2649	9	8	2	1	2000.7248	3	2	3	2
2051.6558	10	9	2	1	2004.2952	4	3	3	2
2055.0098	11	10	2	1	2007.8314	5	4	3	2
2058.3277	12	11	2	1	2011.3309	6	5	3	2
2061.6090	13	12	2	1	2014.7942	7	6	3	2
2064.8485	14	13	2	1	2018.2215	8	7	3	2
2068.0587	15	14	2	1	2021.6128	9	8	3	2
2071.2298	16	15	2	1	2024.9676	10	9	3	2
2074.3594	17	16	2	1	2028.2849	11	10	3	2
2077.4544	18	17	2	1	2031.5668	12	11	3	2
2080.5117	19	18	2	1	2034.8172	13	12	3	2
2083.5320	20	19	2	1	2038.0184	14	13	3	2
2086.5050	21	20	2	1	2041.1879	15	14	3	2
2089.4503	22	21	2	1	2044.3201	16	15	3	2
2092.3576	23	22	2	1	2047.4164	17	16	3	2
2095.2542	24	23	2	1	2050.4704	18	17	3	2
2098.0491	25	24	2	1	2053.4899	19	18	3	2
2100.8117	26	25	2	1	2056.4728	20	19	3	2
2103.5892	27	26	2	1	2059.4119	21	20	3	2
2106.2607	28	27	2	1	2062.3248	22	21	3	2
2108.9338	29	28	2	1	2065.1719	23	22	3	2
2111.5424	30	29	2	1	2068.0104	24	23	3	2
2114.2053	31	30	2	1	2070.7981	25	24	3	2
1913.4396	18	19	3	2	2073.5051	26	25	3	2
1917.7724	17	18	3	2	2076.2609	27	26	3	2
1922.0434	16	17	3	2	2078.9582	28	27	3	2
1926.2969	15	16	3	2	2081.5644	29	28	3	2
1930.5166	14	15	3	2	2084.1264	30	29	3	2
1938.8586	12	13	3	2	1879.0565	20	21	4	3
1942.9789	11	12	3	2	1883.3974	19	20	4	3
1947.0669	10	11	3	2	1887.6986	18	19	4	3
1951.1218	9	10	3	2	1891.9962	17	18	4	3
1955.1433	8	9	3	2	1896.2530	16	17	4	3
1959.1301	7	8	3	2	1900.4697	15	16	4	3
1963.0831	6	7	3	2	1904.6590	14	15	4	3
1967.0031	5	6	3	2	1908.8107	13	14	4	3
1970.8884	4	5	3	2	1912.9321	12	13	4	3
1974.7401	3	4	3	2	1917.0201	11	12	4	3
1978.5553	2	3	3	2	1921.0744	10	11	4	3
1982.3364	1	2	3	2	1925.0944	9	10	4	3
1986.0849	0	1	3	2	1933.0352	7	8	4	3

Table S4 (continued)

ν (cm^{-1})	J'	J''	v'	v''	ν (cm^{-1})	J'	J''	v'	v''
1936.9546	6	7	4	3	1891.0186	11	12	5	4
1940.8393	5	6	4	3	1895.0415	10	11	5	4
1944.6911	4	5	4	3	1899.0268	9	10	5	4
1948.5072	3	4	4	3	1902.9792	8	9	5	4
1952.2885	2	3	4	3	1906.8983	7	8	5	4
1956.0374	1	2	4	3	1910.7842	6	7	5	4
1959.7519	0	1	4	3	1914.6344	5	6	5	4
1967.0700	1	0	4	3	1918.4500	4	5	5	4
1970.6745	2	1	4	3	1922.2334	3	4	5	4
1974.2471	3	2	4	3	1925.9797	2	3	5	4
1977.7820	4	3	4	3	1929.6927	1	2	5	4
1981.2823	5	4	4	3	1933.3707	0	1	5	4
1984.7451	6	5	4	3	1940.6144	1	0	5	4
1988.1732	7	6	4	3	1944.1887	2	1	5	4
1991.5644	8	7	4	3	1947.7257	3	2	5	4
1994.9192	9	8	4	3	1951.2274	4	3	5	4
1998.2386	10	9	4	3	1954.6896	5	4	5	4
2001.5185	11	10	4	3	1958.1176	6	5	5	4
2004.7751	12	11	4	3	1961.5093	7	6	5	4
2007.9730	13	12	4	3	1964.8646	8	7	5	4
2011.1463	14	13	4	3	1968.1830	9	8	5	4
2014.2751	15	14	4	3	1971.4660	10	9	5	4
2017.3701	16	15	4	3	1974.7401	11	10	5	4
2020.4280	17	16	4	3	1977.9176	12	11	5	4
2023.4488	18	17	4	3	1981.0874	13	12	5	4
2026.4283	19	18	4	3	1984.2228	14	13	5	4
2029.3747	20	19	4	3	1987.3204	15	14	5	4
2032.2860	21	20	4	3	1990.3789	16	15	5	4
2035.1494	22	21	4	3	1993.3984	17	16	5	4
2037.9720	23	22	4	3	1996.3794	18	17	5	4
2040.7559	24	23	4	3	1999.3226	19	18	5	4
2043.5171	25	24	4	3	2002.2282	20	19	5	4
2046.2237	26	25	4	3	2005.1024	21	20	5	4
2048.8977	27	26	4	3	2007.9237	22	21	5	4
1853.3519	20	21	5	4	2010.7229	23	22	5	4
1857.6627	19	20	5	4	2013.4075	24	23	5	4
1861.9471	18	19	5	4	2016.1979	25	24	5	4
1866.2023	17	18	5	4	2018.8718	26	25	5	4
1870.4164	16	17	5	4	1844.5397	16	17	6	5
1874.5996	15	16	5	4	1848.6991	15	16	6	5
1878.7577	14	15	5	4	1852.8143	14	15	6	5
1882.8797	13	14	5	4	1856.9055	13	14	6	5
1886.9652	12	13	5	4	1860.9559	12	13	6	5

Table S4 (continued)

ν (cm^{-1})	J'	J''	v'	v''	ν (cm^{-1})	J'	J''	v'	v''
1864.9779	11	12	6	5	1865.8429	4	5	7	6
1872.9162	9	10	6	5	1869.5497	3	4	7	6
1876.8354	8	9	6	5	1873.2297	2	3	7	6
1880.7213	7	8	6	5	1876.8761	1	2	7	6
1884.5728	6	7	6	5	1887.5802	1	0	7	6
1888.3869	5	6	6	5	1891.0880	2	1	7	6
1892.1679	4	5	6	5	1894.5505	3	2	7	6
1895.9158	3	4	6	5	1897.9789	4	3	7	6
1899.6268	2	3	6	5	1901.3698	5	4	7	6
1903.3036	1	2	6	5	1904.7246	6	5	7	6
1906.9448	0	1	6	5	1908.0437	7	6	7	6
1914.1238	1	0	6	5	1911.3281	8	7	7	6
1917.6601	2	1	6	5	1914.5755	9	8	7	6
1921.1622	3	2	6	5	1917.7724	10	9	7	6
1924.6259	4	3	6	5	1920.9524	11	10	7	6
1928.0538	5	4	6	5	1924.0855	12	11	7	6
1931.4453	6	5	6	5	1927.1844	13	12	7	6
1934.8021	7	6	6	5	1930.2394	14	13	7	6
1938.1189	8	7	6	5	1933.2620	15	14	7	6
1941.4018	9	8	6	5	1936.2485	16	15	7	6
1947.8548	11	10	6	5	1939.1858	17	16	7	6
1951.0282	12	11	6	5	1942.1069	18	17	7	6
1954.1597	13	12	6	5	1944.9719	19	18	7	6
1957.2567	14	13	6	5	1947.7994	20	19	7	6
1960.3161	15	14	6	5	1950.5953	21	20	7	6
1963.3356	16	15	6	5	1816.6909	10	11	8	7
1966.3226	17	16	6	5	1820.5594	9	10	8	7
1972.1678	19	18	6	5	1824.4058	8	9	8	7
1975.0354	20	19	6	5	1828.2782	7	8	8	7
1977.8728	21	20	6	5	1832.0105	6	7	8	7
1980.6772	22	21	6	5	1835.7451	5	6	8	7
1983.4130	23	22	6	5	1839.4649	4	5	8	7
1986.1304	24	23	6	5	1843.1407	3	4	8	7
1988.8102	25	24	6	5	1846.7576	2	3	8	7
1830.8573	13	14	7	6	1850.3724	1	2	8	7
1834.9072	12	13	7	6	1853.9552	0	1	8	7
1838.8903	11	12	7	6	1860.9559	1	0	8	7
1842.8405	10	11	7	6	1864.4642	2	1	8	7
1846.7576	9	10	7	6	1867.8652	3	2	8	7
1850.6460	8	9	7	6	1871.2807	4	3	8	7
1854.4941	7	8	7	6	1874.6404	5	4	8	7
1858.3114	6	7	7	6	1877.9579	6	5	8	7
1862.0958	5	6	7	6	1881.2390	7	6	8	7

Table S4 (continued)

**ν (cm^{-1})**	**J'**	**J''**	**v'**	**v''**
1884.4849	8	7	8	7
1887.6986	9	8	8	7
1890.8615	10	9	8	7
1893.9942	11	10	8	7
1897.0903	12	11	8	7
1900.1581	13	12	8	7
1903.1714	14	13	8	7
1906.1642	15	14	8	7
1909.1000	16	15	8	7
1912.0119	17	16	8	7
1914.8826	18	17	8	7

Table S5: Emission lines of the CN radical for Δv = -3 of the $A^2\Pi \rightarrow X^2\Sigma^+$ transition

ν (cm^{-1})	J'	J''	v'	v''	branch	ν (cm^{-1})	J'	J''	v'	v''	branch
3091.324	1.5	1.5	0	3	Q22	2986.156	23.5	23.5	0	3	Q11
3089.093	2.5	2.5	0	3	Q22	2980.150	24.5	24.5	0	3	Q11
3086.689	3.5	3.5	0	3	Q22	3034.942	1.5	2.5	0	3	P11
3084.118	4.5	4.5	0	3	Q22	3029.051	3.5	4.5	0	3	P11
3081.379	5.5	5.5	0	3	Q22	3025.579	4.5	5.5	0	3	P11
3078.463	6.5	6.5	0	3	Q22	3021.748	5.5	6.5	0	3	P11
3075.367	7.5	7.5	0	3	Q22	3017.571	6.5	7.5	0	3	P11
3072.083	8.5	8.5	0	3	Q22	3013.038	7.5	8.5	0	3	P11
3068.609	9.5	9.5	0	3	Q22	3008.182	8.5	9.5	0	3	P11
3064.938	10.5	10.5	0	3	Q22	3002.988	9.5	10.5	0	3	P11
3061.063	11.5	11.5	0	3	Q22	2997.465	10.5	11.5	0	3	P11
3056.982	12.5	12.5	0	3	Q22	2991.621	11.5	12.5	0	3	P11
3052.692	13.5	13.5	0	3	Q22	2985.463	12.5	13.5	0	3	P11
3048.178	14.5	14.5	0	3	Q22	2978.992	13.5	14.5	0	3	P11
3043.456	15.5	15.5	0	3	Q22	2972.218	14.5	15.5	0	3	P11
3038.498	16.5	16.5	0	3	Q22	2957.769	16.5	17.5	0	3	P11
3033.326	17.5	17.5	0	3	Q22	2950.093	17.5	18.5	0	3	P11
3027.914	18.5	18.5	0	3	Q22	2942.125	18.5	19.5	0	3	P11
3022.273	19.5	19.5	0	3	Q22	2933.869	19.5	20.5	0	3	P11
3016.400	20.5	20.5	0	3	Q22	3049.110	6.5	7.5	0	3	P22
3010.287	21.5	21.5	0	3	Q22	3042.345	7.5	8.5	0	3	P22
3003.938	22.5	22.5	0	3	Q22	3035.386	8.5	9.5	0	3	P22
3042.300	1.5	1.5	0	3	Q11	3028.248	9.5	10.5	0	3	P22
3043.211	2.5	2.5	0	3	Q11	3020.909	10.5	11.5	0	3	P22
3043.970	4.5	4.5	0	3	Q11	3013.366	11.5	12.5	0	3	P22
3043.820	5.5	5.5	0	3	Q11	3005.620	12.5	13.5	0	3	P22
3043.309	6.5	6.5	0	3	Q11	2997.666	13.5	14.5	0	3	P22
3042.466	7.5	7.5	0	3	Q11	2989.492	14.5	15.5	0	3	P22
3041.279	8.5	8.5	0	3	Q11	2981.099	15.5	16.5	0	3	P22
3039.765	9.5	9.5	0	3	Q11	2963.643	17.5	18.5	0	3	P22
3037.913	10.5	10.5	0	3	Q11	2954.589	18.5	19.5	0	3	P22
3035.750	11.5	11.5	0	3	Q11	2945.295	19.5	20.5	0	3	P22
3033.266	12.5	12.5	0	3	Q11	2935.772	20.5	21.5	0	3	P22
3030.469	13.5	13.5	0	3	Q11	2926.016	21.5	22.5	0	3	P22
3027.364	14.5	14.5	0	3	Q11	3050.577	2.5	1.5	0	3	R11
3023.958	15.5	15.5	0	3	Q11	3054.808	3.5	2.5	0	3	R11
3020.252	16.5	16.5	0	3	Q11	3058.678	4.5	3.5	0	3	R11
3016.247	17.5	17.5	0	3	Q11	3062.195	5.5	4.5	0	3	R11
3011.951	18.5	18.5	0	3	Q11	3065.367	6.5	5.5	0	3	R11
3007.361	19.5	19.5	0	3	Q11	3068.187	7.5	6.5	0	3	R11
3002.490	20.5	20.5	0	3	Q11	3070.670	8.5	7.5	0	3	R11
2997.317	21.5	21.5	0	3	Q11	3072.817	9.5	8.5	0	3	R11
2991.882	22.5	22.5	0	3	Q11	3074.633	10.5	9.5	0	3	R11

Table S5 (continued)

ν (cm^{-1})	J'	J''	v'	v''	branch	ν (cm^{-1})	J'	J''	v'	v''	branch
3076.129	11.5	10.5	0	3	R11	2902.018	13.5	12.5	1	4	R11
3077.302	12.5	11.5	0	3	R11	2902.542	14.5	13.5	1	4	R11
3078.161	13.5	12.5	0	3	R11	2902.754	15.5	14.5	1	4	R11
3078.707	14.5	13.5	0	3	R11	2902.664	16.5	15.5	1	4	R11
3078.946	15.5	14.5	0	3	R11	2902.282	17.5	16.5	1	4	R11
3078.884	16.5	15.5	0	3	R11	2901.598	18.5	17.5	1	4	R11
3078.527	17.5	16.5	0	3	R11	2900.626	19.5	18.5	1	4	R11
3077.862	18.5	17.5	0	3	R11	2899.362	20.5	19.5	1	4	R11
3076.913	19.5	18.5	0	3	R11	2897.800	21.5	20.5	1	4	R11
3075.666	20.5	19.5	0	3	R11	2895.955	22.5	21.5	1	4	R11
3074.128	21.5	20.5	0	3	R11	2893.827	23.5	22.5	1	4	R11
3072.309	22.5	21.5	0	3	R11	2891.419	24.5	23.5	1	4	R11
3070.202	23.5	22.5	0	3	R11	2888.733	25.5	24.5	1	4	R11
3067.815	24.5	23.5	0	3	R11	2926.689	4.5	3.5	1	4	R22
3065.141	25.5	24.5	0	3	R11	2927.599	5.5	4.5	1	4	R22
3101.430	3.5	2.5	0	3	R22	2928.337	6.5	5.5	1	4	R22
3102.540	4.5	3.5	0	3	R22	2928.898	7.5	6.5	1	4	R22
3103.470	5.5	4.5	0	3	R22	2929.258	8.5	7.5	1	4	R22
3104.238	6.5	5.5	0	3	R22	2929.434	9.5	8.5	1	4	R22
3104.819	7.5	6.5	0	3	R22	2929.397	10.5	9.5	1	4	R22
3105.201	11.5	10.5	0	3	R22	2929.161	11.5	10.5	1	4	R22
3104.770	12.5	11.5	0	3	R22	2928.723	12.5	11.5	1	4	R22
3104.142	13.5	12.5	0	3	R22	2928.063	13.5	12.5	1	4	R22
3103.283	14.5	13.5	0	3	R22	2927.184	14.5	13.5	1	4	R22
3102.213	15.5	14.5	0	3	R22	2926.084	15.5	14.5	1	4	R22
3100.913	16.5	15.5	0	3	R22	2924.759	16.5	15.5	1	4	R22
3099.375	17.5	16.5	0	3	R22	2923.200	17.5	16.5	1	4	R22
3097.607	18.5	17.5	0	3	R22	2921.417	18.5	17.5	1	4	R22
3095.614	19.5	18.5	0	3	R22	2919.389	19.5	18.5	1	4	R22
3090.891	21.5	20.5	0	3	R22	2914.622	21.5	20.5	1	4	R22
3088.177	22.5	21.5	0	3	R22	2911.883	22.5	21.5	1	4	R22
2870.266	1.5	0.5	1	4	R11	2908.886	23.5	22.5	1	4	R22
2874.817	2.5	1.5	1	4	R11	2917.673	0.5	0.5	1	4	Q22
2879.011	3.5	2.5	1	4	R11	2915.611	1.5	1.5	1	4	Q22
2882.834	4.5	3.5	1	4	R11	2913.391	2.5	2.5	1	4	Q22
2886.318	5.5	4.5	1	4	R11	2911.003	3.5	3.5	1	4	Q22
2889.449	6.5	5.5	1	4	R11	2908.444	4.5	4.5	1	4	Q22
2892.234	7.5	6.5	1	4	R11	2905.718	5.5	5.5	1	4	Q22
2894.682	8.5	7.5	1	4	R11	2902.815	6.5	6.5	1	4	Q22
2896.797	9.5	8.5	1	4	R11	2899.724	7.5	7.5	1	4	Q22
2898.583	10.5	9.5	1	4	R11	2896.452	8.5	8.5	1	4	Q22
2900.047	11.5	10.5	1	4	R11	2892.986	9.5	9.5	1	4	Q22
2901.189	12.5	11.5	1	4	R11	2889.323	10.5	10.5	1	4	Q22

Table S5 (continued)

ν (cm^{-1})	J'	J''	v'	v''	branch	ν (cm^{-1})	J'	J''	v'	v''	branch
2885.460	11.5	11.5	1	4	Q22	2822.149	10.5	11.5	1	4	P11
2881.387	12.5	12.5	1	4	Q22	2816.349	11.5	12.5	1	4	P11
2877.105	13.5	13.5	1	4	Q22	2810.231	12.5	13.5	1	4	P11
2872.606	14.5	14.5	1	4	Q22	2803.804	13.5	14.5	1	4	P11
2867.889	15.5	15.5	1	4	Q22	2797.072	14.5	15.5	1	4	P11
2862.948	16.5	16.5	1	4	Q22	2790.037	15.5	16.5	1	4	P11
2857.784	17.5	17.5	1	4	Q22	2782.705	16.5	17.5	1	4	P11
2852.384	18.5	18.5	1	4	Q22	2775.081	17.5	18.5	1	4	P11
2846.759	19.5	19.5	1	4	Q22	2663.033	7.5	8.5	2	5	P11
2840.894	20.5	20.5	1	4	Q22	2658.248	8.5	9.5	2	5	P11
2834.797	21.5	21.5	1	4	Q22	2653.121	9.5	10.5	2	5	P11
2828.462	22.5	22.5	1	4	Q22	2647.685	10.5	11.5	2	5	P11
2866.627	1.5	1.5	1	4	Q11	2641.922	11.5	12.5	2	5	P11
2867.537	2.5	2.5	1	4	Q11	2635.845	12.5	13.5	2	5	P11
2868.072	3.5	3.5	1	4	Q11	2629.461	13.5	14.5	2	5	P11
2868.271	4.5	4.5	1	4	Q11	2622.774	14.5	15.5	2	5	P11
2868.116	5.5	5.5	1	4	Q11	2615.785	15.5	16.5	2	5	P11
2867.607	6.5	6.5	1	4	Q11	2608.498	16.5	17.5	2	5	P11
2866.757	7.5	7.5	1	4	Q11	2600.919	17.5	18.5	2	5	P11
2865.573	8.5	8.5	1	4	Q11	2593.048	18.5	19.5	2	5	P11
2864.058	9.5	9.5	1	4	Q11	2584.901	19.5	20.5	2	5	P11
2862.219	10.5	10.5	1	4	Q11	2576.457	20.5	21.5	2	5	P11
2860.053	11.5	11.5	1	4	Q11	2567.727	21.5	22.5	2	5	P11
2857.577	12.5	12.5	1	4	Q11	2558.730	22.5	23.5	2	5	P11
2854.789	13.5	13.5	1	4	Q11	2724.131	2.5	3.5	2	5	P22
2851.694	14.5	14.5	1	4	Q11	2718.140	3.5	4.5	2	5	P22
2848.296	15.5	15.5	1	4	Q11	2712.010	4.5	5.5	2	5	P22
2844.598	16.5	16.5	1	4	Q11	2705.700	5.5	6.5	2	5	P22
2840.605	17.5	17.5	1	4	Q11	2699.204	6.5	7.5	2	5	P22
2836.319	18.5	18.5	1	4	Q11	2692.531	7.5	8.5	2	5	P22
2831.746	19.5	19.5	1	4	Q11	2685.667	8.5	9.5	2	5	P22
2826.882	20.5	20.5	1	4	Q11	2678.619	9.5	10.5	2	5	P22
2821.736	21.5	21.5	1	4	Q11	2671.368	10.5	11.5	2	5	P22
2816.307	22.5	22.5	1	4	Q11	2663.922	11.5	12.5	2	5	P22
2810.599	23.5	23.5	1	4	Q11	2656.260	12.5	13.5	2	5	P22
2804.608	24.5	24.5	1	4	Q11	2648.398	13.5	14.5	2	5	P22
2856.590	2.5	3.5	1	4	P11	2640.315	14.5	15.5	2	5	P22
2853.502	3.5	4.5	1	4	P11	2632.017	15.5	16.5	2	5	P22
2850.058	4.5	5.5	1	4	P11	2623.497	16.5	17.5	2	5	P22
2842.106	6.5	7.5	1	4	P11	2614.756	17.5	18.5	2	5	P22
2837.617	7.5	8.5	1	4	P11	2605.786	18.5	19.5	2	5	P22
2832.790	8.5	9.5	1	4	P11	2596.591	19.5	20.5	2	5	P22
2827.635	9.5	10.5	1	4	P11	2587.162	20.5	21.5	2	5	P22

Table S5 (continued)

ν (cm^{-1})	J'	J''	v'	v''	branch	ν (cm^{-1})	J'	J''	v'	v''	branch
2695.399	1.5	0.5	2	5	R11	2730.174	24.5	23.5	2	5	R22
2704.050	3.5	2.5	2	5	R11	2740.737	1.5	1.5	2	5	Q22
2707.840	4.5	3.5	2	5	R11	2738.531	2.5	2.5	2	5	Q22
2711.278	5.5	4.5	2	5	R11	2736.156	3.5	3.5	2	5	Q22
2714.370	6.5	5.5	2	5	R11	2733.610	4.5	4.5	2	5	Q22
2717.124	7.5	6.5	2	5	R11	2730.894	5.5	5.5	2	5	Q22
2719.538	8.5	7.5	2	5	R11	2728.002	6.5	6.5	2	5	Q22
2721.621	9.5	8.5	2	5	R11	2724.925	7.5	7.5	2	5	Q22
2723.374	10.5	9.5	2	5	R11	2721.661	8.5	8.5	2	5	Q22
2724.802	11.5	10.5	2	5	R11	2718.208	9.5	9.5	2	5	Q22
2725.923	12.5	11.5	2	5	R11	2714.554	10.5	10.5	2	5	Q22
2726.727	13.5	12.5	2	5	R11	2710.701	11.5	11.5	2	5	Q22
2727.219	14.5	13.5	2	5	R11	2706.637	12.5	12.5	2	5	Q22
2727.413	15.5	14.5	2	5	R11	2702.365	13.5	13.5	2	5	Q22
2727.297	16.5	15.5	2	5	R11	2697.877	14.5	14.5	2	5	Q22
2726.887	17.5	16.5	2	5	R11	2693.166	15.5	15.5	2	5	Q22
2726.175	18.5	17.5	2	5	R11	2688.243	16.5	16.5	2	5	Q22
2725.184	19.5	18.5	2	5	R11	2683.085	17.5	17.5	2	5	Q22
2723.890	20.5	19.5	2	5	R11	2677.706	18.5	18.5	2	5	Q22
2722.317	21.5	20.5	2	5	R11	2672.090	19.5	19.5	2	5	Q22
2720.450	22.5	21.5	2	5	R11	2666.243	20.5	20.5	2	5	Q22
2718.307	23.5	22.5	2	5	R11	2660.157	21.5	21.5	2	5	Q22
2715.874	24.5	23.5	2	5	R11	2653.837	22.5	22.5	2	5	Q22
2713.156	25.5	24.5	2	5	R11	2692.679	2.5	2.5	2	5	Q11
2751.675	4.5	3.5	2	5	R22	2693.412	4.5	4.5	2	5	Q11
2752.564	5.5	4.5	2	5	R22	2692.743	6.5	6.5	2	5	Q11
2753.275	6.5	5.5	2	5	R22	2691.893	7.5	7.5	2	5	Q11
2753.809	7.5	6.5	2	5	R22	2690.710	8.5	8.5	2	5	Q11
2754.145	8.5	7.5	2	5	R22	2689.198	9.5	9.5	2	5	Q11
2754.301	9.5	8.5	2	5	R22	2687.363	10.5	10.5	2	5	Q11
2754.238	10.5	9.5	2	5	R22	2685.205	11.5	11.5	2	5	Q11
2753.980	11.5	10.5	2	5	R22	2682.732	12.5	12.5	2	5	Q11
2753.513	12.5	11.5	2	5	R22	2679.953	13.5	13.5	2	5	Q11
2752.832	13.5	12.5	2	5	R22	2676.866	14.5	14.5	2	5	Q11
2751.929	14.5	13.5	2	5	R22	2673.478	15.5	15.5	2	5	Q11
2750.802	15.5	14.5	2	5	R22	2669.790	16.5	16.5	2	5	Q11
2749.455	16.5	15.5	2	5	R22	2665.812	17.5	17.5	2	5	Q11
2747.870	17.5	16.5	2	5	R22	2661.537	18.5	18.5	2	5	Q11
2746.061	18.5	17.5	2	5	R22	2656.975	19.5	19.5	2	5	Q11
2744.017	19.5	18.5	2	5	R22	2652.126	20.5	20.5	2	5	Q11
2739.205	21.5	20.5	2	5	R22	2646.992	21.5	21.5	2	5	Q11
2736.442	22.5	21.5	2	5	R22	2641.577	22.5	22.5	2	5	Q11
2733.426	23.5	22.5	2	5	R22	2629.910	24.5	24.5	2	5	Q11

Table S5 (continued)

ν (cm^{-1})	J'	J''	v'	v''	branch	ν (cm^{-1})	J'	J''	v'	v''	branch
2493.753	6.5	7.5	3	6	P11	2528.505	13.5	13.5	3	6	Q22
2489.328	7.5	8.5	3	6	P11	2524.031	14.5	14.5	3	6	Q22
2484.576	8.5	9.5	3	6	P11	2519.338	15.5	15.5	3	6	Q22
2479.504	9.5	10.5	3	6	P11	2514.417	16.5	16.5	3	6	Q22
2474.095	10.5	11.5	3	6	P11	2509.278	17.5	17.5	3	6	Q22
2468.367	11.5	12.5	3	6	P11	2503.902	18.5	18.5	3	6	Q22
2462.339	12.5	13.5	3	6	P11	2498.299	19.5	19.5	3	6	Q22
2456.000	13.5	14.5	3	6	P11	2492.470	20.5	20.5	3	6	Q22
2449.356	14.5	15.5	3	6	P11	2486.404	21.5	21.5	3	6	Q22
2442.415	15.5	16.5	3	6	P11	2517.840	1.5	1.5	3	6	Q11
2435.175	16.5	17.5	3	6	P11	2518.717	2.5	2.5	3	6	Q11
2427.641	17.5	18.5	3	6	P11	2519.434	4.5	4.5	3	6	Q11
2419.820	18.5	19.5	3	6	P11	2519.263	5.5	5.5	3	6	Q11
2411.711	19.5	20.5	3	6	P11	2518.758	6.5	6.5	3	6	Q11
2550.283	2.5	3.5	3	6	P22	2517.908	7.5	7.5	3	6	Q11
2544.364	3.5	4.5	3	6	P22	2516.724	8.5	8.5	3	6	Q11
2538.267	4.5	5.5	3	6	P22	2515.213	9.5	9.5	3	6	Q11
2532.000	5.5	6.5	3	6	P22	2513.382	10.5	10.5	3	6	Q11
2525.552	6.5	7.5	3	6	P22	2511.230	11.5	11.5	3	6	Q11
2518.928	7.5	8.5	3	6	P22	2508.766	12.5	12.5	3	6	Q11
2512.106	8.5	9.5	3	6	P22	2505.994	13.5	13.5	3	6	Q11
2505.112	9.5	10.5	3	6	P22	2502.917	14.5	14.5	3	6	Q11
2497.907	10.5	11.5	3	6	P22	2499.543	15.5	15.5	3	6	Q11
2490.498	11.5	12.5	3	6	P22	2495.863	16.5	16.5	3	6	Q11
2482.890	12.5	13.5	3	6	P22	2491.895	17.5	17.5	3	6	Q11
2475.066	13.5	14.5	3	6	P22	2487.636	18.5	18.5	3	6	Q11
2467.029	14.5	15.5	3	6	P22	2483.089	19.5	19.5	3	6	Q11
2458.781	15.5	16.5	3	6	P22	2478.257	20.5	20.5	3	6	Q11
2450.311	16.5	17.5	3	6	P22	2473.138	21.5	21.5	3	6	Q11
2441.616	17.5	18.5	3	6	P22	2578.409	5.5	4.5	3	6	R22
2432.697	18.5	19.5	3	6	P22	2579.095	6.5	5.5	3	6	R22
2566.736	1.5	1.5	3	6	Q22	2579.601	7.5	6.5	3	6	R22
2564.547	2.5	2.5	3	6	Q22	2579.678	11.5	10.5	3	6	R22
2562.187	3.5	3.5	3	6	Q22	2579.180	12.5	11.5	3	6	R22
2559.653	4.5	4.5	3	6	Q22	2578.473	13.5	12.5	3	6	R22
2556.946	5.5	5.5	3	6	Q22	2577.544	14.5	13.5	3	6	R22
2554.068	6.5	6.5	3	6	Q22	2576.391	15.5	14.5	3	6	R22
2551.002	7.5	7.5	3	6	Q22	2575.021	16.5	15.5	3	6	R22
2547.750	8.5	8.5	3	6	Q22	2573.417	17.5	16.5	3	6	R22
2544.302	9.5	9.5	3	6	Q22	2571.585	18.5	17.5	3	6	R22
2540.662	10.5	10.5	3	6	Q22	2561.877	22.5	21.5	3	6	R22
2536.820	11.5	11.5	3	6	Q22	2558.844	23.5	22.5	3	6	R22
2532.769	12.5	12.5	3	6	Q22	2555.575	24.5	23.5	3	6	R22

Table S5 (continued)

ν (cm^{-1})	J'	J''	v'	v''	branch	ν (cm^{-1})	J'	J''	v'	v''	branch
2521.421	1.5	0.5	3	6	R11	2356.803	3.5	2.5	4	7	R11
2525.868	2.5	1.5	3	6	R11	2360.509	4.5	3.5	4	7	R11
2529.974	3.5	2.5	3	6	R11	2366.886	6.5	5.5	4	7	R11
2533.721	4.5	3.5	3	6	R11	2369.564	7.5	6.5	4	7	R11
2537.120	5.5	4.5	3	6	R11	2371.911	8.5	7.5	4	7	R11
2540.173	6.5	5.5	3	6	R11	2373.926	9.5	8.5	4	7	R11
2542.890	7.5	6.5	3	6	R11	2375.615	10.5	9.5	4	7	R11
2545.270	8.5	7.5	3	6	R11	2376.999	11.5	10.5	4	7	R11
2547.316	9.5	8.5	3	6	R11	2378.057	12.5	11.5	4	7	R11
2549.039	10.5	9.5	3	6	R11	2378.801	13.5	12.5	4	7	R11
2550.441	11.5	10.5	3	6	R11	2379.245	14.5	13.5	4	7	R11
2551.532	12.5	11.5	3	6	R11	2379.384	15.5	14.5	4	7	R11
2552.303	13.5	12.5	3	6	R11	2378.764	17.5	16.5	4	7	R11
2552.769	14.5	13.5	3	6	R11	2337.727	1.5	2.5	4	7	P11
2552.940	15.5	14.5	3	6	R11	2335.067	2.5	3.5	4	7	P11
2552.801	16.5	15.5	3	6	R11	2332.047	3.5	4.5	4	7	P11
2552.368	17.5	16.5	3	6	R11	2328.693	4.5	5.5	4	7	P11
2551.635	18.5	17.5	3	6	R11	2320.925	6.5	7.5	4	7	P11
2550.618	19.5	18.5	3	6	R11	2316.544	7.5	8.5	4	7	P11
2549.305	20.5	19.5	3	6	R11	2311.827	8.5	9.5	4	7	P11
2547.710	21.5	20.5	3	6	R11	2306.789	9.5	10.5	4	7	P11
2545.829	22.5	21.5	3	6	R11	2301.423	10.5	11.5	4	7	P11
2543.665	23.5	22.5	3	6	R11	2295.747	11.5	12.5	4	7	P11
2541.219	24.5	23.5	3	6	R11	2289.756	12.5	13.5	4	7	P11
2538.489	25.5	24.5	3	6	R11	2283.452	13.5	14.5	4	7	P11
2403.296	3.5	2.5	4	7	R22	2276.855	14.5	15.5	4	7	P11
2404.317	4.5	3.5	4	7	R22	2269.959	15.5	16.5	4	7	P11
2405.160	5.5	4.5	4	7	R22	2262.766	16.5	17.5	4	7	P11
2405.824	6.5	5.5	4	7	R22	2255.279	17.5	18.5	4	7	P11
2406.311	7.5	6.5	4	7	R22	2247.512	18.5	19.5	4	7	P11
2406.696	9.5	8.5	4	7	R22	2239.460	19.5	20.5	4	7	P11
2405.756	12.5	11.5	4	7	R22	2231.118	20.5	21.5	4	7	P11
2405.029	13.5	12.5	4	7	R22	2222.492	21.5	22.5	4	7	P11
2404.074	14.5	13.5	4	7	R22	2383.064	1.5	2.5	4	7	P22
2402.906	15.5	14.5	4	7	R22	2377.362	2.5	3.5	4	7	P22
2401.505	16.5	15.5	4	7	R22	2365.438	4.5	5.5	4	7	P22
2399.881	17.5	16.5	4	7	R22	2359.216	5.5	6.5	4	7	P22
2398.027	18.5	17.5	4	7	R22	2352.815	6.5	7.5	4	7	P22
2395.945	19.5	18.5	4	7	R22	2346.229	7.5	8.5	4	7	P22
2393.620	20.5	19.5	4	7	R22	2339.472	8.5	9.5	4	7	P22
2391.065	21.5	20.5	4	7	R22	2332.513	9.5	10.5	4	7	P22
2348.339	1.5	0.5	4	7	R11	2325.343	10.5	11.5	4	7	P22
2352.746	2.5	1.5	4	7	R11	2317.989	11.5	12.5	4	7	P22

Table S5 (continued)

ν (cm^{-1})	J'	J''	v'	v''	branch	ν (cm^{-1})	J'	J''	v'	v''	branch
2310.430	12.5	13.5	4	7	P22	2335.717	12.5	12.5	4	7	Q11
2294.670	14.5	15.5	4	7	P22	2332.952	13.5	13.5	4	7	Q11
2286.468	15.5	16.5	4	7	P22	2329.891	14.5	14.5	4	7	Q11
2278.038	16.5	17.5	4	7	P22	2326.511	15.5	15.5	4	7	Q11
2269.391	17.5	18.5	4	7	P22	2322.851	16.5	16.5	4	7	Q11
2260.520	18.5	19.5	4	7	P22	2318.895	17.5	17.5	4	7	Q11
2251.424	19.5	20.5	4	7	P22	2314.651	18.5	18.5	4	7	Q11
2242.092	20.5	21.5	4	7	P22	2310.120	19.5	19.5	4	7	Q11
2232.534	21.5	22.5	4	7	P22	2305.301	20.5	20.5	4	7	Q11
2222.741	22.5	23.5	4	7	P22	2300.203	21.5	21.5	4	7	Q11
2395.664	0.5	0.5	4	7	Q22	2294.823	22.5	22.5	4	7	Q11
2393.656	1.5	1.5	4	7	Q22	2289.164	23.5	23.5	4	7	Q11
2391.475	2.5	2.5	4	7	Q22	2283.229	24.5	24.5	4	7	Q11
2389.130	3.5	3.5	4	7	Q22	2156.743	4.5	5.5	5	8	P11
2386.610	4.5	4.5	4	7	Q22	2153.064	5.5	6.5	5	8	P11
2383.917	5.5	5.5	4	7	Q22	2149.053	6.5	7.5	5	8	P11
2381.051	6.5	6.5	4	7	Q22	2144.700	7.5	8.5	5	8	P11
2377.992	7.5	7.5	4	7	Q22	2140.026	8.5	9.5	5	8	P11
2371.320	9.5	9.5	4	7	Q22	2135.024	9.5	10.5	5	8	P11
2367.689	10.5	10.5	4	7	Q22	2129.698	10.5	11.5	5	8	P11
2363.853	11.5	11.5	4	7	Q22	2124.061	11.5	12.5	5	8	P11
2359.816	12.5	12.5	4	7	Q22	2118.117	12.5	13.5	5	8	P11
2355.561	13.5	13.5	4	7	Q22	2111.870	13.5	14.5	5	8	P11
2351.097	14.5	14.5	4	7	Q22	2105.308	14.5	15.5	5	8	P11
2346.413	15.5	15.5	4	7	Q22	2098.461	15.5	16.5	5	8	P11
2341.507	16.5	16.5	4	7	Q22	2091.315	16.5	17.5	5	8	P11
2336.383	17.5	17.5	4	7	Q22	2083.882	17.5	18.5	5	8	P11
2331.027	18.5	18.5	4	7	Q22	2076.163	18.5	19.5	5	8	P11
2325.431	19.5	19.5	4	7	Q22	2068.153	19.5	20.5	5	8	P11
2319.617	20.5	20.5	4	7	Q22	2059.869	20.5	21.5	5	8	P11
2313.563	21.5	21.5	4	7	Q22	2051.295	21.5	22.5	5	8	P11
2307.276	22.5	22.5	4	7	Q22	2211.045	1.5	2.5	5	8	P22
2344.801	1.5	1.5	4	7	Q11	2205.373	2.5	3.5	5	8	P22
2345.681	2.5	2.5	4	7	Q11	2199.561	3.5	4.5	5	8	P22
2346.192	3.5	3.5	4	7	Q11	2193.557	4.5	5.5	5	8	P22
2346.368	4.5	4.5	4	7	Q11	2187.388	5.5	6.5	5	8	P22
2346.192	5.5	5.5	4	7	Q11	2181.035	6.5	7.5	5	8	P22
2345.681	6.5	6.5	4	7	Q11	2174.508	7.5	8.5	5	8	P22
2344.834	7.5	7.5	4	7	Q11	2167.781	8.5	9.5	5	8	P22
2343.651	8.5	8.5	4	7	Q11	2160.862	9.5	10.5	5	8	P22
2342.145	9.5	9.5	4	7	Q11	2153.754	10.5	11.5	5	8	P22
2340.318	10.5	10.5	4	7	Q11	2146.444	11.5	12.5	5	8	P22
2338.173	11.5	11.5	4	7	Q11	2138.926	12.5	13.5	5	8	P22

Table S5 (continued)

ν (cm^{-1})	J'	J''	v'	v''	branch	ν (cm^{-1})	J'	J''	v'	v''	branch
2131.205	13.5	14.5	5	8	P22	2191.840	11.5	11.5	5	8	Q22
2123.264	14.5	15.5	5	8	P22	2187.813	12.5	12.5	5	8	Q22
2115.105	15.5	16.5	5	8	P22	2183.576	13.5	13.5	5	8	Q22
2106.730	16.5	17.5	5	8	P22	2179.122	14.5	14.5	5	8	Q22
2098.133	17.5	18.5	5	8	P22	2174.454	15.5	15.5	5	8	Q22
2089.309	18.5	19.5	5	8	P22	2169.559	16.5	16.5	5	8	Q22
2080.259	19.5	20.5	5	8	P22	2164.446	17.5	17.5	5	8	Q22
2070.983	20.5	21.5	5	8	P22	2159.104	18.5	18.5	5	8	Q22
2061.482	21.5	22.5	5	8	P22	2153.528	19.5	19.5	5	8	Q22
2172.704	1.5	1.5	5	8	Q11	2147.727	20.5	20.5	5	8	Q22
2174.069	3.5	3.5	5	8	Q11	2141.690	21.5	21.5	5	8	Q22
2174.246	4.5	4.5	5	8	Q11	2135.415	22.5	22.5	5	8	Q22
2174.069	5.5	5.5	5	8	Q11	2176.207	1.5	0.5	5	8	R11
2173.555	6.5	6.5	5	8	Q11	2180.573	2.5	1.5	5	8	R11
2172.704	7.5	7.5	5	8	Q11	2184.579	3.5	2.5	5	8	R11
2171.527	8.5	8.5	5	8	Q11	2188.246	4.5	3.5	5	8	R11
2170.024	9.5	9.5	5	8	Q11	2191.563	5.5	4.5	5	8	R11
2168.201	10.5	10.5	5	8	Q11	2194.546	6.5	5.5	5	8	R11
2166.062	11.5	11.5	5	8	Q11	2197.180	7.5	6.5	5	8	R11
2163.616	12.5	12.5	5	8	Q11	2199.499	8.5	7.5	5	8	R11
2160.862	13.5	13.5	5	8	Q11	2201.483	9.5	8.5	5	8	R11
2157.805	14.5	14.5	5	8	Q11	2203.144	10.5	9.5	5	8	R11
2154.447	15.5	15.5	5	8	Q11	2204.488	11.5	10.5	5	8	R11
2150.796	16.5	16.5	5	8	Q11	2205.521	12.5	11.5	5	8	R11
2146.854	17.5	17.5	5	8	Q11	2206.243	13.5	12.5	5	8	R11
2142.624	18.5	18.5	5	8	Q11	2206.661	14.5	13.5	5	8	R11
2138.108	19.5	19.5	5	8	Q11	2206.775	15.5	14.5	5	8	R11
2133.306	20.5	20.5	5	8	Q11	2206.589	16.5	15.5	5	8	R11
2128.224	21.5	21.5	5	8	Q11	2206.114	17.5	16.5	5	8	R11
2122.861	22.5	22.5	5	8	Q11	2205.337	18.5	17.5	5	8	R11
2117.225	23.5	23.5	5	8	Q11	2204.277	19.5	18.5	5	8	R11
2111.305	24.5	24.5	5	8	Q11	2202.929	20.5	19.5	5	8	R11
2223.514	0.5	0.5	5	8	Q22	2201.293	21.5	20.5	5	8	R11
2221.516	1.5	1.5	5	8	Q22	2194.696	24.5	23.5	5	8	R11
2219.360	2.5	2.5	5	8	Q22	2191.935	25.5	24.5	5	8	R11
2217.022	3.5	3.5	5	8	Q22	2232.048	4.5	3.5	5	8	R22
2214.518	4.5	4.5	5	8	Q22	2233.504	6.5	5.5	5	8	R22
2211.838	5.5	5.5	5	8	Q22	2233.964	7.5	6.5	5	8	R22
2208.980	6.5	6.5	5	8	Q22	2234.238	8.5	7.5	5	8	R22
2205.938	7.5	7.5	5	8	Q22	2234.304	9.5	8.5	5	8	R22
2202.707	8.5	8.5	5	8	Q22	2234.173	10.5	9.5	5	8	R22
2199.284	9.5	9.5	5	8	Q22	2233.837	11.5	10.5	5	8	R22
2195.663	10.5	10.5	5	8	Q22	2233.297	12.5	11.5	5	8	R22

Table S5 (continued)

ν (cm^{-1})	J'	J''	v'	v''	branch	ν (cm^{-1})	J'	J''	v'	v''	branch
2232.534	13.5	12.5	5	8	R22	1986.723	14.5	14.5	6	9	Q11
2231.564	14.5	13.5	5	8	R22	1983.379	15.5	15.5	6	9	Q11
2230.371	15.5	14.5	5	8	R22	1979.748	16.5	16.5	6	9	Q11
2228.946	16.5	15.5	5	8	R22	1975.822	17.5	17.5	6	9	Q11
2227.298	17.5	16.5	5	8	R22	1971.613	18.5	18.5	6	9	Q11
2225.421	18.5	17.5	5	8	R22	1967.118	19.5	19.5	6	9	Q11
2223.305	19.5	18.5	5	8	R22	1962.337	20.5	20.5	6	9	Q11
2218.383	21.5	20.5	5	8	R22	1989.108	3.5	3.5	6	9	Q12
2215.552	22.5	21.5	5	8	R22	1985.816	4.5	4.5	6	9	Q12
1973.846	7.5	8.5	6	9	P11	1982.182	5.5	5.5	6	9	Q12
1969.200	8.5	9.5	6	9	P11	1978.202	6.5	6.5	6	9	Q12
1964.247	9.5	10.5	6	9	P11	2060.744	4.5	4.5	6	9	Q12
1958.962	10.5	11.5	6	9	P11	2061.542	5.5	5.5	6	9	Q12
1953.375	11.5	12.5	6	9	P11	2062.165	6.5	6.5	6	9	Q12
1947.476	12.5	13.5	6	9	P11	2062.587	7.5	7.5	6	9	Q12
1941.274	13.5	14.5	6	9	P11	2062.868	9.5	9.5	6	9	Q12
1927.962	15.5	16.5	6	9	P11	2062.704	10.5	10.5	6	9	Q12
1920.865	16.5	17.5	6	9	P11	2043.434	4.5	4.5	6	9	Q12
1905.822	18.5	19.5	6	9	P11	2040.765	5.5	5.5	6	9	Q12
1978.202	2.5	3.5	6	9	P12	2037.913	6.5	6.5	6	9	Q12
2040.729	5.5	6.5	6	9	P21	2034.894	7.5	7.5	6	9	Q12
2037.874	6.5	7.5	6	9	P21	2031.666	8.5	8.5	6	9	Q12
2034.405	2.5	3.5	6	9	P22	2028.249	9.5	9.5	6	9	Q12
2022.692	4.5	5.5	6	9	P22	2024.651	10.5	10.5	6	9	Q12
2003.776	7.5	8.5	6	9	P22	2020.845	11.5	11.5	6	9	Q12
1997.115	8.5	9.5	6	9	P22	2016.824	12.5	12.5	6	9	Q12
1990.235	9.5	10.5	6	9	P22	2012.598	13.5	13.5	6	9	Q12
1983.173	10.5	11.5	6	9	P22	2008.161	14.5	14.5	6	9	Q12
1975.909	11.5	12.5	6	9	P22	2003.503	15.5	15.5	6	9	Q12
1968.441	12.5	13.5	6	9	P22	1998.624	16.5	16.5	6	9	Q12
1944.761	15.5	16.5	6	9	P22	1993.523	17.5	17.5	6	9	Q12
1936.431	16.5	17.5	6	9	P22	1982.641	19.5	19.5	6	9	Q12
2002.445	2.5	2.5	6	9	Q11	1964.587	22.5	22.5	6	9	Q12
2003.103	4.5	4.5	6	9	Q11	2020.243	5.5	4.5	6	9	Q12
2002.931	5.5	5.5	6	9	Q11	2025.793	7.5	6.5	6	9	Q12
2002.410	6.5	6.5	6	9	Q11	2028.071	8.5	7.5	6	9	Q12
2001.561	7.5	7.5	6	9	Q11	2030.025	9.5	8.5	6	9	Q12
2000.388	8.5	8.5	6	9	Q11	2031.666	10.5	9.5	6	9	Q12
1998.893	9.5	9.5	6	9	Q11	2033.982	12.5	11.5	6	9	Q12
1997.079	10.5	10.5	6	9	Q11	2034.675	13.5	12.5	6	9	Q12
1994.952	11.5	11.5	6	9	Q11	2035.156	15.5	14.5	6	9	Q12
1992.507	12.5	12.5	6	9	Q11	2034.962	16.5	15.5	6	9	Q12
1989.766	13.5	13.5	6	9	Q11	2034.459	17.5	16.5	6	9	Q12

Table S5 (continued)

ν (cm^{-1})	J'	J''	v'	v''	branch
2033.667	18.5	17.5	6	9	R11
2032.593	19.5	18.5	6	9	R11
2027.638	22.5	21.5	6	9	R11
2002.445	2.5	1.5	6	9	R12
2002.445	6.5	5.5	6	9	R12
2000.434	8.5	7.5	6	9	R12
1998.940	9.5	8.5	6	9	R12
1995.014	11.5	10.5	6	9	R12
2062.200	6.5	5.5	6	9	R22
2062.632	7.5	6.5	6	9	R22
2062.868	8.5	7.5	6	9	R22
2062.920	9.5	8.5	6	9	R22
2062.766	10.5	9.5	6	9	R22
2062.409	11.5	10.5	6	9	R22
2061.837	12.5	11.5	6	9	R22
2060.065	14.5	13.5	6	9	R22

Table S6: Emission lines of the CN radical for $A^2\Pi \rightarrow X^2\Sigma^+$ transitions and Δv = -2

**ν (cm^{-1})**	**J'**	**J''**	**v'**	**v''**	**ν (cm^{-1})**	**J'**	**J''**	**v'**	**v''**
4187.060	55.5	56.5	0	2	4836.980	27.5	28.5	0	2
4384.736	46.5	47.5	0	2	4838.876	19.5	20.5	0	2
4401.342	54.5	55.5	0	2	4842.401	37.5	37.5	0	2
4419.728	65.5	65.5	0	2	4844.780	26.5	27.5	0	2
4427.216	64.5	64.5	0	2	4849.194	26.5	27.5	0	2
4440.598	52.5	53.5	0	2	4851.492	18.5	19.5	0	2
4459.823	51.5	52.5	0	2	4867.243	49.5	48.5	0	2
4465.156	62.5	62.5	0	2	4874.208	34.5	33.5	0	2
4497.418	49.5	50.5	0	2	4876.348	34.5	34.5	0	2
4506.292	49.5	49.5	0	2	4877.305	23.5	24.5	0	2
4515.793	48.5	49.5	0	2	4878.133	48.5	47.5	0	2
4529.501	59.5	59.5	0	2	4896.900	32.5	33.5	0	2
4541.865	47.5	47.5	0	2	4897.432	21.5	22.5	0	2
4551.711	46.5	47.5	0	2	4898.701	14.5	15.5	0	2
4575.975	45.5	46.5	0	2	4898.884	46.5	45.5	0	2
4580.511	56.5	56.5	0	2	4906.127	21.5	22.5	0	2
4588.570	55.5	56.5	0	2	4909.341	45.5	44.5	0	2
4603.495	43.5	44.5	0	2	4916.693	20.5	21.5	0	2
4609.256	43.5	44.5	0	2	4919.501	44.5	43.5	0	2
4613.054	54.5	54.5	0	2	4925.252	18.5	19.5	0	2
4652.749	40.5	41.5	0	2	4925.395	18.5	18.5	0	2
4653.081	51.5	52.5	0	2	4926.986	19.5	20.5	0	2
4660.060	51.5	50.5	0	2	4930.614	11.5	12.5	0	2
4674.663	50.5	50.5	0	2	4933.883	17.5	18.5	0	2
4687.681	38.5	38.5	0	2	4934.024	17.5	17.5	0	2
4702.155	37.5	38.5	0	2	4935.077	28.5	29.5	0	2
4713.013	47.5	48.5	0	2	4937.016	27.5	27.5	0	2
4713.263	61.5	60.5	0	2	4940.562	10.5	11.5	0	2
4731.022	35.5	35.5	0	2	4942.315	16.5	16.5	0	2
4741.910	59.5	58.5	0	2	4948.218	41.5	40.5	0	2
4750.464	58.5	57.5	0	2	4950.157	9.5	10.5	0	2
4755.790	58.5	57.5	0	2	4950.288	15.5	15.5	0	2
4771.727	32.5	33.5	0	2	4956.287	16.5	17.5	0	2
4778.144	56.5	55.5	0	2	4957.919	14.5	14.5	0	2
4784.672	31.5	31.5	0	2	4959.394	8.5	9.5	0	2
4785.294	23.5	24.5	0	2	4959.599	24.5	23.5	0	2
4797.111	30.5	31.5	0	2	4960.736	25.5	26.5	0	2
4808.131	40.5	40.5	0	2	4965.530	15.5	16.5	0	2
4819.100	39.5	40.5	0	2	4970.334	38.5	37.5	0	2
4829.606	52.5	52.5	0	2	4974.516	14.5	15.5	0	2
4831.355	38.5	38.5	0	2	4976.765	6.5	7.5	0	2
4832.786	52.5	51.5	0	2	4979.018	21.5	21.5	0	2
4833.317	27.5	28.5	0	2	4984.054	22.5	22.5	0	2

Table S6 (continued)

ν (cm^{-1})	J'	J''	v'	v''	ν (cm^{-1})	J'	J''	v'	v''
4984.945	10.5	11.5	0	2	5043.585	12.5	12.5	0	2
4985.032	10.5	10.5	0	2	5044.416	25.5	24.5	0	2
4990.928	9.5	9.5	0	2	5044.497	3.5	2.5	0	2
4991.055	21.5	22.5	0	2	5047.193	27.5	27.5	0	2
4991.215	21.5	21.5	0	2	5048.032	11.5	12.5	0	2
4991.734	12.5	13.5	0	2	5048.265	4.5	3.5	0	2
4992.631	4.5	5.5	0	2	5051.158	4.5	5.5	0	2
4995.880	18.5	17.5	0	2	5052.114	26.5	26.5	0	2
4996.471	8.5	8.5	0	2	5052.417	10.5	10.5	0	2
4998.090	20.5	20.5	0	2	5054.002	22.5	21.5	0	2
4998.685	34.5	33.5	0	2	5056.475	9.5	9.5	0	2
4999.972	11.5	12.5	0	2	5056.558	21.5	20.5	0	2
5000.673	17.5	17.5	0	2	5056.921	25.5	24.5	0	2
5000.808	17.5	16.5	0	2	5057.603	3.5	4.5	0	2
5001.497	54.5	53.5	0	2	5060.302	8.5	8.5	0	2
5001.644	7.5	7.5	0	2	5060.710	19.5	18.5	0	2
5004.703	34.5	34.5	0	2	5061.265	24.5	23.5	0	2
5006.952	2.5	3.5	0	2	5061.349	9.5	8.5	0	2
5007.968	10.5	11.5	0	2	5063.903	7.5	7.5	0	2
5010.860	5.5	5.5	0	2	5063.996	11.5	10.5	0	2
5011.045	18.5	18.5	0	2	5064.471	16.5	15.5	0	2
5014.889	4.5	4.5	0	2	5065.222	13.5	12.5	0	2
5015.726	9.5	10.5	0	2	5065.313	14.5	13.5	0	2
5016.987	17.5	18.5	0	2	5067.281	6.5	6.5	0	2
5018.297	32.5	32.5	0	2	5068.931	22.5	22.5	0	2
5018.528	3.5	3.5	0	2	5070.440	5.5	5.5	0	2
5021.778	2.5	3.5	0	2	5073.391	4.5	4.5	0	2
5022.122	30.5	29.5	0	2	5075.805	20.5	19.5	0	2
5023.232	11.5	11.5	0	2	5076.131	3.5	3.5	0	2
5023.320	11.5	10.5	0	2	5078.667	2.5	3.5	0	2
5024.640	1.5	2.5	0	2	5078.747	19.5	18.5	0	2
5025.861	10.5	9.5	0	2	5081.012	1.5	2.5	0	2
5028.055	9.5	8.5	0	2	5081.284	18.5	18.5	0	2
5028.476	15.5	15.5	0	2	5081.415	18.5	17.5	0	2
5030.721	30.5	30.5	0	2	5086.951	0.5	0.5	0	2
5033.427	4.5	4.5	0	2	5088.442	1.5	1.5	0	2
5033.655	14.5	15.5	0	2	5090.987	3.5	3.5	0	2
5033.772	14.5	14.5	0	2	5091.956	4.5	4.5	0	2
5036.439	27.5	26.5	0	2	5092.746	5.5	4.5	0	2
5037.637	6.5	7.5	0	2	5093.553	9.5	9.5	0	2
5038.801	13.5	13.5	0	2	5093.625	9.5	8.5	0	2
5040.331	2.5	1.5	0	2	5093.689	8.5	8.5	0	2
5040.584	26.5	25.5	0	2	5102.092	3.5	2.5	0	2

Table S6 (continued)

ν (cm^{-1})	J'	J''	v'	v''	ν (cm^{-1})	J'	J''	v'	v''
5106.763	4.5	3.5	0	2	4777.023	11.5	12.5	1	3
5109.231	39.5	38.5	0	2	4777.112	11.5	11.5	1	3
5115.479	6.5	5.5	0	2	4782.094	36.5	35.5	1	3
5119.514	7.5	6.5	0	2	4783.251	10.5	11.5	1	3
5123.317	8.5	7.5	0	2	4783.333	10.5	10.5	1	3
5126.906	9.5	8.5	0	2	4789.194	9.5	9.5	1	3
5130.243	10.5	9.5	0	2	4789.319	21.5	21.5	1	3
5133.351	11.5	10.5	0	2	4794.699	8.5	8.5	1	3
5136.436	32.5	31.5	0	2	4798.639	17.5	17.5	1	3
5145.086	16.5	15.5	0	2	4802.653	19.5	20.5	1	3
5149.717	20.5	19.5	0	2	4803.231	16.5	16.5	1	3
5150.273	23.5	22.5	0	2	4808.067	32.5	31.5	1	3
4463.941	31.5	32.5	1	3	4808.990	18.5	19.5	1	3
4468.423	39.5	40.5	1	3	4809.123	18.5	18.5	1	3
4501.748	37.5	38.5	1	3	4811.418	14.5	14.5	1	3
4528.821	35.5	36.5	1	3	4812.993	4.5	4.5	1	3
4544.316	34.5	34.5	1	3	4814.110	9.5	10.5	1	3
4557.584	33.5	34.5	1	3	4815.190	17.5	17.5	1	3
4557.829	33.5	33.5	1	3	4816.603	3.5	3.5	1	3
4570.799	32.5	33.5	1	3	4818.253	12.5	12.5	1	3
4596.332	30.5	31.5	1	3	4818.342	12.5	11.5	1	3
4625.454	20.5	21.5	1	3	4819.829	2.5	2.5	1	3
4629.725	38.5	38.5	1	3	4821.156	11.5	11.5	1	3
4648.346	26.5	27.5	1	3	4821.238	11.5	10.5	1	3
4651.467	36.5	36.5	1	3	4821.601	8.5	9.5	1	3
4663.035	17.5	18.5	1	3	4822.665	1.5	2.5	1	3
4674.928	16.5	17.5	1	3	4823.704	10.5	10.5	1	3
4691.673	32.5	32.5	1	3	4823.784	10.5	9.5	1	3
4705.038	21.5	22.5	1	3	4825.895	9.5	9.5	1	3
4708.629	13.5	14.5	1	3	4826.526	15.5	15.5	1	3
4709.959	30.5	30.5	1	3	4830.850	2.5	2.5	1	3
4710.176	30.5	29.5	1	3	4831.288	3.5	3.5	1	3
4714.988	19.5	20.5	1	3	4831.811	14.5	14.5	1	3
4715.138	19.5	19.5	1	3	4833.693	1.5	0.5	1	3
4715.555	20.5	21.5	1	3	4835.891	6.5	7.5	1	3
4740.869	16.5	16.5	1	3	4836.833	13.5	13.5	1	3
4754.974	16.5	17.5	1	3	4837.741	26.5	25.5	1	3
4757.470	24.5	24.5	1	3	4838.203	2.5	1.5	1	3
4764.310	23.5	23.5	1	3	4839.161	28.5	28.5	1	3
4767.051	24.5	24.5	1	3	4841.608	12.5	12.5	1	3
4770.825	22.5	22.5	1	3	4842.320	3.5	2.5	1	3
4773.116	14.5	15.5	1	3	4842.709	5.5	6.5	1	3
4775.207	6.5	7.5	1	3	4844.389	27.5	27.5	1	3

Table S6 (continued)

ν (cm^{-1})	J'	J''	v'	v''	ν (cm^{-1})	J'	J''	v'	v''
4849.313	4.5	5.5	1	3	4566.094	7.5	8.5	2	4
4858.233	8.5	9.5	1	3	4569.641	12.5	13.5	2	4
4861.540	11.5	10.5	1	3	4569.735	12.5	12.5	2	4
4861.883	7.5	7.5	1	3	4572.521	14.5	15.5	2	4
4862.300	12.5	11.5	1	3	4573.525	23.5	24.5	2	4
4862.488	15.5	14.5	1	3	4576.258	11.5	11.5	2	4
4862.702	13.5	12.5	1	3	4581.167	13.5	14.5	2	4
4865.255	6.5	6.5	1	3	4581.715	20.5	20.5	2	4
4866.400	22.5	21.5	1	3	4581.855	20.5	19.5	2	4
4868.403	5.5	5.5	1	3	4582.441	10.5	10.5	2	4
4869.923	21.5	20.5	1	3	4588.266	9.5	9.5	2	4
4871.339	4.5	5.5	1	3	4589.567	12.5	13.5	2	4
4873.161	20.5	19.5	1	3	4592.637	18.5	17.5	2	4
4876.125	19.5	18.5	1	3	4593.738	8.5	8.5	2	4
4881.124	17.5	17.5	1	3	4597.279	3.5	4.5	2	4
4881.242	17.5	16.5	1	3	4597.534	17.5	16.5	2	4
4886.281	1.5	0.5	1	3	4598.837	7.5	7.5	2	4
4888.781	3.5	3.5	1	3	4601.541	19.5	20.5	2	4
4891.024	6.5	5.5	1	3	4603.567	6.5	6.5	2	4
4891.268	9.5	8.5	1	3	4604.131	2.5	3.5	2	4
4891.420	8.5	7.5	1	3	4607.923	5.5	5.5	2	4
4904.836	39.5	38.5	1	3	4610.162	14.5	14.5	2	4
4927.519	10.5	9.5	1	3	4611.898	4.5	4.5	2	4
4933.352	12.5	11.5	1	3	4618.670	2.5	3.5	2	4
4946.784	21.5	20.5	1	3	4619.965	11.5	10.5	2	4
4412.692	21.5	22.5	2	4	4621.487	1.5	2.5	2	4
4463.117	17.5	18.5	2	4	4622.503	10.5	9.5	2	4
4465.421	24.5	25.5	2	4	4624.691	9.5	8.5	2	4
4485.982	22.5	23.5	2	4	4625.376	15.5	15.5	2	4
4486.150	22.5	22.5	2	4	4627.972	7.5	6.5	2	4
4494.012	22.5	23.5	2	4	4630.074	4.5	4.5	2	4
4504.753	21.5	22.5	2	4	4632.417	1.5	0.5	2	4
4505.297	20.5	21.5	2	4	4635.667	13.5	13.5	2	4
4514.488	19.5	20.5	2	4	4639.581	25.5	24.5	2	4
4523.344	18.5	19.5	2	4	4640.346	12.5	13.5	2	4
4525.444	19.5	20.5	2	4	4641.716	5.5	6.5	2	4
4532.014	17.5	17.5	2	4	4644.868	11.5	12.5	2	4
4535.375	18.5	19.5	2	4	4646.382	23.5	22.5	2	4
4540.220	16.5	16.5	2	4	4651.907	21.5	20.5	2	4
4548.107	15.5	15.5	2	4	4652.032	25.5	25.5	2	4
4548.274	9.5	10.5	2	4	4653.393	7.5	6.5	2	4
4555.659	14.5	14.5	2	4	4654.186	20.5	19.5	2	4
4563.144	23.5	23.5	2	4	4655.555	8.5	7.5	2	4

Table S6 (continued)

ν (cm^{-1})	J'	J''	v'	v''	ν (cm^{-1})	J'	J''	v'	v''
4656.154	19.5	18.5	2	4	4249.108	26.5	27.5	3	5
4657.356	9.5	8.5	2	4	4264.027	17.5	18.5	3	5
4660.663	7.5	7.5	2	4	4266.166	24.5	24.5	3	5
4660.791	2.5	3.5	2	4	4276.390	23.5	24.5	3	5
4664.020	6.5	6.5	2	4	4281.154	33.5	32.5	3	5
4666.732	1.5	2.5	2	4	4283.643	23.5	24.5	3	5
4667.161	5.5	5.5	2	4	4284.192	33.5	33.5	3	5
4667.906	21.5	21.5	2	4	4286.461	22.5	23.5	3	5
4670.090	4.5	4.5	2	4	4304.079	31.5	32.5	3	5
4671.321	20.5	19.5	2	4	4305.687	20.5	21.5	3	5
4674.179	19.5	19.5	2	4	4308.699	30.5	30.5	3	5
4674.313	19.5	18.5	2	4	4308.973	13.5	14.5	3	5
4675.315	2.5	3.5	2	4	4312.701	44.5	43.5	3	5
4683.576	15.5	14.5	2	4	4313.928	30.5	30.5	3	5
4685.231	14.5	13.5	2	4	4314.952	19.5	19.5	3	5
4686.534	13.5	13.5	2	4	4315.752	20.5	21.5	3	5
4688.298	4.5	4.5	2	4	4323.629	18.5	19.5	3	5
4689.544	6.5	5.5	2	4	4323.762	18.5	18.5	3	5
4689.830	8.5	8.5	2	4	4329.465	11.5	12.5	3	5
4707.182	5.5	4.5	2	4	4330.240	42.5	41.5	3	5
4725.585	10.5	9.5	2	4	4332.124	17.5	18.5	3	5
3995.269	44.5	45.5	3	5	4332.256	17.5	17.5	3	5
4011.897	43.5	43.5	3	5	4333.748	27.5	27.5	3	5
4034.413	33.5	34.5	3	5	4335.803	18.5	19.5	3	5
4067.299	31.5	32.5	3	5	4340.297	16.5	17.5	3	5
4070.575	39.5	40.5	3	5	4340.929	27.5	28.5	3	5
4083.304	30.5	31.5	3	5	4345.437	17.5	18.5	3	5
4089.126	38.5	38.5	3	5	4348.138	15.5	16.5	3	5
4101.107	37.5	38.5	3	5	4348.903	25.5	25.5	3	5
4103.697	37.5	37.5	3	5	4350.559	40.5	39.5	3	5
4114.433	28.5	29.5	3	5	4355.651	14.5	15.5	3	5
4130.524	35.5	36.5	3	5	4357.851	25.5	25.5	3	5
4142.945	45.5	45.5	3	5	4362.831	13.5	14.5	3	5
4145.431	34.5	35.5	3	5	4363.906	15.5	16.5	3	5
4145.671	34.5	34.5	3	5	4365.796	24.5	24.5	3	5
4169.189	43.5	42.5	3	5	4366.243	7.5	8.5	3	5
4179.652	42.5	42.5	3	5	4369.662	12.5	13.5	3	5
4209.480	29.5	30.5	3	5	4372.761	14.5	15.5	3	5
4216.792	39.5	40.5	3	5	4375.498	21.5	21.5	3	5
4217.058	39.5	39.5	3	5	4375.640	21.5	20.5	3	5
4224.704	28.5	29.5	3	5	4376.158	11.5	12.5	3	5
4228.628	38.5	38.5	3	5	4380.861	22.5	22.5	3	5
4240.324	37.5	38.5	3	5	4381.357	20.5	20.5	3	5

Table S6 (continued)

ν (cm^{-1})	J'	J''	v'	v''	ν (cm^{-1})	J'	J''	v'	v''
4381.498	20.5	19.5	3	5	4435.333	13.5	13.5	3	5
4382.300	10.5	11.5	3	5	4436.385	2.5	1.5	3	5
4382.381	10.5	10.5	3	5	4440.413	3.5	2.5	3	5
4383.487	36.5	36.5	3	5	4441.428	27.5	26.5	3	5
4387.032	19.5	18.5	3	5	4444.518	11.5	12.5	3	5
4388.100	9.5	10.5	3	5	4444.602	11.5	11.5	3	5
4389.718	12.5	13.5	3	5	4445.242	23.5	22.5	3	5
4390.009	4.5	5.5	3	5	4447.313	5.5	4.5	3	5
4392.121	18.5	18.5	3	5	4448.192	22.5	21.5	3	5
4393.600	8.5	8.5	3	5	4450.190	6.5	5.5	3	5
4394.837	20.5	20.5	3	5	4450.815	21.5	20.5	3	5
4397.022	17.5	17.5	3	5	4450.908	25.5	25.5	3	5
4397.822	11.5	12.5	3	5	4452.906	9.5	9.5	3	5
4398.670	7.5	7.5	3	5	4455.102	19.5	18.5	3	5
4401.273	19.5	20.5	3	5	4456.636	8.5	9.5	3	5
4401.413	19.5	19.5	3	5	4456.701	8.5	8.5	3	5
4401.595	16.5	16.5	3	5	4457.991	10.5	9.5	3	5
4403.369	6.5	6.5	3	5	4458.096	17.5	16.5	3	5
4403.957	2.5	3.5	3	5	4459.747	12.5	11.5	3	5
4405.560	33.5	32.5	3	5	4460.089	13.5	12.5	3	5
4405.838	15.5	15.5	3	5	4460.281	7.5	7.5	3	5
4409.746	14.5	14.5	3	5	4463.631	6.5	6.5	3	5
4411.634	4.5	4.5	3	5	4466.382	1.5	2.5	3	5
4413.319	13.5	13.5	3	5	4469.667	4.5	4.5	3	5
4413.769	17.5	17.5	3	5	4472.376	3.5	3.5	3	5
4415.163	3.5	4.5	3	5	4474.869	2.5	3.5	3	5
4416.551	12.5	12.5	3	5	4475.859	43.5	42.5	3	5
4418.369	2.5	2.5	3	5	4477.176	1.5	2.5	3	5
4419.544	16.5	16.5	3	5	4480.756	16.5	15.5	3	5
4420.716	8.5	9.5	3	5	4481.980	42.5	41.5	3	5
4421.155	1.5	1.5	3	5	4482.696	15.5	14.5	3	5
4421.980	10.5	10.5	3	5	4484.375	14.5	13.5	3	5
4422.053	10.5	9.5	3	5	4485.796	13.5	12.5	3	5
4424.238	9.5	8.5	3	5	4486.809	3.5	3.5	3	5
4424.786	30.5	29.5	3	5	4486.885	12.5	12.5	3	5
4425.784	28.5	27.5	3	5	4487.807	11.5	11.5	3	5
4427.882	7.5	8.5	3	5	4487.989	1.5	0.5	3	5
4428.362	1.5	0.5	3	5	4488.486	10.5	10.5	3	5
4429.176	2.5	2.5	3	5	4488.558	10.5	9.5	3	5
4429.293	5.5	5.5	3	5	4488.908	6.5	5.5	3	5
4429.617	3.5	2.5	3	5	4488.991	9.5	8.5	3	5
4430.329	14.5	14.5	3	5	4489.187	8.5	7.5	3	5
4434.826	6.5	7.5	3	5	4497.601	3.5	2.5	3	5

Table S6 (continued)

ν (cm^{-1})	J'	J''	v'	v''	ν (cm^{-1})	J'	J''	v'	v''
4508.112	37.5	36.5	3	5	4163.858	13.5	13.5	4	6
4517.937	8.5	7.5	3	5	4165.068	15.5	16.5	4	6
4534.665	14.5	13.5	3	5	4166.465	24.5	24.5	4	6
4541.567	19.5	18.5	3	5	4167.013	38.5	38.5	4	6
3994.603	41.5	41.5	4	6	4167.255	7.5	8.5	4	6
3994.882	41.5	40.5	4	6	4169.860	22.5	22.5	4	6
3997.024	54.5	53.5	4	6	4170.558	12.5	13.5	4	6
4000.622	54.5	53.5	4	6	4170.732	37.5	36.5	4	6
4006.536	40.5	40.5	4	6	4176.164	21.5	20.5	4	6
4022.035	52.5	52.5	4	6	4182.429	13.5	14.5	4	6
4026.485	28.5	29.5	4	6	4183.336	5.5	6.5	4	6
4038.772	27.5	28.5	4	6	4185.031	35.5	34.5	4	6
4046.251	50.5	50.5	4	6	4188.465	21.5	22.5	4	6
4051.489	36.5	35.5	4	6	4188.871	9.5	10.5	4	6
4057.924	49.5	49.5	4	6	4188.942	9.5	9.5	4	6
4065.806	17.5	18.5	4	6	4190.818	4.5	5.5	4	6
4067.618	24.5	24.5	4	6	4190.876	35.5	34.5	4	6
4074.327	34.5	35.5	4	6	4194.276	8.5	9.5	4	6
4074.554	34.5	34.5	4	6	4194.335	8.5	8.5	4	6
4077.787	23.5	24.5	4	6	4195.778	53.5	52.5	4	6
4080.373	47.5	47.5	4	6	4197.919	3.5	4.5	4	6
4087.970	22.5	22.5	4	6	4201.883	19.5	20.5	4	6
4091.175	46.5	46.5	4	6	4202.058	16.5	16.5	4	6
4091.439	46.5	45.5	4	6	4204.950	33.5	33.5	4	6
4096.104	22.5	23.5	4	6	4209.952	31.5	30.5	4	6
4101.456	45.5	44.5	4	6	4211.886	32.5	31.5	4	6
4106.758	21.5	22.5	4	6	4213.763	13.5	13.5	4	6
4109.577	30.5	29.5	4	6	4214.227	17.5	18.5	4	6
4114.698	30.5	30.5	4	6	4215.422	30.5	29.5	4	6
4122.068	43.5	42.5	4	6	4216.988	12.5	12.5	4	6
4126.335	28.5	28.5	4	6	4218.096	31.5	31.5	4	6
4129.690	42.5	41.5	4	6	4218.301	31.5	30.5	4	6
4131.704	42.5	41.5	4	6	4218.912	2.5	2.5	4	6
4132.879	28.5	29.5	4	6	4219.872	11.5	11.5	4	6
4133.066	28.5	28.5	4	6	4219.994	16.5	17.5	4	6
4140.423	10.5	11.5	4	6	4222.405	10.5	10.5	4	6
4141.027	41.5	40.5	4	6	4222.479	10.5	9.5	4	6
4142.076	26.5	26.5	4	6	4224.593	9.5	9.5	4	6
4149.160	15.5	16.5	4	6	4224.650	9.5	8.5	4	6
4156.633	14.5	15.5	4	6	4225.512	15.5	16.5	4	6
4163.380	23.5	23.5	4	6	4226.415	8.5	8.5	4	6
4163.529	23.5	22.5	4	6	4227.881	7.5	7.5	4	6
4163.767	13.5	14.5	4	6	4228.688	7.5	8.5	4	6

Table S6 (continued)

ν (cm^{-1})	J'	J''	v'	v''	ν (cm^{-1})	J'	J''	v'	v''
4228.988	6.5	6.5	4	6	4289.308	8.5	8.5	4	6
4229.621	2.5	1.5	4	6	4296.706	39.5	38.5	4	6
4229.710	5.5	5.5	4	6	4322.134	33.5	32.5	4	6
4230.881	14.5	14.5	4	6	4337.003	27.5	26.5	4	6
4232.388	1.5	0.5	4	6	4338.951	17.5	16.5	4	6
4235.582	6.5	7.5	4	6	4341.297	20.5	19.5	4	6
4235.871	13.5	13.5	4	6	3988.810	19.5	19.5	5	7
4238.531	48.5	47.5	4	6	3990.010	21.5	22.5	5	7
4241.691	24.5	23.5	4	6	3990.618	9.5	9.5	5	7
4244.341	4.5	3.5	4	6	3992.532	4.5	5.5	5	7
4245.128	11.5	11.5	4	6	3994.000	18.5	18.5	5	7
4245.951	26.5	26.5	4	6	3995.912	8.5	9.5	5	7
4247.948	22.5	21.5	4	6	3996.969	20.5	20.5	5	7
4248.734	4.5	5.5	4	6	3998.599	33.5	32.5	5	7
4249.387	10.5	10.5	4	6	3998.984	17.5	16.5	5	7
4250.402	6.5	5.5	4	6	3999.568	3.5	4.5	5	7
4253.408	9.5	9.5	4	6	4003.426	16.5	16.5	5	7
4254.965	8.5	7.5	4	6	4009.689	18.5	19.5	5	7
4256.616	18.5	17.5	4	6	4009.802	5.5	6.5	5	7
4256.693	9.5	8.5	4	6	4015.099	13.5	13.5	5	7
4257.199	8.5	8.5	4	6	4015.828	17.5	17.5	5	7
4258.060	10.5	9.5	4	6	4016.006	9.5	10.5	5	7
4259.086	11.5	10.5	4	6	4017.207	3.5	4.5	5	7
4259.687	15.5	14.5	4	6	4018.397	12.5	11.5	5	7
4259.750	12.5	11.5	4	6	4021.113	29.5	28.5	5	7
4260.066	13.5	12.5	4	6	4021.191	11.5	11.5	5	7
4260.762	7.5	7.5	4	6	4021.271	11.5	10.5	5	7
4263.132	22.5	22.5	4	6	4023.092	1.5	1.5	5	7
4263.274	22.5	21.5	4	6	4027.731	8.5	8.5	5	7
4266.893	1.5	2.5	4	6	4031.028	5.5	5.5	5	7
4267.220	5.5	5.5	4	6	4031.416	4.5	3.5	5	7
4270.047	20.5	20.5	4	6	4032.325	14.5	14.5	5	7
4270.122	4.5	4.5	4	6	4037.239	6.5	7.5	5	7
4275.303	2.5	2.5	4	6	4042.065	12.5	12.5	5	7
4277.593	1.5	1.5	4	6	4043.880	5.5	6.5	5	7
4278.367	17.5	17.5	4	6	4045.521	4.5	3.5	5	7
4278.484	17.5	16.5	4	6	4046.552	11.5	11.5	5	7
4280.603	16.5	16.5	4	6	4050.293	4.5	5.5	5	7
4282.683	15.5	14.5	4	6	4050.727	10.5	11.5	5	7
4287.122	3.5	2.5	4	6	4050.797	10.5	10.5	5	7
4287.905	11.5	11.5	4	6	4053.633	20.5	19.5	5	7
4289.076	9.5	9.5	4	6	4053.931	7.5	6.5	5	7
4289.139	9.5	8.5	4	6	4054.743	9.5	10.5	5	7

Table S6 (continued)

ν (cm^{-1})	J'	J''	v'	v''
4055.653	19.5	18.5	5	7
4055.992	8.5	7.5	5	7
4058.592	8.5	8.5	5	7
4059.025	10.5	9.5	5	7
4060.658	12.5	11.5	5	7
4060.900	14.5	13.5	5	7
4063.997	22.5	21.5	5	7
4067.478	21.5	21.5	5	7
4074.008	19.5	18.5	5	7
4080.964	0.5	1.5	5	7
4081.577	16.5	15.5	5	7
4085.217	14.5	14.5	5	7
4098.884	3.5	2.5	5	7
4103.264	4.5	3.5	5	7
4114.103	35.5	34.5	5	7
4118.614	8.5	7.5	5	7
4127.717	31.5	30.5	5	7
4132.572	13.5	12.5	5	7
4138.190	26.5	25.5	5	7
4140.183	18.5	17.5	5	7

Table S7: Emission lines of the CN radical for Δv = -1 of the $A^2\Pi \rightarrow X^2\Sigma^+$ transition

**ν (cm^{-1})**	**J'**	**J''**	**v'**	**v''**	**ν (cm^{-1})**	**J'**	**J''**	**v'**	**v''**
6484.045	59.5	58.5	0	1	7023.429	31.5	31.5	0	1
6497.467	48.5	48.5	0	1	7027.136	17.5	18.5	0	1
6516.834	47.5	47.5	0	1	7030.587	30.5	30.5	0	1
6535.876	46.5	46.5	0	1	7033.584	16.5	17.5	0	1
6540.721	56.5	56.5	0	1	7034.286	3.5	4.5	0	1
6554.252	45.5	46.5	0	1	7037.141	11.5	10.5	0	1
6572.662	44.5	45.5	0	1	7037.696	2.5	3.5	0	1
6572.987	44.5	44.5	0	1	7049.281	3.5	3.5	0	1
6612.312	52.5	52.5	0	1	7061.508	25.5	25.5	0	1
6660.013	31.5	32.5	0	1	7065.349	21.5	20.5	0	1
6671.620	38.5	39.5	0	1	7066.728	24.5	24.5	0	1
6676.236	38.5	39.5	0	1	7080.639	21.5	20.5	0	1
6694.775	47.5	46.5	0	1	7084.447	20.5	20.5	0	1
6709.873	46.5	46.5	0	1	7108.176	9.5	8.5	0	1
6719.545	45.5	46.5	0	1	7108.350	5.5	4.5	0	1
6744.837	56.5	55.5	0	1	7108.690	6.5	5.5	0	1
6749.835	43.5	44.5	0	1	7108.785	7.5	6.5	0	1
6778.830	41.5	42.5	0	1	7127.004	5.5	4.5	0	1
6781.849	31.5	32.5	0	1	7154.115	14.5	13.5	0	1
6802.489	52.5	51.5	0	1	7159.118	19.5	18.5	0	1
6821.756	38.5	37.5	0	1	6509.645	34.5	35.5	1	2
6832.959	37.5	38.5	0	1	6517.431	26.5	27.5	1	2
6837.893	27.5	28.5	0	1	6536.503	42.5	43.5	1	2
6842.201	49.5	48.5	0	1	6541.139	32.5	32.5	1	2
6858.361	35.5	35.5	0	1	6548.445	24.5	25.5	1	2
6873.259	17.5	18.5	0	1	6564.822	40.5	41.5	1	2
6888.928	45.5	45.5	0	1	6567.760	40.5	39.5	1	2
6915.557	30.5	30.5	0	1	6606.381	20.5	21.5	1	2
6923.090	42.5	41.5	0	1	6630.261	35.5	35.5	1	2
6932.027	57.5	56.5	0	1	6632.565	25.5	25.5	1	2
6935.980	28.5	29.5	0	1	6652.416	33.5	32.5	1	2
6954.723	39.5	38.5	0	1	6676.602	31.5	31.5	1	2
6955.369	26.5	27.5	0	1	6682.759	14.5	15.5	1	2
6956.017	55.5	54.5	0	1	6687.161	30.5	31.5	1	2
6973.671	24.5	24.5	0	1	6697.562	19.5	19.5	1	2
6990.364	22.5	23.5	0	1	6697.645	29.5	30.5	1	2
6998.488	21.5	21.5	0	1	6707.162	18.5	18.5	1	2
6999.946	51.5	50.5	0	1	6713.648	40.5	39.5	1	2
7005.135	9.5	9.5	0	1	6714.879	40.5	40.5	1	2
7006.140	20.5	20.5	0	1	6738.777	16.5	17.5	1	2
7011.029	8.5	8.5	0	1	6741.965	14.5	14.5	1	2
7016.514	7.5	7.5	0	1	6764.172	11.5	11.5	1	2
7020.394	18.5	19.5	0	1	6765.492	20.5	19.5	1	2

Table S7 (continued)

ν (cm^{-1})	J'	J''	v'	v''	ν (cm^{-1})	J'	J''	v'	v''
6770.917	34.5	33.5	1	2	6592.636	5.5	5.5	2	3
6771.747	19.5	18.5	1	2	6597.104	6.5	7.5	2	3
6773.293	33.5	32.5	1	2	6604.278	3.5	2.5	2	3
6777.068	9.5	9.5	1	2	6607.852	4.5	3.5	2	3
6777.728	20.5	21.5	1	2	6610.334	10.5	10.5	2	3
6782.918	8.5	8.5	1	2	6614.763	9.5	9.5	2	3
6788.363	7.5	7.5	1	2	6617.730	17.5	16.5	2	3
6794.151	31.5	31.5	1	2	6619.033	22.5	21.5	2	3
6797.635	14.5	13.5	1	2	6619.265	16.5	15.5	2	3
6801.231	1.5	2.5	1	2	6620.443	15.5	14.5	2	3
6801.594	9.5	10.5	1	2	6621.242	14.5	13.5	2	3
6805.406	12.5	11.5	1	2	6621.707	12.5	11.5	2	3
6805.626	28.5	27.5	1	2	6622.818	7.5	7.5	2	3
6812.362	1.5	1.5	1	2	6623.346	21.5	20.5	2	3
6820.876	3.5	3.5	1	2	6640.189	16.5	16.5	2	3
6821.118	27.5	26.5	1	2	6646.833	0.5	0.5	2	3
6840.622	43.5	42.5	1	2	6652.190	6.5	6.5	2	3
6844.980	3.5	4.5	1	2	6681.350	8.5	7.5	2	3
6847.169	22.5	22.5	1	2	6687.430	10.5	9.5	2	3
6847.459	16.5	15.5	1	2	6693.681	26.5	25.5	2	3
6859.264	19.5	18.5	1	2					
6862.618	18.5	17.5	1	2					
6878.762	10.5	9.5	1	2					
6879.719	5.5	5.5	1	2					
6879.949	8.5	7.5	1	2					
6880.146	7.5	6.5	1	2					
6915.356	29.5	28.5	1	2					
6918.229	28.5	27.5	1	2					
6920.836	12.5	11.5	1	2					
6922.969	26.5	25.5	1	2					
6926.334	15.5	14.5	1	2					
6926.423	24.5	23.5	1	2					
6928.604	22.5	21.5	1	2					
6499.499	10.5	11.5	2	3					
6517.997	24.5	24.5	2	3					
6526.381	23.5	24.5	2	3					
6540.370	13.5	14.5	2	3					
6543.467	10.5	11.5	2	3					
6544.145	19.5	18.5	2	3					
6557.941	11.5	12.5	2	3					
6583.987	10.5	9.5	2	3					
6584.810	1.5	2.5	2	3					
6592.435	25.5	24.5	2	3					

Table S8: Observed emission lines of the CH radical in the $X^2\Pi$ ground state

ν (cm^{-1})	J'	J''	v'	v''	F'	F''	ν (cm^{-1})	J'	J''	v'	v''	F'	F''
2513.872	6.5	7.5	1	0	6	8	2729.863	2.5	2.5	1	0	2	3
2514.197	5.5	6.5	1	0	5	6	2730.183	1.5	1.5	1	0	0	1
2514.428	6.5	7.5	1	0	6	8	2731.926	0.5	0.5	1	0	0	0
2535.198	2.5	3.5	1	0	2	3	2736.074	2.5	1.5	1	0	2	1
2536.167	2.5	3.5	1	0	2	3	2749.537	1.5	0.5	1	0	0	0
2547.568	4.5	5.5	1	0	4	5	2749.674	1.5	0.5	1	0	0	0
2547.937	5.5	6.5	1	0	5	7	2785.113	2.5	1.5	1	0	2	2
2548.079	4.5	5.5	1	0	4	5	2797.010	1.5	0.5	1	0	0	0
2548.409	5.5	6.5	1	0	5	7	2806.756	2.5	2.5	1	0	2	3
2550.550	5.5	5.5	1	0	5	5	2810.799	3.5	2.5	1	0	3	3
2580.646	3.5	4.5	1	0	3	4	2810.996	3.5	2.5	1	0	3	3
2581.193	4.5	5.5	1	0	4	6	2812.968	2.5	1.5	1	0	2	1
2581.583	4.5	5.5	1	0	4	6	2813.204	2.5	1.5	1	0	2	1
2584.140	4.5	4.5	1	0	4	4	2835.242	4.5	3.5	1	0	4	4
2595.962	1.5	2.5	1	0	0	2	2836.115	3.5	2.5	1	0	3	2
2599.821	15.5	15.5	1	0	15	15	2855.856	4.5	4.5	1	0	4	5
2612.718	2.5	3.5	1	0	2	3	2857.813	5.5	4.5	1	0	5	5
2613.062	2.5	3.5	1	0	2	3	2858.141	5.5	4.5	1	0	5	5
2613.613	3.5	4.5	1	0	3	5	2858.538	4.5	3.5	1	0	4	3
2613.914	3.5	4.5	1	0	3	5	2858.885	4.5	3.5	1	0	4	3
2643.436	1.5	2.5	1	0	0	2	2862.009	2.5	1.5	1	0	2	2
2645.247	2.5	3.5	1	0	2	4	2862.504	2.5	1.5	1	0	2	2
2645.404	12.5	12.5	1	0	12	13	2877.906	5.5	5.5	1	0	5	6
2658.778	11.5	11.5	1	0	11	12	2879.754	6.5	5.5	1	0	6	6
2659.520	11.5	10.5	1	0	11	10	2879.883	5.5	4.5	1	0	5	4
2664.883	0.5	1.5	1	0	1	1	2880.283	5.5	4.5	1	0	5	4
2667.875	11.5	11.5	1	0	11	12	2899.653	7.5	6.5	1	0	7	7
2676.357	1.5	2.5	1	0	0	3	2917.844	7.5	7.5	1	0	7	8
2678.687	10.5	10.5	1	0	10	11	2918.928	7.5	6.5	1	0	7	6
2682.487	1.5	1.5	1	0	0	1	2919.102	8.5	7.5	1	0	8	8
2682.711	1.5	1.5	1	0	0	1	2919.424	7.5	6.5	1	0	7	6
2690.858	7.5	8.5	1	0	7	9	2935.753	8.5	8.5	1	0	8	9
2692.158	7.5	7.5	1	0	7	7	2936.285	9.5	8.5	1	0	9	9
2697.364	8.5	8.5	1	0	8	9	2966.456	10.5	10.5	1	0	10	11
2701.386	7.5	7.5	1	0	7	8	2993.358	12.5	12.5	1	0	12	13
2703.722	6.5	7.5	1	0	6	8	3003.078	13.5	13.5	1	0	13	14
2705.242	7.5	7.5	1	0	7	8	3019.696	5.5	4.5	1	0	5	5
2709.129	5.5	5.5	1	0	5	5	3020.854	16.5	15.5	1	0	16	16
2709.264	6.5	6.5	1	0	6	7	3027.096	17.5	16.5	1	0	17	17
2711.189	6.5	5.5	1	0	6	5	3027.819	16.5	15.5	1	0	16	15
2712.420	5.5	5.5	1	0	5	5	3035.017	19.5	18.5	1	0	19	19
2726.181	3.5	3.5	1	0	3	4	2522.374	2.5	3.5	2	1	2	3
2727.193	2.5	2.5	1	0	2	2	2526.297	2.5	2.5	2	1	2	2

Table S8 (continued)

ν (cm^{-1})	J'	J''	v'	v''	F'	F''	ν (cm^{-1})	J'	J''	v'	v''	F'	F''
2552.370	1.5	2.5	2	1	0	2	2616.430	6.5	5.5	3	2	6	5
2552.504	1.5	2.5	2	1	0	2	2616.778	6.5	5.5	3	2	6	5
2571.869	7.5	7.5	2	1	7	7	2616.921	5.5	4.5	3	2	5	4
2586.501	5.5	5.5	2	1	5	5	2634.736	7.5	6.5	3	2	7	6
2595.727	3.5	3.5	2	1	3	3	2635.120	7.5	6.5	3	2	7	6
2600.191	3.5	3.5	2	1	3	3	2652.187	8.5	7.5	3	2	8	7
2600.894	3.5	3.5	2	1	3	3							
2601.181	2.5	2.5	2	1	2	2							
2603.707	1.5	1.5	2	1	0	0							
2604.164	1.5	1.5	2	1	0	0							
2605.884	0.5	0.5	2	1	0	1							
2623.561	1.5	0.5	2	1	0	1							
2669.003	1.5	0.5	2	1	0	1							
2669.120	1.5	0.5	2	1	0	0							
2678.077	2.5	2.5	2	1	2	2							
2702.253	3.5	3.5	2	1	3	3							
2726.617	5.5	4.5	2	1	5	4							
2726.924	5.5	4.5	2	1	5	4							
2727.327	4.5	3.5	2	1	4	3							
2727.666	4.5	3.5	2	1	4	3							
2730.963	2.5	1.5	2	1	2	0							
2747.175	6.5	5.5	2	1	6	5							
2747.539	6.5	5.5	2	1	6	5							
2748.065	5.5	4.5	2	1	5	4							
2783.013	7.5	7.5	2	1	7	7							
2784.470	8.5	7.5	2	1	8	7							
2785.246	7.5	6.5	2	1	7	6							
2801.156	9.5	8.5	2	1	9	8							
2801.396	8.5	7.5	2	1	8	7							
2801.919	8.5	7.5	2	1	8	7							
2843.136	12.5	11.5	2	1	12	11							
2864.241	13.5	12.5	2	1	13	12							
2880.032	15.5	14.5	2	1	15	14							
2530.244	2.5	1.5	3	2	2	0							
2553.943	3.5	2.5	3	2	3	2							
2554.127	3.5	2.5	3	2	3	2							
2555.986	2.5	1.5	3	2	2	0							
2576.067	4.5	3.5	3	2	4	3							
2576.310	4.5	3.5	3	2	4	3							
2577.140	3.5	2.5	3	2	3	2							
2577.418	3.5	2.5	3	2	3	2							
2596.883	5.5	4.5	3	2	5	4							
2597.179	5.5	4.5	3	2	5	4							
